# Cediranib with weekly paclitaxel or olaparib versus weekly paclitaxel for advanced or recurrent endometrial cancer (COPELIA): a multicentre, open-label, randomised, phase 2 trial in the UK

**DOI:** 10.1016/j.eclinm.2026.104012

**Published:** 2026-06-16

**Authors:** Robert D. Morgan, Catharine Porter, Cong Zhou, Rebecca Kristeleit, Sudha Desai, Ignacio Vazquez, Angela George, Gemma Eminowicz, Axel Walther, Adrian Franklin, Azmat H. Sadozye, Andrew Hughes, Louise Hanna, Rene Roux, Rosemary Lord, Rebecca Bowen, Joey Wood, Clara Sentamans, Ann White, Angela C. Casbard, Alys Humphrys, Jennifer B. Swettenham, Lisette S. Nixon, Richard Adams, Ruby Ray, Elena Brogden, Wendy E. Powell, Joanna Canham, Tracy M. Cox, Monica Narasimham, Helen Lowe, Leah Ensell, Mohammed Zubair, Karen Morris, Molly Glenister-Doyle, Ariadna Fuertes Gassio, Derrick Morgan, Dylan Broughton, Margherita Carucci, Phillip J. Monaghan, Jonathan Tugwood, Richard J. Edmondson, Caroline Dive, Gordon C. Jayson, Andrew R. Clamp

**Affiliations:** a The University of Manchester, Manchester, England; b The Christie NHS Foundation Trust, Manchester, England; c Centre for Trials Research, Cardiff University, Cardiff, Wales, United Kingdom; d Cancer Research UK National Biomarker Centre, Manchester, England; e Guy's and St Thomas' NHS Foundation Trust, London, England; f East and North Hertfordshire NHS Trust, Stevenage, England; g The Royal Marsden NHS Foundation Trust, London, England; h University College London Hospitals NHS Foundation Trust, London, England; i University Hospitals Bristol NHS Foundation Trust, Bristol, England; j Royal Surrey County Hospital NHS Foundation Trust, Guildford, England; k The Beatson West of Scotland Cancer Centre, Glasgow, Scotland; l Newcastle Hospitals NHS Foundation Trust, Newcastle, England; m Velindre University NHS Trust, Cardiff, Wales, United Kingdom; n Oxford University Hospitals NHS Foundation Trust, Oxford, England; o Wirral University Teaching Hospital NHS Foundation Trust, Wirral, England; p Royal United Hospital Bath NHS Foundation Trust, Bath, England; q University Hospitals of Leicester NHS Trust, Leicester, England; r Airedale NHS Foundation Trust, Keighley, England; s UCL ECMC GCLP Facility, University College London, London, England; t Christie Pathology Partnership, Manchester, England; u Manchester University NHS Foundation Trust, Manchester, England

**Keywords:** Clinical trial, Endometrial cancer, Paclitaxel, Cediranib, Olaparib, Plasma Tie2

## Abstract

**Background:**

Patients with advanced or recurrent endometrial cancer have poor survival outcomes because effective treatment options remain limited. We investigated the efficacy and safety of cediranib plus weekly paclitaxel or olaparib versus weekly paclitaxel alone after prior platinum-based chemotherapy.

**Methods:**

COPELIA was an open-label, randomised, phase 2 trial undertaken at 15 centres in the United Kingdom. Eligible participants were aged 16 years or older with histologically confirmed endometrial cancer, Eastern Cooperative Oncology Group performance status 0–1, a life expectancy greater than 16 weeks, and at least one previous line of platinum-based chemotherapy. Patients were randomly assigned to paclitaxel 80 mg/m^2^ on days 1, 8, and 15 of a 28-day cycle (arm 1, control); cediranib 20 mg daily plus the same paclitaxel schedule (arm 2, experimental); or cediranib 20 mg daily plus olaparib 300 mg twice daily (arm 3, experimental). Up to six cycles of paclitaxel were permitted. Patients in arm 2 with a Response Evaluation Criteria in Solid Tumours (RECIST) version 1.1 complete or partial response, or stable disease, after paclitaxel plus cediranib continued cediranib monotherapy. The primary endpoint was the proportion of patients free from RECIST-defined progression or death at 3 months (PFS at 3 months). Secondary endpoints included RECIST response, PFS at 6 months, median PFS, median overall survival (OS), safety, and quality of life. Translational analyses assessed vascular response, defined as a reduction of 5% or more in plasma Tie2 within 9 weeks of treatment initiation. The trial was registered with EudraCT (2016-004617-28), ISRCTN (16320634), and ClinicalTrials.gov (NCT03570437).

**Findings:**

Between May 1, 2018, and January 11, 2022, 124 patients were enrolled and randomised to arm 1 (n = 41), arm 2 (n = 41), or arm 3 (n = 42). Median follow-up in arms 1, 2, and 3 was 34.4 months (interquartile range [IQR] 11.6–37.6), 26.3 months (11.8–not evaluable), and 23.7 months (15.0–not evaluable), respectively. PFS at 3 months was significantly higher in arm 2 than in arm 1 (73.2% versus 48.8%; adjusted odds ratio [aOR] 3.2, lower limit of one-sided 80% confidence interval [CI] 2.1; p = 0.01). RECIST response was also higher in arm 2 versus arm 1 (56.4% versus 28.2%; aOR 5.7, 95% CI 1.8–17.6; p < 0.001). No differences were observed between arm 3 and arm 1 for PFS at 3 months (aOR 1.0, lower limit of one-sided 80% CI 0.71; p = 0.46) or RECIST response (aOR 0.8, 95% CI 0.3–2.6; p = 0.73). Median OS was 12.8 months (95% CI 7.3–16.8) in arm 1, 18.1 months (9.5–26.4; log-rank p = 0.42 versus arm 1) in arm 2, and 13.9 months (11.2–18.3; log-rank p = 0.86 versus arm 1) in arm 3. There was no difference in PFS at 6 months and median PFS between arm 1 and arm 2 or arm 3. Grade 3 adverse events occurring in ≥10% of patients in arm 2 included hypertension (15%), neutropenia (12%), and diarrhoea (10%). Quality-of-life differences favoured arm 1 over arm 2 for diarrhoea and gastrointestinal symptoms (p < 0.001). In multivariable analyses, cediranib-treated patients who achieved a Tie2-defined vascular response had significantly improved PFS (hazard ratio 0.54, 95% CI 0.33–0.88; p = 0.014).

**Interpretation:**

Paclitaxel plus cediranib improved 3-month PFS and RECIST response compared with paclitaxel alone in advanced or recurrent endometrial cancer. However, this effect diminished over time; with no difference in PFS observed at 6 months, nor median PFS or median OS. Plasma Tie2 identified cediranib-treated patients with improved PFS. Further evaluation of paclitaxel plus cediranib in patients previously treated with platinum-based chemotherapy and immunotherapy is warranted, ideally incorporating plasma Tie2 as a response biomarker to guide treatment continuation.

**Funding:**

AstraZeneca.

**Research in context**

**Evidence before this study**

Phase 2 trials have shown that several anti-angiogenic agents, including cediranib, have activity as monotherapy in endometrial cancer. Two phase 2 studies have also demonstrated that weekly paclitaxel (80 mg/m^2^ weekly for 3 weeks of a 4-week cycle) is active in this setting. We searched PubMed from database inception to December 1, 2017, using the terms ‘endometrial cancer’, ‘uterine carcinosarcoma’, ‘cediranib’, and ‘paclitaxel’ (English-language only) to identify studies evaluating paclitaxel combined with cediranib in endometrial cancer; no studies were identified.

**Added value of this study**

In this multicentre, randomised, phase 2 trial, 124 women with advanced or recurrent endometrial cancer received weekly paclitaxel, weekly paclitaxel plus once-daily cediranib, or cediranib plus olaparib. Paclitaxel plus cediranib significantly improved PFS at 3 months and RECIST response compared with paclitaxel alone. However, this effect diminished over time; with no difference in PFS observed at 6 months, nor median PFS or median OS. Quality-of-life scores for diarrhoea and gastrointestinal symptoms were worse with paclitaxel plus cediranib. Cediranib-associated reductions in plasma Tie2 were associated with improved PFS, and vascular non-responders experienced similar toxicity to vascular responders.

**Implications of all the available evidence**

Weekly paclitaxel and cediranib each have activity as monotherapy in endometrial cancer, and this study shows that their combination improves short-term disease control. A logical next step is a clinical trial evaluating a prolonged course of paclitaxel plus cediranib, given that the efficacy signal appears to diminish once paclitaxel is discontinued, in patients with MMR-proficient tumours who have progressed after platinum-based chemotherapy and immunotherapy. Incorporating plasma Tie2 as a biomarker to guide treatment continuation warrants prospective evaluation.

## Introduction

Endometrial cancer is the second most common gynaecological malignancy worldwide, with more than 420,000 cases diagnosed annually.^1^ Most patients are diagnosed with local or locoregional disease and can be cured with surgery with or without radiotherapy.^2^ Approximately 30% of patients present with distant metastases or develop recurrent disease after first-line therapy, resulting in a 5-year survival rate of only 20%. Until the recent introduction of anti-PD-1/PD-L1 immunotherapy, systemic therapy was limited to hormone and chemotherapy, with modest response rates and limited survival benefit.

Cediranib is an orally bioavailable, selective inhibitor of multiple tyrosine kinases involved in tumour angiogenesis including VEGF receptor-1, -2 and -3, c-Kit and platelet-derived growth factor receptors-α and -β.^3^ Olaparib is an orally bioavailable, selective inhibitor of poly(ADP-ribose) polymerases-1/2 (PARP).^4^ At the time COPELIA was initiated, cediranib and several other anti-angiogenic agents had shown activity as monotherapy in phase 2 trials of endometrial cancer.^5^, ^6^, ^7^, ^8^, ^9^, ^10^, ^11^, ^12^, ^13^ In addition, three anti-angiogenic agents, bevacizumab, trebananib, and pazopanib had demonstrated efficacy in phase 2 or 3 trials when combined with weekly paclitaxel in ovarian cancer.^14^, ^15^, ^16^

PARP inhibitors were also known to be synthetically lethal in cells with loss of PTEN expression.^17^^,^^18^ Given that approximately 60% of endometrial cancers contain a mutation in *PTEN*, PARP inhibitors offered a promising therapeutic option.^19^, ^20^, ^21^ Additionally, the combination of olaparib with cediranib had shown efficacy in recurrent ovarian cancer, with the greatest benefit reported in patients without a *BRCA1/2* mutation.^22^ Together, these data supported investigating cediranib with weekly paclitaxel and cediranib with olaparib in endometrial cancer.

The aim of our study was to evaluate the efficacy and safety of these regimens compared with standard chemotherapy in women with advanced or recurrent endometrial cancer. In the absence of a standard second-line or subsequent chemotherapy at the time the trial was initiated, weekly paclitaxel was selected due to its tolerable side effect profile and supportive phase 2 trial data.^23^, ^24^, ^25^

In previous studies investigating ovarian, colorectal and biliary tract cancer, we showed that reductions in plasma Tie2 after treatment with a VEGF inhibitor and chemotherapy were associated with improved progression-free survival (PFS) benefit (hazard ratio [HR] of 0.55–0.70).^26^, ^27^, ^28^, ^29^ A further aim of our study was therefore to explore the clinical significance of changes in plasma Tie2 as a vascular response biomarker for cediranib in endometrial cancer.

## Methods

### Study design and ethics

COPELIA was a randomised, controlled, three-arm, open-label, parallel group, multi-arm multi-stage trial in which patients were enrolled from 15 centres in the UK (Appendix p 3). The trial was registered with EudraCT (2016-004617-28), ISRCTN (16320634) and ClinicalTrials.gov (NCT03570437).

All patients provided written informed consent. Ethical approval for the study was granted by the South Central–Oxford B Research Ethics Committee (Health Research Authority). The study was approved under REC reference 17/SC/0536, IRAS project ID 216069, and MHRA authorisation 35030/0007/001-0001. The trial was performed in accordance with Good Clinical Practice guidelines, provisions of the Declaration of Helsinki and UK clinical trial regulations.

### Participants

All participants had a diagnosis of endometrial cancer and were biologically female at birth. All had previously received platinum-based chemotherapy in the adjuvant setting or for recurrent disease and either had recurrent disease within 18 months of completing adjuvant chemotherapy without having received cytotoxic chemotherapy for recurrence or had received one to two prior lines of cytotoxic chemotherapy for recurrent disease. No patient had received prior dose-dense weekly paclitaxel, a VEGF inhibitor, or a PARP inhibitor. Full eligibility criteria are provided in the supplementary data (Appendix pp 4 and 5).

### Randomisation and masking

Eligible patients were randomly assigned (1:1:1) using a minimisation method with a random element to one of the three treatment arms. The minimisation was stratified by histological subtype including histology grade and number of prior lines of cytotoxic chemotherapy not including adjuvant chemotherapy. At the time COPELIA was designed, molecular subtyping for *POLE*, mismatch repair (MMR) and p53 was not standard practice and these parameters were not included in the stratification.^23^^,^^24^ Randomisation was performed centrally by the Centre for Trials Research (Cardiff University, Wales) using a secure online system, ensuring allocation concealment. Patients and clinicians were not blinded to the assigned treatment.

### Procedures

The investigational medicinal products were cediranib and olaparib. Patients in arm 1 received intravenous paclitaxel 80 mg/m^2^, dosed by body-surface area, on days 1, 8, and 15 of each 28-day cycle for a maximum of six cycles. Patients in arm 2 received cediranib 20 mg once daily and intravenous paclitaxel 80 mg/m^2^ on days 1, 8, and 15 of each 28-day cycle for up to six cycles. Patients in arm 2 with a Response Evaluation Criteria in Solid Tumours (RECIST) version 1.1 complete or partial response, or stable disease, at the end of paclitaxel treatment could continue cediranib until disease progression. In arm 3, patients received cediranib 20 mg once daily and olaparib 300 mg (tablets) twice daily until disease progression.

Dose levels for each drug are provided in the supplementary material (Appendix p 6). Protocol-specified indications for dose modifications and treatment discontinuation are detailed in the supplementary material (Appendix p 7). If trial treatment was interrupted for more than four consecutive weeks, the patient was withdrawn from the trial. All trial treatment was discontinued following radiological progression according to RECIST, clinical progression, unacceptable toxicity, non-compliance, or withdrawal of consent.

### Outcomes

The primary endpoint was progression-free survival at 3 months (PFS at 3 months). PFS at 3 months was defined as radiological progression, assessed by the investigator according to RECIST, or death, whichever occurred first. Patients were assessed by computed tomography of the thorax, abdomen, and pelvis within 28 days before day 1 of cycle 1 (pre-treatment scan), at 6 and 12 weeks, and then every 12 weeks thereafter. Disease progression was assessed by comparing the pre-treatment scan with the 6-week and 12-week scans.

Secondary endpoints included the RECIST response rate, PFS at 6 months, median PFS, median OS, safety, and quality of life. PFS at 6 months was defined as radiological progression, assessed by the investigator according to RECIST, or death, whichever occurred first. PFS was measured from the date of randomisation to the date of disease progression or death, whichever occurred first. OS was measured from the date of randomisation to the date of death; patients alive at data lock were censored at the date last seen. Safety was assessed by evaluating adverse events, which were graded using the National Cancer Institute Common Terminology Criteria for Adverse Events version 4.03. Quality of life was measured using the European Organisation for Research and Treatment of Cancer (EORTC) Quality of Life Questionnaire-Core 30 (QLQ-C30) and the Endometrial Cancer Module (EN24). Quality-of-life data were collected at screening, on day 1 of each new cycle, and at the end-of-trial visit. Patients were followed until disease progression, complete withdrawal, or death.

All translational research endpoints were exploratory. Patients could optionally donate research blood samples for plasma Tie2 analysis (Appendix pp 9 and 10). Blood samples were collected at the screening visit and on day 1 of cycles 1, 2, and 3. The screening and day 1 cycle 1 values were averaged to define the pre-treatment plasma Tie2 value for each patient. Vascular response was determined by comparing the pre-treatment Tie2 value with the day 1 cycle 3 value. Based on previously published methodology, a reduction in plasma Tie2 of 5% or more within the first 9 weeks of treatment was defined as a vascular response.^29^ Patients treated with cediranib who attained this reduction were classified as vascular responders and were hypothesised to derive clinical benefit from cediranib, whereas those who did not meet this threshold were classified as vascular non-responders. Patients could also optionally donate research blood samples for circulating tumour cell (CTC) analysis on day 1 of cycle 1 (Appendix pp 11 and 12).

### Statistical analysis

The trial adopted a multi-arm, multi-stage design, allowing one experimental arm (arm 2 or arm 3) to be dropped, or the trial to be stopped early, for a lack of benefit at the predefined interim analysis of the primary endpoint. The predicted median PFS for patients receiving weekly paclitaxel (arm 1) was 3.0 months, corresponding to an anticipated 3-month PFS of 50%. The study was powered to detect an improvement in either experimental arm from 50% to 70%. The interim analysis was designed with a nominal alpha of 0.50 and 90% power, and the final analysis with a nominal alpha of 0.20 and 85% power. The design required an interim analysis when 19 patients (with primary outcome data available) were recruited in each arm (57 in total) and a final analysis when 41 patients per arm were recruited (123 in total). Allowing for 5% loss to follow-up, a total of 129 patients were planned for recruitment, with the interim analysis planned after 20 patients in each arm (60 in total). The final analysis was planned once all patients had met at least one of the criteria: completed 12 months of follow-up post-treatment, had been withdrawn from follow-up, had been lost to follow-up, had experienced disease progression, or had died.

The primary analysis followed the intention-to-treat principle, with no imputation of missing data as pre-specified in the protocol. All analyses used observed data only, no imputation was required for secondary endpoints. The primary endpoint (PFS at 3 months) in each trial arm was compared using multivariable logistic regression adjusting for stratification factors, applying a one-sided significance level of 0.20 and reporting one-sided 80% confidence intervals (CIs). No sensitivity analyses were performed. A per-protocol sensitivity analysis of the primary analysis was considered, but was not feasible due to resource constraints. PFS and OS were compared using a two-sided log-rank test. Median PFS, OS and follow-up intervals were estimated from the 50th percentile of the corresponding Kaplan–Meier estimates. Cox proportional hazards regression analyses were used to estimate hazard ratios (adjusted for stratification factors) for PFS and OS. Proportionality and linearity were assessed using post-estimation tests and Schoenfeld residual plots in Stata 19. The RECIST response rate was analysed using logistic regression adjusting for stratification factors, with results presented as adjusted odds ratios and two-sided 95% CIs. The safety analysis included all patients who received at least one dose of trial treatment. Linear mixed-effects models were used to compare trial arms for each subscale and single item of the EORTC QLQ-C30 and EN24 questionnaires, and time-by-treatment interaction p-values were calculated. No adjustment was made for multiplicity in the analysis of quality-of-life endpoints. No differences in any endpoints were explored between recruiting centres. All primary and secondary endpoint analyses were pre-specified.

A post-hoc analysis was performed to assess the impact of molecular subtypes on the primary endpoint (PFS at 3 months). The analysis included all patients who had consented to optional donation of archival tumour tissue for research. Tumours with loss of expression of one or more MMR proteins were classified as MMR-deficient (dMMR) (Appendix p 8). Tumours with normal expression of all MMR proteins and mutant p53 expression were classified as p53-abnormal (p53abn). Tumours with normal MMR and p53 expression were classified as no specific molecular profile (NSMP). A *POLE* test was not available in our central research laboratory at the time of testing.

In the translational research analyses, continuous variables such as age were dichotomised by their median values. The association between two clinical variables or between a clinical variable and the primary endpoint (PFS at 3 months) were assessed using chi-squared tests. A multivariable logistic regression analysis was applied to interrogate the impact of clinical variables, including the molecular subtypes, on the primary endpoint. A backward stepwise method was used for model development. The difference in plasma Tie2 values between pre-treatment and day 1 cycle 3 samples was compared across three groups (patients receiving paclitaxel alone, those showing a Tie2 response to cediranib, and those without a Tie2 response), using a Kruskal–Wallis test. Missing data at day 1 cycle 3 were extrapolated if a patient received at least three cycles of treatment and the pre-treatment and day 1 cycle 2 concentrations were available. The survival benefit of Tie2-defined vascular response was assessed using multivariable Cox proportional hazard regression analysis on PFS and OS following the REMARK guideline.^30^ The impact of pre-treatment CTC counts on PFS and OS were assessed using multivariable Cox proportional hazard regression analysis. All translational research analyses were post-hoc.

### Role of the funding source

The funder supplied olaparib and cediranib and provided feedback on the study design, but had no role in the collection, analysis, or interpretation of the data, or in the writing of the trial report or manuscript.

## Results

**Fig. 1:**
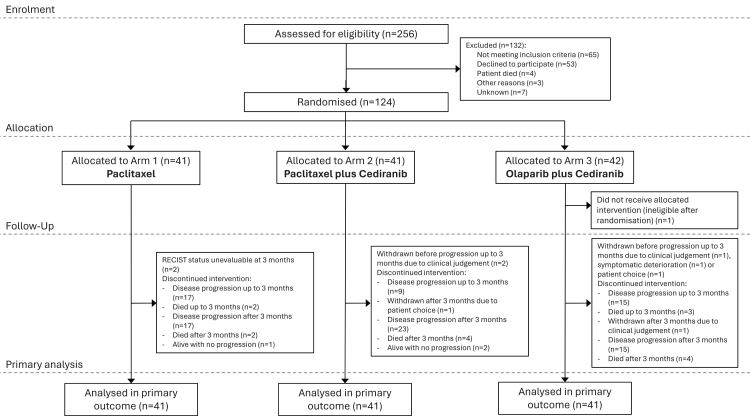
**Trial profile**.

Between May 1, 2018, and Jan 11, 2022, 124 patients were enrolled and randomly assigned to receive paclitaxel (n = 41), paclitaxel plus cediranib (n = 41), or olaparib plus cediranib (n = 42) (Fig. 1). Baseline demographic and disease characteristics are shown in Table 1. One patient in the olaparib plus cediranib arm was found to be ineligible after randomisation and withdrew before receiving treatment; this patient was excluded from the primary analysis. Treatment duration and dose intensity are provided in the supplementary material (Appendix p 13). The data cutoff was April 29, 2023, by which time no patient remained on trial treatment. The most common reason for treatment discontinuation in all arms was disease progression. Median follow-up in arms 1, 2, and 3 was 34.4 months (interquartile range [IQR] 11.6–37.6), 26.3 months (IQR 11.8–not evaluable), and 23.7 months (IQR 15.0–not evaluable), respectively.

**Table 1:** Baseline demographic data and disease characteristics.

	Arm 1 paclitaxel	Arm 2 paclitaxel plus cediranib	Arm 3 olaparib plus cediranib
**Age/years**			
Median	68.1	66.0	66.3
Interquartile range	61.1–73.3	58.8–70.3	60.2–70.4
**ECOG performance status**			
0	17 (41%)	25 (61%)	24 (57%)
1	24 (59%)	16 (39%)	18 (43%)
**Histology**			
Endometrioid	21 (51%)	21 (51%)	17 (40%)
Low grade (grade 1/2)	6	9	10
High-grade (grade 3)	14	12	7
Unknown grade	1	0	0
Serous	10 (24%)	14 (34%)	18 (43%)
Clear cell	3 (7%)	2 (5%)	1 (2%)
Carcinosarcoma	7 (17%)	4 (10%)	6 (14%)
**FIGO stage at diagnosis**			
I	9 (22%)	9 (22%)	8 (19%)
II	4 (10%)	6 (15%)	3 (7%)
III	12 (29%)	12 (29%)	12 (29%)
IV	15 (37%)	13 (32%)	19 (45%)
Unknown	1 (2%)	1 (2%)	0
**Prior surgery**	32 (78%)	33 (80%)	31 (74%)
**Prior radiotherapy**	27 (66%)	25 (61%)	24 (57%)
**Prior lines of cytotoxic chemotherapy not including adjuvant chemotherapy**			
0	8 (20%)	7 (17%)	8 (19%)
1	29 (71%)	30 (73%)	29 (69%)
2	4 (10%)	4 (10%)	5 (12%)

Key: ECOG, Eastern Cooperative Oncology Group; FIGO, International Federation of Gynecology and Obstetrics.

Data are presented as number (percent) unless otherwise stated.

The primary analysis showed that the proportion of patients who were free from progression at 3 months was significantly higher in the paclitaxel plus cediranib arm than in the paclitaxel arm (73.2% versus 48.8%; adjusted odds ratio [OR] 3.2; lower limit of the one-sided 80% CI 2.1; p = 0.01; Table 2). There was no significant difference in PFS at 3 months between the olaparib plus cediranib arm and the paclitaxel arm (48.8% versus 48.8%; adjusted OR 1.0; lower limit of the one-sided 80% CI 0.71; p = 0.46; Table 2).

**Table 2:** Efficacy outcomes.

Efficacy outcomes	Arm 1 Paclitaxel	Arm 2 Paclitaxel plus cediranib	Arm 3 Olaparib plus cediranib
PFS at 3 months (primary endpoint)			
ITT population	41	41	41
No evidence of progression or death at 3 months (%)	20 (48.8%)	30 (73.2%)	20 (48.8%)
Unadjusted difference in proportions (80% CI; p-value)	–	−0.26 (−0.17; p = 0.01)	−0.013 (−0.082; p = 0.45)
Adjusted odds ratio (80% CI; p-value)	–	3.2 (2.1; p = 0.01)	1.0 (0.71; p = 0.46)
RECIST response			
RECIST evaluable	39	39	39
Complete or partial response % (95% CI)	28.2% (16.0–44.7%)	56.4% (40.2–71.3%)	23.1% (12.2–39.3%)
Adjusted odds ratio (95% CI; p-value)	–	5.7 (1.8–17.6; p < 0.001)	0.8 (0.3–2.6; p = 0.73)
PFS at 6 months			
Survival status known at 6 months	38	36	36
No evidence of progression or death at 6 months (%)	15 (39.5%)	16 (44.4%)	13 (36.1%)
Adjusted odds ratio (80% CI; p-value)	–	1.6 (1.0; p = 0.19)	0.70 (0.44; p = 0.26)
Progression-free survival			
Events (progression or death)	38	36	37
Median PFS, months (95% CI)	5.4 (1.6–7.7)	6.9 (5.4–8.3)	4.3 (2.7–8.3)
Log-rank test p-value	–	p = 0.11	p = 0.49
Adjusted hazard ratio (95% CI; p-value)	–	0.72 (0.45–1.15; p = 0.16)	0.94 (0.75–1.18; p = 0.60)
Overall survival			
Events (deaths)	27	26	30
Median OS, months (95% CI)	12.8 (7.3–16.8)	18.1 (9.5–26.4)	13.9 (11.2–18.3)
Log-rank test p-value	–	p = 0.42	p = 0.86
Adjusted hazard ratio (95% CI; p-value)	–	0.82 (0.47–1.41; p = 0.47)	1.00 (0.76–1.31; p = 0.99)

Key: CI, confidence interval; ITT, intention to treat.

**Fig. 2:**
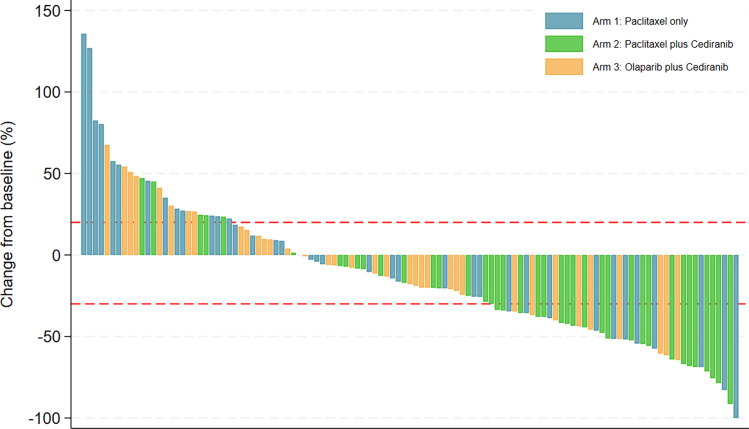
**Waterfall plot showing percentage change in RECIST marker lesions from baseline**. Dashed red lines represent 20% increase and 30% decrease in RECIST marker lesions from baseline.

No imputation for missing data was required for secondary endpoints. RECIST response could be assessed in 117 patients (39 per arm). A waterfall plot of best percentage change in target lesions is shown in Fig. 2. The RECIST response rates in arms 1, 2, and 3 were 28.2% (11/39; including 2 complete responses), 56.4% (22/39; all partial responses), and 23.1% (9/39; all partial responses), respectively (Table 2). After adjustment for stratification factors, the odds of achieving a RECIST response were 5.7 times higher with paclitaxel plus cediranib than with paclitaxel alone (OR 5.7, 95% CI 1.8–17.6; p < 0.001; Table 2). There was no significant difference in RECIST response between the olaparib plus cediranib arm and the paclitaxel arm (OR 0.8, 95% CI 0.3–2.6; p = 0.73; Table 2).

**Fig. 3:**
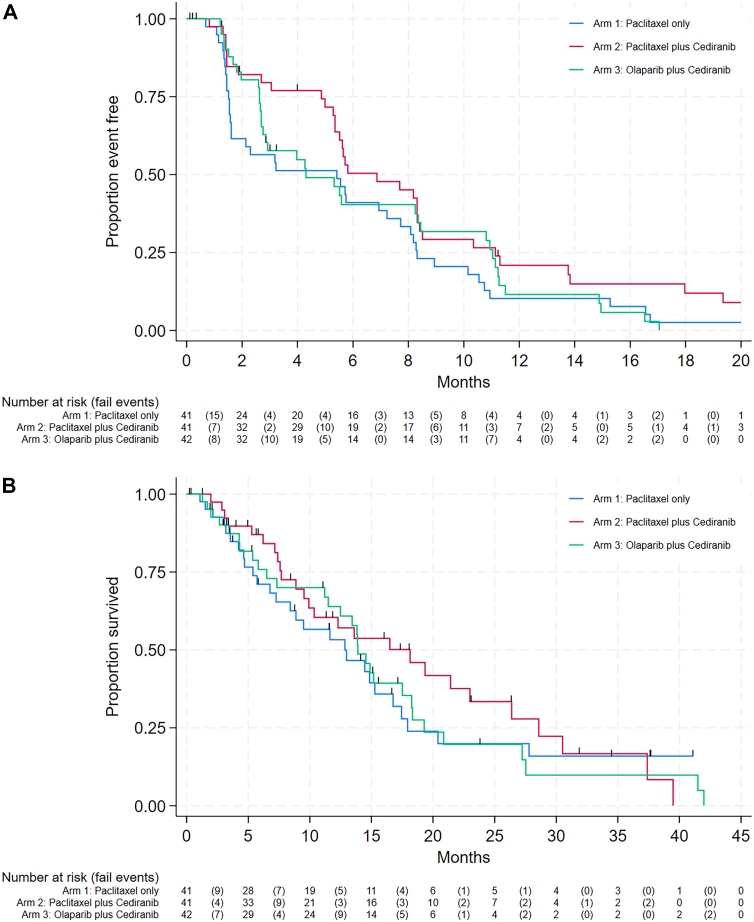
**Kaplan–Meier estimates of (A) progression-free survival and (B) overall survival**.

There was no significant difference in PFS at 6 months, median PFS, or median OS between arm 1 and arm 2 or arm 3 (Table 2). Kaplan–Meier curves for PFS and OS are shown in Fig. 3. There were 111 PFS events (89.5% data maturity). Median PFS in arms 1, 2, and 3 was 5.4 months (95% CI 1.6–7.7), 6.9 months (95% CI 5.4–8.3), and 4.3 months (95% CI 2.7–8.3), respectively. There were 83 deaths (66.9% data maturity). Median OS in arms 1, 2, and 3 was 12.8 months (95% CI 7.3–16.8), 18.1 months (95% CI 9.5–26.4), and 13.9 months (95% CI 11.2–18.3), respectively. Although median OS was 5.3 months longer with paclitaxel plus cediranib than with paclitaxel alone, the 95% CIs overlapped and the log-rank p-value was not statistically significant (p = 0.42).

The safety analysis included 123 patients (41 per arm). All adverse events are reported in the supplementary material (Appendix pp 14–26). Adverse events occurring in 10% or more of patients are shown in Table 3. Significantly more patients experienced a grade 3 adverse event in arm 2 (59%; p < 0.001) and arm 3 (46%; p = 0.02) than in arm 1 (22%). Grade 3 hypertension occurred only in patients treated with cediranib (six in arm 2 and one in arm 3). Three patients treated with cediranib developed a gastrointestinal perforation (two grade 2 and one grade 3), and two patients experienced an arterial thromboembolic event (one grade 2 transient ischaemic attack and one grade 3 cerebrovascular accident); none of these events occurred in arm 1. One patient in arm 1 developed a grade 3 fistula.

**Table 3:** Treatment-related adverse events reported in 10% or more of patients.

Adverse event	Arm 1 paclitaxel			Arm 2 paclitaxel plus cediranib			Arm 3 olaparib plus cediranib		
	Grade 1/2	Grade 3	Grade 4	Grade 1/2	Grade 3	Grade 4	Grade 1/2	Grade 3	Grade 4
**Any event**	**37 (90%)**	**9 (22%)**	**0**	**40 (98%)**	**24 (59%)**	**1 (2%)**	**39 (95%)**	**19 (46%)**	**1 (2%)**
Fatigue	25 (61%)	2 (5%)	0	29 (71%)	3 (7%)	0	27 (64%)	4 (10%)	0
Peripheral sensory neuropathy	20 (49%)	0	0	19 (46%)	0	0	6 (14%)	0	0
Nausea	19 (46%)	0	0	18 (44%)	2 (5%)	0	30 (71%)	0	0
Anaemia	16 (39%)	1 (2%)	0	9 (22%)	1 (2%)	0	10 (24%)	3 (7%)	0
Alopecia	14 (34%)	0	0	14 (34%)	1 (2%)	0	2 (5%)	0	0
Dyspnoea	14 (34%)	1 (2%)	0	16 (39%)	1 (2%)	0	19 (45%)	0	0
Anorexia	13 (32%)	0	0	14 (34%)	0	0	20 (48%)	2 (5%)	0
Constipation	12 (29%)	0	0	18 (44%)	0	0	23 (55%)	0	0
Abdominal pain	10 (24%)	0	0	18 (44%)	3 (7%)	0	18 (43%)	1 (2%)	0
Diarrhoea	10 (24%)	0	0	30 (73%)	3 (10%)	0	30 (73%)	0	0
Back pain	7 (17%)	0	0	6 (15%)	0	0	11 (26%)	1 (2%)	0
Arthralgia	6 (15%)	0	0	6 (15%)	0	0	3 (7%)	1 (2%)	0
Pain	6 (15%)	0	0	5 (12%)	0	0	4 (10%)	0	0
Vomiting	6 (15%)	0	0	11 (27%)	1 (2%)	0	18 (43%)	0	0
Cough	5 (12%)	1 (2%)	0	8 (20%)	0	0	9 (21%)	0	0
Hypertension	5 (12%)	0	0	13 (32%)	6 (15%)	0	9 (21%)	1 (2%)	0
Neutrophil count decreased	5 (12%)	2 (5%)	0	6 (15%)	5 (12%)	0	3 (7%)	0	0
Pain in extremity	5 (12%)	0	0	3 (7%)	0	0	4 (10%)	0	0
Urinary tract infection	4 (10%)	2 (5%)	0	7 (17%)	2 (5%)	0	9 (21%)	0	0
Dysgeusia	3 (7%)	0	0	5 (12%)	0	0	2 (5%)	0	0
Dyspepsia	3 (7%)	0	0	8 (20%)	0	0	10 (24%)	0	0
Alkaline phosphatase increased	2 (5%)	0	0	6 (15%)	0	0	2 (5%)	0	0
Mucositis oral	2 (5%)	0	0	12 (29%)	1 (2%)	0	6 (14%)	0	0
Creatinine increased	1 (2%)	0	0	6 (15%)	0	0	7 (17%)	0	0
Dry skin	1 (2%)	0	0	5 (12%)	0	0	1 (2%)	0	0
Dry mouth	0	0	0	5 (12%)	0	0	2 (5%)	0	0
Pruritus	0	0	0	5 (12%)	0	0	1 (2%)	0	0

Key: Grade 4 adverse events were gastrointestinal perforation (arm 2) and agitation (arm 3).

Data is presented as number of patients with the adverse events (percentage, where the denominator is the number of patients who received treatment in each trial arm).

In the 41 patients receiving paclitaxel plus cediranib, adverse events leading to dose reduction or treatment interruption of cediranib occurred in 22% (n = 9) and 37% (n = 15), respectively. In the 41 patients receiving olaparib plus cediranib, adverse events leading to dose reduction of cediranib or olaparib occurred in 5% (n = 2) and 15% (n = 6), and treatment interruption occurred in 29% (n = 12) and 17% (n = 7), respectively. Five patients discontinued treatment because of adverse events (four in arm 2 and one in arm 3). No deaths were considered related to trial treatment. Detailed information on treatment duration, relative dose intensity, total dose delivered, dose intensity, AEs, SAEs, and SUSARs are included in the Supplementary Materials.

Patient-reported outcome data were provided by 123 patients (41 per arm). For the EORTC QLQ-C30 and EN24 questionnaires, the proportion of missing data at each timepoint (including withdrawals and deaths) was balanced across trial arms and remained around 5% between cycles 1 and 6, increasing to approximately 20% by cycle 10. Quality-of-life scores significantly improved for the role (p = 0.003) and social (p < 0.001) functioning scales, and for constipation (p = 0.022), lymphoedema (p = 0.022), and urological (p = 0.007) symptom scales in arm 2 compared with arm 1, but worsened for diarrhoea (p < 0.001) and gastrointestinal (p < 0.001) symptom scales (Appendix pp 27 and 28).

Significantly improved quality-of-life scores were reported in arm 3 compared with arm 1 for the global health (p < 0.001), physical (p < 0.001), role (p < 0.001), and social (p < 0.001) functioning scales, and for fatigue (p < 0.001), pain (p < 0.001), dyspnoea (p = 0.027), appetite loss (p < 0.001), urological (p < 0.001), body image (p = 0.023), back/pelvic pain (p = 0.003), tingling/numbness (p = 0.003), muscular/joint pain (p < 0.001), and taste change (p < 0.001) symptom scales. However, scores worsened for emotional (p < 0.001) and cognitive (p = 0.005) functioning scales, and for nausea and vomiting (p < 0.001), diarrhoea (p < 0.001), and gastrointestinal (p = 0.015) symptom scales (Appendix pp 27 and 28).

Archival tumour tissue was available from 105 patients for molecular subtyping. Of these, 15 tissue blocks could not be classified because they contained no tumour (n = 12) or immunohistochemistry failed (n = 3). Among the remaining 90 blocks, 18 (20%), 49 (54%), and 23 (26%) tumours were classified as dMMR, p53abn, and NSMP, respectively (Appendix p 29). dMMR was more frequent in endometrioid tumours (p < 0.001), and p53abn was more frequent in serous tumours (p < 0.001). Exploratory analysis showed no interaction between molecular subgroup and trial arm, although dMMR tumours were slightly more common in arm 2 (n = 9) than in arm 1 (n = 5) or arm 3 (n = 4). Univariable analysis showed that higher tumour grade and dMMR status were associated with worse PFS at 3 months (Appendix p 30). Multivariable logistic regression showed significantly better PFS at 3 months in arm 2 versus arm 1 (p = 0.011), consistent with the primary analysis, and significantly worse PFS at 3 months for patients with dMMR tumours compared with p53abn tumours across all arms (p = 0.006; Appendix p 31).

**Fig. 4:**
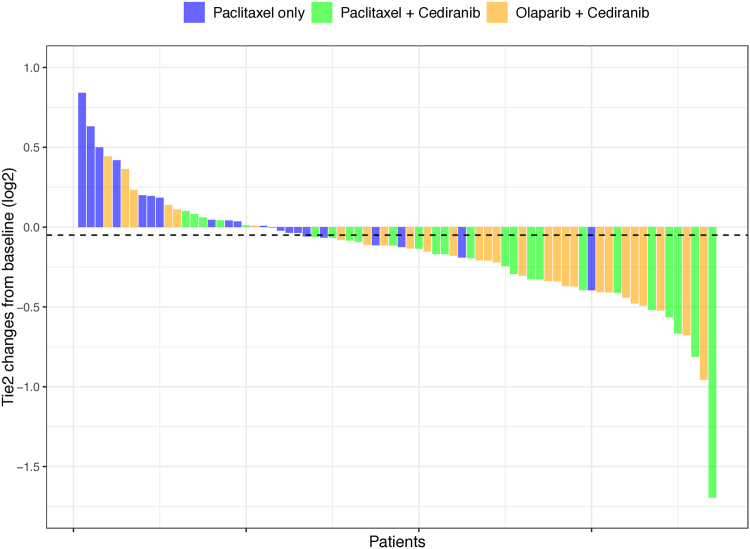
**Waterfall plot showing change in plasma Tie2 (log2) from pre-treatment value to day 1 cycle 3**. Patients receiving paclitaxel had an average Tie2 increase of 7%. The dashed black line represents a 5% or more reduction in plasma Tie2, which defined vascular response.

Pre-treatment Tie2 values were available for 106 patients (33 in arm 1, 34 in arm 2, and 39 in arm 3; Appendix p 32). The average pre-treatment plasma Tie2 value did not differ significantly between arms (Appendix p 32). Of the 106 evaluable patients, those receiving paclitaxel alone (n = 33) showed a modest average increase in plasma Tie2 of 7%, whereas 42 of 73 patients (58%) treated with cediranib achieved a Tie2-defined vascular response (≥5% reduction; Fig. 4). The proportion of vascular responders in arm 2 (n = 20) and arm 3 (n = 22) did not differ significantly (Appendix p 33). There were significant differences in PFS (log-rank p < 0.001) and OS (log-rank p = 0.002) among patients treated with paclitaxel alone, cediranib-treated vascular responders, and cediranib-treated non-responders (Appendix pp 34 and 35). Multivariable regression analysis showed that cediranib-treated vascular responders had significantly better PFS (HR 0.54, 95% CI 0.33–0.88; p = 0.014) compared with patients treated with paclitaxel alone (Appendix pp 36 and 37). The greatest differences in HRs for PFS (HR 0.52, 95% CI 0.29–0.93; p = 0.026) and OS (HR 0.45, 95% CI 0.22–0.90; p = 0.023) between vascular responders and non-responders were observed in patients treated with olaparib plus cediranib (Appendix pp 38 and 39). There was no difference in the prevalence of grade 3 or higher adverse events between cediranib-treated vascular responders and non-responders (Appendix p 40).

CTC analysis was performed in 96 patients, and 29 had one or more CTCs detected at day 1 of cycle 1. CTCs were detected in patients with endometrial carcinoma (n = 24) and uterine carcinosarcoma (n = 5; Appendix p 41). Patients with detectable CTCs had significantly shorter median PFS (5.0 versus 7.2 months; p = 0.0014) and median OS (11.2 versus 14.5 months; p = 0.017) than those with no detectable CTCs (Appendix pp 42 and 43). Multivariable regression showed that pre-treatment detectable CTCs were associated with worse PFS (HR 2.13; p = 0.004) and OS (HR 1.75; p = 0.036; Appendix p 44). These findings indicate that detectable CTCs were associated with poorer survival but did not predict treatment-specific response in COPELIA.

## Discussion

In this phase 2, hypothesis-generating trial, we compared a standard second-line therapy, paclitaxel, with two regimens targeting angiogenesis and DNA repair: paclitaxel plus cediranib and olaparib plus cediranib, in patients with recurrent endometrial cancer previously treated with platinum-based chemotherapy. Most patients enrolled had a poor prognosis and limited treatment options, with 79% having high-grade disease, 80% having an MMR-proficient tumour, and 81% having received two or more prior lines of chemotherapy. The primary endpoint was met, with a statistically significant improvement in PFS at 3 months in patients receiving paclitaxel plus cediranib compared with paclitaxel alone. The RECIST response rate was also significantly higher with paclitaxel–cediranib. However, no significant differences were observed between arms in PFS at 6 months, median PFS, or median OS. Notably, a higher proportion of patients treated with paclitaxel plus cediranib also experienced grade 3 treatment-related adverse events compared with those receiving paclitaxel. The combination of olaparib plus cediranib did not demonstrate improved efficacy compared with paclitaxel alone.

Three key considerations should inform the design of future trials evaluating paclitaxel plus cediranib in endometrial cancer. First, whether to include dMMR tumours. Endometrial cancers with dMMR generally derive the greatest benefit from immunotherapy, and multiple phase 3 trials have now demonstrated substantial survival improvements with anti-PD-1/PD-L1 agents in this subgroup.^31^, ^32^, ^33^, ^34^ When COPELIA was initiated, immunotherapy was not standard of care and therefore was not incorporated into the trial design.^23^^,^^24^ As a result, it remains unclear whether paclitaxel plus cediranib retains efficacy following prior immunotherapy. In COPELIA, dMMR tumours responded less well to paclitaxel, cediranib, and/or olaparib than p53abn tumours. A similar pattern was observed in the phase 2 GOG-086P trial, in which p53-overexpressing tumours derived the greatest benefit from chemotherapy plus bevacizumab.^35^ In contrast, in the control arm of the phase 3 RUBY trial, median PFS did not differ between dMMR and p53abn tumours.^31^ In COPELIA, the association between molecular subtype and vascular response was relatively weak (p = 0.724), suggesting that these factors may exert independent effects on patient outcomes. It is also notable that the number of patients within each molecular subtype was small, precluding formal assessment of subtype treatment interactions; larger studies will be required to determine whether cediranib benefit is enriched in specific subgroups such as p53abn/copy-number-high disease.

Second, future trials should consider whether paclitaxel plus cediranib should be continued beyond six cycles. In COPELIA, the PFS curves for paclitaxel plus cediranib and paclitaxel alone converged at six months, suggesting a loss of efficacy in the combination arm once treatment was stopped. All best radiological responses occurred within the first six months, and paclitaxel plus cediranib produced a significantly higher response rate than paclitaxel alone, supporting enhanced early activity of the combination. This likely contributed to the significant difference in 3-month PFS between arm 1 and arm 2, but not to differences at 6 months, nor to median PFS or OS. These findings indicate that any future trial should evaluate continuation of paclitaxel plus cediranib beyond six cycles, potentially to disease progression, to maximise therapeutic benefit.

Third, careful attention to patient selection, counselling, dose-modification strategies, and toxicity–management pathways will be essential in any future trial to minimise the risk of grade 3 adverse events associated with paclitaxel plus cediranib.

The enhanced efficacy observed in the paclitaxel plus cediranib arm is unlikely to reflect unexpectedly poor performance of the control arm. The response rate in arm 1 was 28%, consistent with the 18% and 27% response rates reported in two phase 2 trials of weekly paclitaxel monotherapy (80 mg/m^2^ on days 1, 8, and 15 of a 28-day cycle) in endometrial cancer.^25^^,^^36^

No new safety signals were identified with paclitaxel, cediranib, or olaparib. As expected, alopecia and neuropathy were more frequent in paclitaxel-treated patients, diarrhoea and mucositis in those receiving cediranib, and nausea and vomiting in those treated with olaparib. The rate of grade 3 hypertension in arm 3 (2%) was substantially lower than the 33% reported in the cediranib-only arm of the phase 2 NRG-GY012 trial evaluating olaparib plus cediranib versus monotherapy.^37^ Negative patient-reported outcomes in the paclitaxel plus cediranib arm were consistent with treatment-related adverse events. Despite this, dose intensity for both paclitaxel and cediranib in arm 2 exceeded 93%, indicating that the regimen was generally tolerable.

Our research group has now demonstrated the use of plasma Tie2 as a vascular response biomarker in four tumours, using two classes of VEGF inhibitor.^26^, ^27^, ^28^, ^29^ Here, we showed that cediranib-treated vascular responders had significantly better median PFS than vascular non-responders. These findings align with our previous data, where the HR for PFS benefit in vascular responders across all tumours was 0.55–0.70.^26^, ^27^, ^28^, ^29^

The most profound difference in HRs between cediranib-treated vascular responders and non-responders was seen in patients receiving olaparib plus cediranib. Indeed, assessing vascular response within individual treatment arms showed that olaparib plus cediranib conferred additive benefit, with vascular responders experiencing improved PFS and OS. The implication being those non-responders in arm 3 effectively received olaparib only, which has limited efficacy in endometrial cancer.^37^ This problem was exacerbated by toxicity from cediranib, which occurred at the same rate in vascular responders and non-responders. Conversely, in arm 2, patients whose tumours did not respond to cediranib still received paclitaxel, which is efficacious in endometrial cancer.^25^^,^^36^ Given that the clinical value of Tie2-defined vascular response has now been demonstrated in four tumour types, using bevacizumab or cediranib, this biomarker should be considered in future trials of VEGF inhibitors.

There are several limitations to COPELIA. First, stratification did not include molecular subtype, and only 73% of tumours in the primary analysis set underwent molecular evaluation. We recognise that the slightly higher prevalence of p53abn tumours in arm 2 may have influenced the primary endpoint. However, molecular subtyping was not standard practice when the trial was designed, and incorporation of these factors into the stratification scheme was therefore not feasible. Second, *POLE* testing was not available, so the frequency of *POLE*-mutant tumours in the cohort is unknown. The likelihood of recurrent endometrial cancer arising from a *POLE*-mutant primary tumour is low, suggesting that only a small number of cases, if any, would have been missed.^34^^,^^38^ Third, the Tie2 analysis is exploratory, may be subject to post-hoc bias, and is limited by the modest sample size. Fourth, the limited efficacy of olaparib plus cediranib should be interpreted in the context of absent predictive biomarker data, particularly *BRCA1/2* mutation status, homologous recombination deficiency, and PTEN loss.^21^^,^^22^ Fifth, because no patients had received prior immunotherapy, the benefit of paclitaxel plus cediranib in the post-immunotherapy setting remains unknown.^31^, ^32^, ^33^, ^34^ Finally, no data on race or ethnicity were collected. Despite these limitations, COPELIA has several strengths: all protocol-specified criteria for the primary analysis were met; a broad range of secondary endpoints, including quality-of-life outcomes, were comprehensively evaluated; median follow-up was robust; and all post-hoc and translational analyses were undertaken using rigorous multivariable methods.

In conclusion, COPELIA showed that paclitaxel plus cediranib improved PFS at 3 months and RECIST response compared with paclitaxel alone. Changes in plasma Tie2 during the first 9 weeks of cediranib also identified a subgroup of patients more likely to benefit. The next step will be to design a clinical trial evaluating paclitaxel plus cediranib in patients with MMR-proficient tumours who have received prior platinum chemotherapy and immunotherapy, a population with limited effective treatment options. Such a study should incorporate plasma Tie2 as a response biomarker to guide treatment continuation.

## Contributors

Conceptualisation: GCJ, ARC. Data curation: RDM, CP, CZ, ACC, GCJ, ARC. Formal analysis: RDM, CP, CZ, ACC, GCJ, ARC. Funding acquisition: RK, GCJ, ARC. Investigation: RDM, RK, SD, IV, AG, GE, AxW, AF, AHS, AnH, LH, ReR, RL, RB, JW, CS, PJM, GCJ, ARC. Methodology: RDM, CP, CZ, ACC, GCJ, ARC. Project administration: AnW, ACC, AH, JBS, LSN, RA, RuR, EB, WEP, JC, TMC, MN, MZ, MC. Resources: CD, GCJ, ARC. Software: CP, CZ, ACC. Supervision: GCJ, ARC. Validation: RDM, CP, CZ, SD, HL, LE, KM, MGD, AFG, DM, DB, PJM, JT, PJM, RJE, CD, GCJ, ARC. Visualisation: RDM, CP, CZ, GCJ, ARC. Assessed and verified the underlying data: GCJ, ARC. Writing—original draft: RDM, CP, CZ, ACC, GCJ, ARC. Writing—review & editing: All authors.

## Data sharing statement

All data supporting this study are provided in the manuscript and supplementary appendix. Access to additional de-identified data is available through formal application to the Centre for Trials Research at Cardiff University.

## Declaration of interests

RDM, CP, CZ, SD, IV, AG, AxW, AF, AHS, AnH, ReR, JW, CS, AnW, AH, JBS, LSN, RuR, EB, WEP, JC, TMC, MN, HL, LE, MZ, KM, MD-G, AFG, DB, MC, PJM and CD declare no competing interests. RK declares grants from Guy's Cancer Charity, consulting fees from Pharma&, Clovis Oncology, AstraZeneca, GSK, MSD, honoraria from Pharma&, AstraZeneca, GSK, MSD, support for attending meetings from Pharma&, GSK, and participation on Advisory Boards for Pharma&, AstraZeneca, GSK, MSD. GE declares consultancy fees from Eisai and MSD, honoraria from Eisai, MSD and GSK, travel support for attending meetings from MSD, and participation on Data Safety Monitoring/Advisory Boards for Eisai, MSD, GSK, Genmab and Regeneron. LH declares royalties from Cambridge University Press, support for attending meetings/travel from The Chinese University of Hong Kong, Roche, MIMS, Tata Medical Centre and The Royal College of Radiologists, leadership roles for The Royal College of Radiologist and Wales Cancer Team and financial interests from practicing privileges with AXA, Aviva and Sciensus. RL declares herself as the Chief Investigator for an investigator-initiated study in cervical cancer using cediranib and olaparib, funded by AstraZeneca, and honoraria and advisory board fees from GSK. RB declares consulting fees from Novartis and Genmab, honoraria from Novartis, GSK, Pharma&, AstraZeneca, Daiichi Sankyo, AbbVie and Pfizer, and support for attending meetings and/or travel from Daiichi Sankyo, AbbVie, Astra Zeneca and Pharma&. ACC declares institutional educational grants from AstraZeneca.

RA declares consulting fees from Bayer, BMS, Takeda, Servier, Nordic Pharma and Artios Pharma, honoraria from Pierre Fabre, Servier and Takeda, and support for attending meetings and/or travel from Servier and Takeda. DM declares a financial interest in AstraZeneca, holding shares purchased through the employee share retention scheme during prior employment between 2007 and 2017. JT declares a financial interest in AstraZeneca, holding shares purchased. RJE declares consulting free from GSK not related to this study, honoraria from AstraZeneca not related to this study, and declares himself the Chair of the PROTECTOR Study Data Safety Monitoring Board GCJ declares grants and consulting fees from AstraZeneca and a financial interest in Sanofi, holding shares purchased. ARC declares grants from AstraZeneca and honoraria from GSK.

## Appendix A Supplementary data

Supplementary Appendix

Trial Protocol
