# Supplementary material for: Cediranib with weekly paclitaxel or olaparib versus weekly paclitaxel for advanced or recurrent endometrial cancer (COPELIA): a multicentre, open-label, randomised, phase 2 trial in the UK
Source: eClinicalMedicine. 2026 Jun 16;96:104012. doi: 10.1016/j.eclinm.2026.104012 (PMC13284432; doi:10.1016/j.eclinm.2026.104012)
Supplement: Supplementary Appendix [file mmc1.docx]

**Cediranib with weekly paclitaxel or olaparib versus weekly paclitaxel for advanced or recurrent endometrial cancer (COPELIA): a multicentre, open-label, randomised, phase 2 trial**

**Supplementary Appendix**

**Table of Contents**

[Trial centres 3](#_Toc226658267)

[Eligibility criteria 4](#_Toc226658268)

[Dose levels 6](#_Toc226658269)

[Dose modification 7](#_Toc226658270)

[Treatment discontinuation 7](#_Toc226658271)

[Immunohistochemistry 8](#_Toc226658272)

[Plasma Tie2 9](#_Toc226658273)

[Circulating Tumour Cells 11](#_Toc226658274)

[Supplementary Table S1: Treatment duration and dose intensity 13](#_Toc226658275)

[Supplementary Table S2: Adverse events 14](#_Toc226658276)

[Supplementary Figure S1: Adverse events of any grade experienced in ≥10% of patients. 21](#_Toc226658277)

[Supplementary Figure S2: Adverse events of any grade experienced in <10% of patients. 22](#_Toc226658278)

[Supplementary Table S3: Frequency of Serious Adverse Events and Reactions 23](#_Toc226658279)

[Supplementary Table S4: Serious Adverse Events 24](#_Toc226658280)

[Supplementary Table S5: Serious Adverse Reactions 25](#_Toc226658281)

[Supplementary Table S6: Suspected Unexpected Serious Adverse Reactions 26](#_Toc226658282)

[Supplementary Table S7: Health-related quality-of-life data 27](#_Toc226658283)

[Supplementary Table S8: Molecular subtyping 29](#_Toc226658284)

[Supplementary Table S9: Association between clinical variables and PFS at 3 months 30](#_Toc226658285)

[Supplementary Table S10: Multivariable logistic regression analysis of PFS at 3 months 31](#_Toc226658286)

[Supplementary Table S11: Average pre-treatment plasma Tie2 value in each trial arm. 32](#_Toc226658287)

[Supplementary Table S12: Vascular response 33](#_Toc226658288)

[Supplementary Figure S3: Kaplan-Meier curves for PFS of cediranib-treated vascular response 34](#_Toc226658289)

[Supplementary Figure S4: Kaplan-Meier curves for OS of cediranib-treated vascular response 35](#_Toc226658290)

[Supplementary Table S13: Univariable Cox proportional hazards analysis for clinical variables at baseline 36](#_Toc226658291)

[Supplementary Table S14: Multivariable Cox proportional hazards analysis for vascular response 37](#_Toc226658292)

[Supplementary Figure S5: Additive PFS benefit in vascular responders receiving olaparib 38](#_Toc226658293)

[Supplementary Figure S6: Additive OS benefit in vascular responders receiving olaparib 39](#_Toc226658294)

[Supplementary Table S15: Adverse events in vascular responders and non-responder 40](#_Toc226658295)

[Supplementary Table S16: Circulating tumour cell data. 41](#_Toc226658296)

[Supplementary Figure S7. Kaplan-Meier curves for progression-free survival according to CTC status 42](#_Toc226658297)

[Supplementary Figure S8. Kaplan-Meier curves for overall survival according to CTC status 43](#_Toc226658298)

[Supplementary Table S17. Multivariable Cox proportional hazards analysis for circulating tumour cells 44](#_Toc226658299)

## Trial centres

| **Country** | **Site Name** | **Principal Investigator** | **Patients randomised** |
| --- | --- | --- | --- |
| England | The Christie NHS Foundation Trust | Professor Gordon Jayson | 27 |
| England | East and North Hertfordshire NHS Trust | Dr Ignacio Vazquez | 16 |
| England | The Royal Marsden NHS Foundation Trust | Dr Angela George | 12 |
| England | University College London Hospitals NHS Foundation Trust | Dr Gemma Eminowicz | 12 |
| England | University Hospitals Bristol NHS Foundation Trust | Dr Axel Walther | 11 |
| England | Royal Surrey County Hospital NHS Foundation Trust | Dr Adrian Franklin | 10 |
| Scotland | The Beatson West of Scotland Cancer Centre | Dr Azmat Sadozye | 8 |
| England | Newcastle Hospitals NHS Foundation Trust | Dr Andrew Hughes | 6 |
| Wales | Velindre University NHS Trust | Dr Louise Hanna | 6 |
| England | Oxford University Hospitals NHS Foundation Trust | Dr Rene Roux | 5 |
| England | Wirral University Teaching Hospital NHS Foundation Trust | Dr Rosemary Lord | 4 |
| England | Royal United Hospital Bath NHS Foundation Trust | Dr Rebecca Bowen | 3 |
| England | Guy’s and St Thomas’ NHS Foundation Trust | Dr Rebecca Kristeleit | 2 |
| England | University Hospitals of Leicester NHS Trust | Dr Joey Wood | 2 |

## Eligibility criteria

**Inclusion criteria**

1. Age 16 years or older
2. Histologically confirmed endometrial carcinoma or carcinosarcoma
3. Eastern Cooperative Oncology Group performance status of 0 or 1
4. Response Evaluation Criteria in Solid Tumours (RECIST) version 1·1 measurable disease
5. Haemoglobin ≥90 grams per L, platelet count ≥100 × 10^9^ per L and absolute neutrophil count ≥1·5 × 10^9^ per L
6. International normalised ratio <1.4 and activated partial thromboplastin ratio <1·4
7. Bilirubin ≤1·5 × upper limit of normal (ULN), aspartate aminotransferase and alanine transaminase ≤2·5 × ULN in the absence of liver metastasis (<5·0 × ULN in the presence of liver metastases)
8. Creatinine clearance (Wright or Cockcroft-Gault formula) ≥51 mL per minute or a measured radioisotopic glomerular filtration rate ≥51 mL per minute
9. Negative or trace proteinuria on dipstick urinalysis
10. Adequately controlled thyroid function
11. Life expectancy of ≥16 weeks.

**Exclusion criteria**

1. Prior treatment with dose-dense weekly paclitaxel.
2. Prior or concurrent therapy with a PARP or VEGF inhibitor.
3. Uncontrolled brain metastases or seizures.
4. Known positivity for hepatitis B, hepatitis C or HIV.
5. Resting ECG with QTc > 470 millisecond on ≥2 time points within a 24-hour period or family history of long QT syndrome.
6. Concomitant use of known strong or moderate CYP3A inhibitors.
7. Concomitant use of known strong or moderate CYP3A inducers.
8. Pregnant or lactating.
9. Of child bearing potential and not willing to ensure use effective contraception throughout the treatment period and for six months following the end of treatment.
10. Side effects of previous treatments have not resolved to grade 1 or less, except for alopecia that is considered related to cytotoxic chemotherapy.
11. Radiotherapy, chemotherapy, surgery or tumour embolisation within 28 days before the first dose of Investigational Medicinal Product.
12. Additional concurrent anti-cancer therapy.

Causes of malabsorption, e.g. uncontrolled diarrhoea or poorly controlled stoma.

1. Bowel obstruction, fistulae, impending fistulation seen on radiological imaging, or extensive rectosigmoid involvement by cancer.
2. Inadequately controlled hypertension, defined as ≥150/90 mmHg.
3. Known hypersensitivity to olaparib, cediranib or any of the excipients of the products.
4. Known hypersensitivity to paclitaxel that in the opinion of the investigator would prevent administration of a weekly paclitaxel regimen.
5. Exposure to an investigational agent within 30 days or 5 half-lives (whichever is the longer) prior to enrolment.
6. Considered a poor medical risk due to a serious, uncontrolled medical disorder, non-malignant systemic disease or active, uncontrolled infection.
7. Myelodysplastic syndrome (MDS), acute myeloid leukaemia (AML) or features suggestive of MDS/AML.
8. Other malignancy within the last 5 years except: adequately treated non-melanoma skin cancer, curatively treated in situ cancer of the cervix, ductal carcinoma in situ, or other solid tumours including lymphomas (without bone marrow involvement) curatively treated with no evidence disease for ≥5 years.
9. Prior allogeneic bone marrow transplant or double umbilical cord blood transplantation.

## Dose levels

| **Drug** | **Dose Level** | **Dose** |
| --- | --- | --- |
| Paclitaxel | Starting dose | 80 mg/m^2^ by body surface area |
|  | Minus 1 | 65 mg/m^2^ by body surface area |
|  | Minus 2 | 50 mg/m^2^ by body surface area |
| Cediranib (Investigational Medicinal Product) | Starting dose | 20 mg once daily |
|  | Minus 1 | 15 mg once daily |
| Olaparib (Investigational Medicinal Product) | Starting dose | 300 mg twice daily |
|  | Minus 1 | 250 mg twice daily |
|  | Minus 2 | 200 mg twice daily |

## Dose modification

Paclitaxel was interrupted on day 1 for an absolute neutrophil count of <1·5 × 10^9^ per L, a platelet count <100 × 10^9^ per L or a bilirubin >1·5 × upper limit of normal (ULN) or an AST or ALT >2·5 × ULN (or an AST or ALT >5·0 × ULN in the presence of liver metastases). Paclitaxel was omitted on days 8 or 15 for an absolute neutrophil count of <1·0 × 10^9^ per L or a platelet count <75 × 10^9^ per L. Cediranib was interrupted for grade ≥2 proteinuria (≥1·0 grams per 24 hours) in the absence of any urinary tract infection, grade ≥3 pulmonary embolism, grade 2–3 diarrhoea or grade ≥2 fatigue. Olaparib was interrupted for grade ≥3 anaemia, an absolute neutrophil count <1·0 × 10^9^ per L, a platelet count <50 × 10^9^ per L or grade ≥3 febrile neutropenia. Paclitaxel, cediranib or olaparib were interrupted for any grade ≥2 adverse event considered by the treating team to warrant interruption.

## Treatment discontinuation

Paclitaxel was permanently discontinued for grade ≥3 peripheral neuropathy, any grade of pneumonitis (clinical symptoms plus radiological evidence on computed tomography) or grade 3–4 hypersensitivity reaction. Cediranib was permanently discontinued for grade 4 diarrhoea, grade 4 hypertension despite maximal anti-hypertensive treatment, grade 4 venous thromboembolism, grade 3–4 haemorrhage, any grade of fistula or gastrointestinal perforation, any grade of arterial thromboembolism or reversible posterior leukoencephalopathy syndrome (RPLS). Olaparib was permanently discontinued for MDS/AML or any other clonal blood disorder or any grade of pneumonitis. Paclitaxel, cediranib or olaparib were permanently discontinued for any grade 4 adverse event considered by the treating team to warrant discontinuation.

## Immunohistochemistry

Immunohistochemistry was performed at the Cancer Research UK National Biomarker Centre (University of Manchester, England; GCLP-linked laboratory). Archival FFPE tissue was requested for all patients who optionally consented to donate tissue. Five 4mm thick sections were cut and stained using the antibodies listed in the table below. Chromogenic immunohistochemistry assays were performed on a BOND RX Platform with Bond Polymer Refine Detection Kit (DS9800) (Leica Biosystems) or a Benchmark ULTRA System with OptiView DAB IHC Detection Kit (750-700) and OptiView Amplification Kit (760-099) (Roche Diagnostics). Positive controls were benign tonsil tissue.

Immunohistochemistry (IHC) was assessed by a Consultant Histopathologist with a specialist interest in Gynaecological Pathology, who was blinded to all clinical, treatment, and outcome data, and scored all stained slides on low (x10) and high (x40) power objectives for each protein marker. Tumours with loss of expression of one or more mismatch repair (MMR) proteins (MLH1, MSH2, MSH6, PMS2) were classified as MMR-deficient (dMMR) regardless of p53 expression. Tumours with normal expression of all MMR proteins and mutant p53 expression were classified as p53 abnormal (p53abn). Tumours were only molecularly classified if IHC was successful for all MMR proteins and p53 protein.

| **Primary Antibody** | **Company/Cat No** | **Clone** | **Dilution** | **Antigen retrieval** | **Antibody incubation** |
| --- | --- | --- | --- | --- | --- |
| p53 | Leica Biosystems/PA0057 | D0-7 | Ready to use | pH 9, 20 mins | 36ºC, 20 mins |
| MSH2 | Roche Diagnostics/760-5093 | G219-1129 | Ready to use | CC1, 40 mins | 36ºC, 12 mins |
| MSH6 | Roche Diagnostics/760-5092 | SP93 | Ready to use | CC1, 64 mins | 36ºC, 12 mins |
| MLH1 | Roche Diagnostics/760-5091 | M1 | Ready to use | CC1, 64 mins | 36ºC, 24 mins |
| PMS2 | Roche Diagnostics/760-5094 | A16-4 | Ready to use | CC1, 92 mins | 36ºC, 32 mins |

## Plasma Tie2

Whole blood was tested for plasma Tie2 at the Cancer Research UK National Biomarker Centre (University of Manchester, Manchester, England). Whole blood was collected at the screening visit, and then on day 1 of cycle 1, 2 and 3, and then at the end of trial visit. 10 mL of whole blood was drawn into a EDTA collection tube. Plasma was separated from blood cells within 30 minutes of drawing by centrifugation at 1000g for 10 minutes. The plasma was transferred into a sterile 15ml Falcon tube before centrifuging again at 2000g for 10 minutes. Plasma supernatant aliquots were transferred into 1·8 mL cryovials without disturbing the cell pellet. If the red cell layer after the first centrifugation or the cell pellet after the second centrifugation was disturbed, the plasma sample was re-spun. All plasma samples were transferred immediately to a -80ºC freezer.

Analytes of interest were quantified using the Simoa® SP-X Planar Array Multiplex Sandwich ELISA Kits (Quanterix) as listed in the table below. Plasma was thawed, centrifuged at 2000g for 5 minutes and diluted as listed in the table below. After an initial wash of the plate (4 × cycles of 300 µL), 50 µL of plasma was added to wells pre-spotted with capture antibodies. The plate was then incubated for 120 minutes at room temperature on a plate shaker (400–600 RPM). A second wash was performed (4 × cycles of 300 µL), then 50 µL of Biotinylated Antibody Reagent was added to the wells for 30 minutes at room temperature on a plate shaker (400–600 RPM). A third wash was performed (4 × cycles of 300 µL) followed by addition of 50 µL of Streptavidin-HRP Reagent. Plates were incubated for a further 30 minutes at room temperature on a plate shaker (400–600 RPM). A final wash was performed (4 × cycles of 300 µL) followed by the addition of 50 µL SuperSignal® Substrate, which was briefly incubated in the ELISA plate before quantification using the SP-X Imaging and Analysis System (Quanterix). The lyophilized recombinant calibrators included in the kit were diluted as per the manufacturer’s instructions to create a standard curve for each analyte. Analyte concentrations (pg/mL) were determined using the Cirasoft Analyst Software. All calibrators and samples were run in duplicate, and the mean values were calculated.

| **Assay Name** | **Target Analytes** | **Sample Dilution** |
| --- | --- | --- |
| Human 2-Plex Kit  Product Number 100-0181 | Ang-1  Tie-2 | 1:10 (1 part sample to 9 parts sample diluent) |

## Circulating Tumour Cells

Whole blood was tested for circulating tumour cells (CTCs) at UCL Cancer Institute (University College London, England; GCLP-linked laboratory). Whole blood was collected for CTCs on day 1 cycle 1. 10 mL  of whole blood was drawn into a CellSave® preservative tube (Menarini Silicon Biosystems). Samples were maintained at ambient temperature and processed for CTCs within 96 hours of collection. Samples were mixed thoroughly by inverting eight times and 7·5 mL transferred to a conical centrifuge tube and mixed with 6·5 mL of dilution buffer (CELLSEARCH® CTC kit components). The sample and buffer were centrifuged at 800g for 10 minutes. Following centrifugation, samples were loaded onto the CELLTRACKS® AUTOPREP® System for automated processing using the CELLSEARCH® CTC kit (Menarini Silicon Biosystems). In every batch analysed, a CELLSEARCH® CTC control sample (Menarini Silicon Biosystems) was run alongside trial samples to confirm proper system functioning.

A ferrofluid reagent consisting of magnetic particles coated with anti-EpCAM antibodies was used for capture and enrichment of CTCs from blood, before fluorescent reagents (anti-CK-PE [specific for the intracellular protein cytokeratin cells], DAPI, and anti-CD45-APC [specific for leukocytes]) were added for identification and analysis of CTCs. The processed sample was then dispensed into a barcode labelled cartridge contained within a MAGNEST® Cartridge Holder for image acquisition using the CELLTRACKS ANALYZER II®. Cartridges were scanned using the CELLTRACKS ANALYZER II® between 20 minutes and 24 hours after preparation by the CELLTRACKS® AUTOPREP® System. The CELLTRACKS ANALYZER II® scanned the entire surface of the cartridge in each fluorescent channel (PE, DAPI, APC). The software then displayed events to the analyst where CK-PE and DAPI fluorescent signal were co-located for classification. All samples were reviewed by two analysts to identify CTCs according to CELLSEARCH standardised interpretation criteria. Events are classified as CTCs when they have the morphology of an intact cell and are CK-PE positive, DAPI positive, and CD45-APC negative. Results were reported as the number of CTCs per 7·5 mL whole blood.

## Supplementary Table S1: Treatment duration and dose intensity

Key: IQR, interquartile range; * Paclitaxel was given on days 1, 8, 15 of every 28-day cycle and so expected weekly dose intensity for a complete cycle is 60 mg/m^2^/week, not 80 mg/m^2^/week.

|  | **Arm 1**  Paclitaxel | **Arm 2**  Paclitaxel/cediranib | **Arm 3**  Olaparib/cediranib |
| --- | --- | --- | --- |
| **Treatment duration – median (IQR)** |  |  |  |
| Paclitaxel (weeks) | 16·0 (8·0–24·0) | 23·9 (16·0–24·0) | - |
| Cediranib (weeks) | - | 23·5 (14·3–39·7) | 15·0 (9·4–43·9) |
| Olaparib (weeks) | - | - | 15·0 (9·4–43·9) |
| **Relative dose intensity – median (IQR)** |  |  |  |
| Paclitaxel (%) | 100 (88·2–100) | 95·2 (85·7–100) | - |
| Cediranib (%) | - | 93·4 (80·6–100) | 99·7 (93·6–100) |
| Olaparib (%) | - | - | 97·5 (83·2–100) |
| **Total dose delivered – median (IQR)** |  |  |  |
| Paclitaxel (mg) | 1,323 (763–2,395) | 1,984 (1,564–2,410) | - |
| Cediranib (mg) | - | 2,800 (1,680–5,460) | 1,990 (1,120–5,330) |
| Olaparib (mg) | - | - | 55,875 (30,800–151,200) |
| **Dose intensity – median (IQR)** |  |  |  |
| Paclitaxel (mg/m^2^/week) * | 60.0 (52·9–60·0) | 57·1 (51·4–60·0) | - |
| Cediranib (mg/day) | - | 18.7 (16·1–20·0) | 19·9 (18·7–20·0) |
| Olaparib (mg/day) | - | - | 585 (499–600) |

## Supplementary Table S2: Adverse events

| **CTCAE** | **Arm 1**  Paclitaxel | | | **Arm 2**  Paclitaxel/cediranib | | | | **Arm 3**  Olaparib/cediranib | | | |
| --- | --- | --- | --- | --- | --- | --- | --- | --- | --- | --- | --- |
|  | **Grade 1/2** | **Grade 3** | **Grade 4** | **Grade 1/2** | **Grade 3** | **Grade 4** | **Grade 1/2** | | **Grade 3** | **Grade 4** |  |
| Abdominal cavity drainage | 0 | 0 | 0 | 0 | 0 | 0 | 0 | | 1 | 0 |  |
| Abdominal discomfort | 0 | 0 | 0 | 0 | 0 | 0 | 1 | | 0 | 0 |  |
| Abdominal distension | 4 | 0 | 0 | 2 | 0 | 0 | 4 | | 0 | 0 |  |
| Abdominal pain | 10 | 0 | 0 | 18 | 3 | 0 | 18 | | 1 | 0 |  |
| Abdominal pain - lower | 0 | 0 | 0 | 1 | 0 | 0 | 0 | | 0 | 0 |  |
| Abdominal pain - upper | 0 | 0 | 0 | 3 | 0 | 0 | 4 | | 0 | 0 |  |
| Acne | 0 | 0 | 0 | 1 | 0 | 0 | 0 | | 0 | 0 |  |
| Acute kidney injury | 0 | 0 | 0 | 0 | 1 | 0 | 0 | | 0 | 0 |  |
| Agitation | 0 | 0 | 0 | 0 | 0 | 0 | 0 | | 0 | 1 |  |
| Allergic reaction | 2 | 0 | 0 | 0 | 0 | 0 | 0 | | 0 | 0 |  |
| Alopecia | 14 | 0 | 0 | 14 | 1 | 0 | 2 | | 0 | 0 |  |
| ALP increased | 2 | 0 | 0 | 6 | 0 | 0 | 2 | | 0 | 0 |  |
| ALT increased | 1 | 0 | 0 | 3 | 0 | 0 | 1 | | 1 | 0 |  |
| Anaemia | 16 | 1 | 0 | 9 | 1 | 0 | 10 | | 3 | 0 |  |
| Anal fissure | 0 | 0 | 0 | 1 | 0 | 0 | 0 | | 0 | 0 |  |
| Angular cheilitis | 1 | 0 | 0 | 0 | 0 | 0 | 0 | | 0 | 0 |  |
| Anorexia | 13 | 0 | 0 | 14 | 0 | 0 | 20 | | 2 | 0 |  |
| Anosmia | 0 | 0 | 0 | 1 | 0 | 0 | 0 | | 0 | 0 |  |
| Anxiety | 2 | 0 | 0 | 2 | 0 | 0 | 2 | | 0 | 0 |  |
| Aphthous ulcer | 0 | 0 | 0 | 1 | 0 | 0 | 0 | | 0 | 0 |  |
| Arthralgia | 6 | 0 | 0 | 6 | 0 | 0 | 3 | | 1 | 0 |  |
| Ascites | 0 | 0 | 0 | 1 | 0 | 0 | 1 | | 1 | 0 |  |
| Aspiration | 0 | 0 | 0 | 0 | 0 | 0 | 1 | | 0 | 0 |  |
| AST increased | 2 | 0 | 0 | 2 | 0 | 0 | 2 | | 1 | 0 |  |
| Asthma | 1 | 0 | 0 | 0 | 0 | 0 | 0 | | 0 | 0 |  |
| Back pain | 7 | 0 | 0 | 6 | 0 | 0 | 11 | | 1 | 0 |  |
| Blood albumin decreased | 0 | 0 | 0 | 0 | 0 | 0 | 1 | | 0 | 0 |  |
| Blood bilirubin increased | 1 | 0 | 0 | 0 | 0 | 0 | 0 | | 0 | 0 |  |
| Blood LDH increased | 0 | 0 | 0 | 1 | 0 | 0 | 0 | | 0 | 0 |  |
| Blood TSH decreased | 0 | 0 | 0 | 0 | 0 | 0 | 1 | | 0 | 0 |  |
| Blood TSH increased | 1 | 0 | 0 | 2 | 0 | 0 | 1 | | 0 | 0 |  |
| Body tinea | 0 | 0 | 0 | 0 | 0 | 0 | 1 | | 0 | 0 |  |
| Bone pain | 1 | 0 | 0 | 1 | 0 | 0 | 1 | | 0 | 0 |  |
| Burning sensation | 0 | 0 | 0 | 1 | 0 | 0 | 0 | | 0 | 0 |  |
| Cerebrovascular accident | 0 | 0 | 0 | 0 | 0 | 0 | 0 | | 1 | 0 |  |
| Chest pain | 1 | 0 | 0 | 0 | 0 | 0 | 0 | | 0 | 0 |  |
| CKD-mineral bone disorder | 0 | 0 | 0 | 0 | 0 | 0 | 1 | | 0 | 0 |  |
| Confusional state | 1 | 0 | 0 | 0 | 0 | 0 | 0 | | 1 | 0 |  |
| Conjunctivitis | 0 | 0 | 0 | 2 | 0 | 0 | 0 | | 0 | 0 |  |
| Constipation | 12 | 0 | 0 | 18 | 0 | 0 | 23 | | 0 | 0 |  |
| Cough | 5 | 1 | 0 | 8 | 0 | 0 | 9 | | 0 | 0 |  |
| COVID-19 | 1 | 0 | 0 | 0 | 0 | 0 | 2 | | 0 | 0 |  |
| Creatinine increased | 1 | 0 | 0 | 6 | 0 | 0 | 7 | | 0 | 0 |  |
| Dehydration | 0 | 0 | 0 | 0 | 1 | 0 | 0 | | 1 | 0 |  |
| Low mood | 0 | 0 | 0 | 1 | 0 | 0 | 0 | | 0 | 0 |  |
| Depression | 0 | 0 | 0 | 0 | 0 | 0 | 1 | | 0 | 0 |  |
| Dermatitis acneiform | 0 | 0 | 0 | 1 | 0 | 0 | 0 | | 0 | 0 |  |
| Dermatitis atopic | 1 | 0 | 0 | 0 | 0 | 0 | 0 | | 0 | 0 |  |
| Device related infection | 1 | 1 | 0 | 0 | 0 | 0 | 0 | | 0 | 0 |  |
| Diarrhoea | 10 | 0 | 0 | 30 | 4 | 0 | 30 | | 0 | 0 |  |
| Discomfort | 0 | 0 | 0 | 1 | 0 | 0 | 0 | | 0 | 0 |  |
| Disturbance of attention | 0 | 0 | 0 | 1 | 0 | 0 | 0 | | 0 | 0 |  |
| Diverticulitis | 1 | 0 | 0 | 0 | 0 | 0 | 0 | | 0 | 0 |  |
| Dizziness | 1 | 0 | 0 | 3 | 0 | 0 | 4 | | 0 | 0 |  |
| Dry Eye | 0 | 0 | 0 | 1 | 0 | 0 | 0 | | 0 | 0 |  |
| Dry mouth | 0 | 0 | 0 | 5 | 0 | 0 | 2 | | 0 | 0 |  |
| Dry skin | 1 | 0 | 0 | 5 | 0 | 0 | 1 | | 0 | 0 |  |
| Duodeno-gastric reflux | 0 | 0 | 0 | 1 | 0 | 0 | 0 | | 0 | 0 |  |
| Dysarthria | 1 | 0 | 0 | 0 | 0 | 0 | 0 | | 0 | 0 |  |
| Dysgeusia | 3 | 0 | 0 | 5 | 0 | 0 | 2 | | 0 | 0 |  |
| Dyspepsia | 3 | 0 | 0 | 8 | 0 | 0 | 10 | | 0 | 0 |  |
| Dysphagia | 1 | 0 | 0 | 0 | 0 | 0 | 0 | | 0 | 0 |  |
| Dyspnoea | 14 | 1 | 0 | 16 | 1 | 0 | 19 | | 0 | 0 |  |
| Dysuria | 0 | 0 | 0 | 1 | 0 | 0 | 0 | | 0 | 0 |  |
| Eczema | 0 | 0 | 0 | 1 | 0 | 0 | 0 | | 0 | 0 |  |
| Embolism | 0 | 0 | 0 | 0 | 0 | 0 | 1 | | 0 | 0 |  |
| Epistaxis | 2 | 0 | 0 | 4 | 0 | 0 | 0 | | 0 | 0 |  |
| Eye Infection | 0 | 0 | 0 | 1 | 0 | 0 | 0 | | 0 | 0 |  |
| Eye Pain | 0 | 0 | 0 | 1 | 0 | 0 | 0 | | 0 | 0 |  |
| Faecal incontinence | 0 | 0 | 0 | 1 | 0 | 0 | 0 | | 0 | 0 |  |
| Fall | 0 | 0 | 0 | 0 | 0 | 0 | 1 | | 2 | 0 |  |
| Fatigue | 25 | 2 | 0 | 29 | 3 | 0 | 27 | | 4 | 0 |  |
| Febrile neutropenia | 0 | 0 | 0 | 1 | 1 | 0 | 0 | | 0 | 0 |  |
| Flank pain | 1 | 0 | 0 | 4 | 0 | 0 | 0 | | 0 | 0 |  |
| Flatulence | 2 | 0 | 0 | 1 | 0 | 0 | 2 | | 0 | 0 |  |
| Free T3 decreased | 0 | 0 | 0 | 1 | 0 | 0 | 0 | | 0 | 0 |  |
| Fungal skin infection | 0 | 0 | 0 | 0 | 0 | 0 | 1 | | 0 | 0 |  |
| Furuncle | 1 | 0 | 0 | 2 | 0 | 0 | 0 | | 0 | 0 |  |
| Gait disturbance | 0 | 0 | 0 | 0 | 0 | 0 | 1 | | 0 | 0 |  |
| Gastroenteritis radiation | 1 | 0 | 0 | 0 | 0 | 0 | 0 | | 0 | 0 |  |
| Gastrointestinal perforation | 0 | 0 | 0 | 0 | 1 | 1 | 0 | | 1 | 0 |  |
| Gastro-oesophageal reflux disease | 2 | 0 | 0 | 3 | 0 | 0 | 2 | | 0 | 0 |  |
| GGT increased | 0 | 0 | 0 | 3 | 0 | 0 | 0 | | 0 | 0 |  |
| Glossodynia | 0 | 0 | 0 | 1 | 0 | 0 | 0 | | 0 | 0 |  |
| Glucose tolerance impaired | 1 | 0 | 0 | 0 | 0 | 0 | 0 | | 0 | 0 |  |
| Groin infection | 0 | 0 | 0 | 1 | 0 | 0 | 0 | | 0 | 0 |  |
| Groin pain | 1 | 0 | 0 | 0 | 0 | 0 | 0 | | 0 | 0 |  |
| Haematoma | 0 | 0 | 0 | 1 | 0 | 0 | 0 | | 0 | 0 |  |
| Haematuria | 1 | 0 | 0 | 0 | 0 | 0 | 0 | | 0 | 0 |  |
| Haemoptysis | 0 | 0 | 0 | 0 | 0 | 0 | 1 | | 0 | 0 |  |
| Haemorrhoids | 0 | 0 | 0 | 3 | 0 | 0 | 1 | | 0 | 0 |  |
| Headache | 4 | 0 | 0 | 9 | 1 | 0 | 2 | | 0 | 0 |  |
| Hepatic pain | 0 | 0 | 0 | 2 | 0 | 0 | 0 | | 0 | 0 |  |
| Hoarseness | 2 | 0 | 0 | 3 | 0 | 0 | 0 | | 0 | 0 |  |
| Hordeolum | 1 | 0 | 0 | 0 | 0 | 0 | 0 | | 0 | 0 |  |
| Hot flush | 1 | 0 | 0 | 1 | 0 | 0 | 0 | | 0 | 0 |  |
| Hypercalcaemia | 0 | 1 | 0 | 0 | 0 | 0 | 0 | | 0 | 0 |  |
| Hypercholesterolaemia | 1 | 0 | 0 | 0 | 0 | 0 | 0 | | 0 | 0 |  |
| Hyperglycaemia | 0 | 0 | 0 | 1 | 0 | 0 | 0 | | 0 | 0 |  |
| Hyperhidrosis | 0 | 0 | 0 | 1 | 0 | 0 | 0 | | 0 | 0 |  |
| Hyperkeratosis | 0 | 0 | 0 | 1 | 0 | 0 | 0 | | 0 | 0 |  |
| Hyperphosphatemia | 1 | 0 | 0 | 0 | 0 | 0 | 0 | | 0 | 0 |  |
| Hypertension | 5 | 0 | 0 | 13 | 6 | 0 | 9 | | 1 | 0 |  |
| Hyperthyroidism | 0 | 0 | 0 | 4 | 0 | 0 | 1 | | 0 | 0 |  |
| Hyperuricaemia | 1 | 0 | 0 | 0 | 0 | 0 | 0 | | 0 | 0 |  |
| Hypoaesthesia | 1 | 0 | 0 | 2 | 0 | 0 | 0 | | 0 | 0 |  |
| Hypoalbuminemia | 1 | 0 | 0 | 1 | 0 | 0 | 0 | | 0 | 0 |  |
| Hypocalcaemia | 0 | 0 | 0 | 0 | 0 | 0 | 1 | | 0 | 0 |  |
| Hypomagnesaemia | 2 | 0 | 0 | 1 | 2 | 0 | 1 | | 0 | 0 |  |
| Hyponatraemia | 0 | 0 | 0 | 1 | 0 | 0 | 0 | | 0 | 0 |  |
| Hypotension | 0 | 0 | 0 | 3 | 0 | 0 | 1 | | 0 | 0 |  |
| Hypothyroidism | 2 | 0 | 0 | 4 | 0 | 0 | 4 | | 0 | 0 |  |
| Ileal perforation | 0 | 0 | 0 | 0 | 1 | 0 | 0 | | 0 | 0 |  |
| Infected neoplasm | 0 | 0 | 0 | 0 | 1 | 0 | 0 | | 0 | 0 |  |
| Infection | 0 | 1 | 0 | 0 | 0 | 0 | 1 | | 0 | 0 |  |
| Influenza-like illness | 0 | 0 | 0 | 0 | 0 | 0 | 1 | | 0 | 0 |  |
| Infusion related reaction | 1 | 0 | 0 | 1 | 0 | 0 | 0 | | 0 | 0 |  |
| Insomnia | 1 | 0 | 0 | 3 | 0 | 0 | 2 | | 0 | 0 |  |
| Intestinal obstruction | 0 | 0 | 0 | 0 | 0 | 0 | 1 | | 0 | 0 |  |
| Intra-abdominal haemorrhage | 0 | 0 | 0 | 1 | 0 | 0 | 0 | | 0 | 0 |  |
| Intraocular pressure increased | 0 | 0 | 0 | 0 | 0 | 0 | 1 | | 0 | 0 |  |
| Irritable bowel syndrome | 1 | 0 | 0 | 0 | 0 | 0 | 0 | | 0 | 0 |  |
| Joint swelling | 1 | 0 | 0 | 0 | 0 | 0 | 0 | | 0 | 0 |  |
| Keratitis | 0 | 0 | 0 | 1 | 0 | 0 | 0 | | 0 | 0 |  |
| Lacrimation increased | 0 | 0 | 0 | 1 | 0 | 0 | 0 | | 0 | 0 |  |
| Large intestinal obstruction | 0 | 0 | 0 | 1 | 0 | 0 | 0 | | 0 | 0 |  |
| Lethargy | 4 | 0 | 0 | 0 | 0 | 0 | 2 | | 0 | 0 |  |
| Leukopenia | 0 | 0 | 0 | 2 | 0 | 0 | 0 | | 0 | 0 |  |
| Lichen sclerosis | 0 | 0 | 0 | 0 | 0 | 0 | 1 | | 0 | 0 |  |
| Ligament sprain | 0 | 0 | 0 | 1 | 0 | 0 | 0 | | 0 | 0 |  |
| Lip swelling | 0 | 0 | 0 | 0 | 0 | 0 | 1 | | 0 | 0 |  |
| Lipoma | 1 | 0 | 0 | 0 | 0 | 0 | 0 | | 0 | 0 |  |
| Lower respiratory tract infection | 1 | 1 | 0 | 0 | 0 | 0 | 0 | | 0 | 0 |  |
| Lymphocyte count decreased | 0 | 0 | 0 | 0 | 1 | 0 | 0 | | 0 | 0 |  |
| Lymphoedema | 1 | 0 | 0 | 0 | 0 | 0 | 0 | | 0 | 0 |  |
| Lymphopenia | 0 | 0 | 0 | 1 | 0 | 0 | 0 | | 0 | 0 |  |
| Migraine | 0 | 0 | 0 | 0 | 0 | 0 | 1 | | 0 | 0 |  |
| Mobility decreased | 1 | 0 | 0 | 0 | 0 | 0 | 0 | | 0 | 0 |  |
| Monoparesis | 0 | 0 | 0 | 1 | 0 | 0 | 0 | | 0 | 0 |  |
| Mouth ulceration | 0 | 0 | 0 | 3 | 0 | 0 | 0 | | 0 | 0 |  |
| Mucosal inflammation | 0 | 0 | 0 | 0 | 0 | 0 | 1 | | 0 | 0 |  |
| Mucositis oral | 2 | 0 | 0 | 12 | 1 | 0 | 6 | | 0 | 0 |  |
| Muscle spasms | 0 | 0 | 0 | 2 | 0 | 0 | 0 | | 0 | 0 |  |
| Muscular weakness | 1 | 0 | 0 | 0 | 0 | 0 | 1 | | 0 | 0 |  |
| Musculoskeletal chest pain | 2 | 0 | 0 | 3 | 0 | 0 | 1 | | 0 | 0 |  |
| Musculoskeletal pain | 2 | 0 | 0 | 2 | 0 | 0 | 4 | | 0 | 0 |  |
| Myalgia | 2 | 0 | 0 | 4 | 0 | 0 | 3 | | 0 | 0 |  |
| Myocardial infarction | 1 | 0 | 0 | 0 | 0 | 0 | 0 | | 0 | 0 |  |
| Nail discoloration | 1 | 0 | 0 | 2 | 0 | 0 | 0 | | 0 | 0 |  |
| Nail disorder | 2 | 0 | 0 | 2 | 0 | 0 | 0 | | 0 | 0 |  |
| Nail dystrophy | 0 | 0 | 0 | 1 | 0 | 0 | 0 | | 0 | 0 |  |
| Nasal congestion | 0 | 0 | 0 | 1 | 0 | 0 | 0 | | 0 | 0 |  |
| Nasal discomfort | 0 | 0 | 0 | 2 | 0 | 0 | 0 | | 0 | 0 |  |
| Nasopharyngitis | 0 | 0 | 0 | 1 | 0 | 0 | 1 | | 0 | 0 |  |
| Nausea | 19 | 0 | 0 | 18 | 2 | 0 | 30 | | 0 | 0 |  |
| Neck pain | 1 | 1 | 0 | 0 | 0 | 0 | 1 | | 0 | 0 |  |
| Neurotoxicity | 1 | 0 | 0 | 4 | 1 | 0 | 7 | | 0 | 0 |  |
| Neutrophil count decreased | 5 | 2 | 0 | 6 | 5 | 0 | 3 | | 0 | 0 |  |
| Nocturia | 1 | 0 | 0 | 0 | 0 | 0 | 0 | | 0 | 0 |  |
| Non-cardiac chest pain | 1 | 0 | 0 | 0 | 0 | 0 | 0 | | 0 | 0 |  |
| Oedema | 0 | 0 | 0 | 1 | 0 | 0 | 0 | | 0 | 0 |  |
| Oedema peripheral | 4 | 0 | 0 | 3 | 0 | 0 | 2 | | 0 | 0 |  |
| Onychomadesis | 0 | 0 | 0 | 1 | 0 | 0 | 0 | | 0 | 0 |  |
| Oral candidiasis | 0 | 0 | 0 | 1 | 0 | 0 | 0 | | 0 | 0 |  |
| Oral dysaesthesia | 0 | 0 | 0 | 0 | 0 | 0 | 1 | | 0 | 0 |  |
| Oral herpes | 0 | 0 | 0 | 1 | 0 | 0 | 0 | | 0 | 0 |  |
| Oral pain | 0 | 0 | 0 | 2 | 0 | 0 | 0 | | 0 | 0 |  |
| Oropharyngeal pain | 0 | 0 | 0 | 2 | 0 | 0 | 2 | | 0 | 0 |  |
| Osteoarthritis | 1 | 0 | 0 | 0 | 0 | 0 | 0 | | 0 | 0 |  |
| Pain | 6 | 0 | 0 | 5 | 0 | 0 | 4 | | 0 | 0 |  |
| Pain in extremity | 5 | 0 | 0 | 3 | 0 | 0 | 4 | | 0 | 0 |  |
| Palpitations | 1 | 0 | 0 | 1 | 0 | 0 | 0 | | 0 | 0 |  |
| Pancytopenia | 0 | 1 | 0 | 0 | 0 | 0 | 0 | | 0 | 0 |  |
| Pelvic pain | 1 | 0 | 0 | 2 | 0 | 0 | 1 | | 0 | 0 |  |
| Pericardial effusion | 0 | 0 | 0 | 0 | 0 | 0 | 0 | | 1 | 0 |  |
| Perineal pain | 0 | 0 | 0 | 0 | 0 | 0 | 1 | | 0 | 0 |  |
| Periorbital oedema | 1 | 0 | 0 | 0 | 0 | 0 | 0 | | 0 | 0 |  |
| Peripheral motor neuropathy | 3 | 0 | 0 | 0 | 0 | 0 | 1 | | 0 | 0 |  |
| Peripheral sensory neuropathy | 20 | 0 | 0 | 19 | 0 | 0 | 6 | | 0 | 0 |  |
| Peripheral swelling | 3 | 0 | 0 | 2 | 0 | 0 | 1 | | 0 | 0 |  |
| Pharyngeal inflammation | 1 | 0 | 0 | 0 | 0 | 0 | 0 | | 0 | 0 |  |
| Platelet count decreased | 2 | 1 | 0 | 1 | 0 | 0 | 1 | | 0 | 0 |  |
| Pleural effusion | 0 | 1 | 0 | 0 | 0 | 0 | 0 | | 1 | 0 |  |
| Pneumonia | 0 | 0 | 0 | 0 | 0 | 0 | 0 | | 1 | 0 |  |
| Pneumonitis | 0 | 0 | 0 | 2 | 0 | 0 | 0 | | 0 | 0 |  |
| Pollakiuria | 1 | 0 | 0 | 3 | 0 | 0 | 0 | | 0 | 0 |  |
| Polycystic ovaries | 1 | 0 | 0 | 0 | 0 | 0 | 0 | | 0 | 0 |  |
| PPE syndrome | 0 | 0 | 0 | 1 | 0 | 0 | 0 | | 0 | 0 |  |
| Proctalgia | 0 | 0 | 0 | 0 | 0 | 0 | 2 | | 0 | 0 |  |
| Proteinuria | 1 | 0 | 0 | 2 | 0 | 0 | 0 | | 0 | 0 |  |
| Pruritus | 0 | 0 | 0 | 5 | 0 | 0 | 1 | | 0 | 0 |  |
| Pulmonary embolism | 2 | 0 | 0 | 0 | 1 | 0 | 0 | | 0 | 0 |  |
| Pustule | 0 | 0 | 0 | 1 | 0 | 0 | 0 | | 0 | 0 |  |
| Pyrexia | 1 | 0 | 0 | 2 | 2 | 0 | 1 | | 0 | 0 |  |
| Rash | 2 | 0 | 0 | 4 | 0 | 0 | 4 | | 0 | 0 |  |
| Rash erythematous | 0 | 0 | 0 | 1 | 0 | 0 | 0 | | 0 | 0 |  |
| Rash maculo-papular | 1 | 0 | 0 | 3 | 0 | 0 | 1 | | 0 | 0 |  |
| Rectal haemorrhage | 0 | 0 | 0 | 1 | 0 | 0 | 0 | | 0 | 0 |  |
| Respiratory tract infection | 0 | 0 | 0 | 2 | 0 | 0 | 0 | | 0 | 0 |  |
| Restless leg syndrome | 1 | 0 | 0 | 0 | 0 | 0 | 0 | | 0 | 0 |  |
| Rhinitis | 1 | 0 | 0 | 1 | 0 | 0 | 0 | | 0 | 0 |  |
| Sciatica | 0 | 0 | 0 | 1 | 0 | 0 | 1 | | 0 | 0 |  |
| Seasonal allergy | 0 | 0 | 0 | 2 | 0 | 0 | 0 | | 0 | 0 |  |
| Seizure | 1 | 0 | 0 | 1 | 0 | 0 | 0 | | 0 | 0 |  |
| Sepsis | 0 | 2 | 0 | 0 | 0 | 0 | 0 | | 0 | 0 |  |
| Sinus pain | 0 | 0 | 0 | 0 | 0 | 0 | 1 | | 0 | 0 |  |
| Sinusitis | 0 | 0 | 0 | 0 | 0 | 0 | 1 | | 0 | 0 |  |
| Skin fissures | 1 | 0 | 0 | 0 | 0 | 0 | 0 | | 0 | 0 |  |
| Skin infection | 0 | 0 | 0 | 1 | 0 | 0 | 0 | | 0 | 0 |  |
| Skin irritation | 1 | 0 | 0 | 0 | 0 | 0 | 0 | | 0 | 0 |  |
| Skin mass | 1 | 0 | 0 | 0 | 0 | 0 | 0 | | 0 | 0 |  |
| Skin necrosis | 0 | 0 | 0 | 0 | 0 | 0 | 1 | | 0 | 0 |  |
| Skin rash | 0 | 0 | 0 | 1 | 0 | 0 | 0 | | 0 | 0 |  |
| Skin ulcer | 0 | 0 | 0 | 2 | 0 | 0 | 1 | | 0 | 0 |  |
| Small intestinal obstruction | 0 | 0 | 0 | 0 | 0 | 0 | 0 | | 1 | 0 |  |
| Sneezing | 0 | 0 | 0 | 1 | 0 | 0 | 0 | | 0 | 0 |  |
| Stomatitis | 0 | 0 | 0 | 2 | 0 | 0 | 2 | | 0 | 0 |  |
| Stress fracture | 1 | 0 | 0 | 0 | 0 | 0 | 0 | | 0 | 0 |  |
| Swelling face | 1 | 0 | 0 | 0 | 0 | 0 | 0 | | 0 | 0 |  |
| Syncope | 0 | 0 | 0 | 1 | 1 | 0 | 0 | | 0 | 0 |  |
| Tachycardia | 0 | 0 | 0 | 1 | 0 | 0 | 0 | | 0 | 0 |  |
| Tendon rupture | 1 | 0 | 0 | 0 | 0 | 0 | 0 | | 0 | 0 |  |
| Thromboembolic event | 1 | 1 | 0 | 2 | 0 | 0 | 2 | | 2 | 0 |  |
| Thrombophlebitis superficial | 0 | 0 | 0 | 1 | 0 | 0 | 0 | | 0 | 0 |  |
| Thrombosis | 0 | 0 | 0 | 1 | 0 | 0 | 0 | | 0 | 0 |  |
| Tooth abscess | 0 | 0 | 0 | 1 | 0 | 0 | 0 | | 1 | 0 |  |
| Tooth repair | 0 | 0 | 0 | 1 | 0 | 0 | 0 | | 0 | 0 |  |
| Toothache | 1 | 0 | 0 | 0 | 0 | 0 | 1 | | 0 | 0 |  |
| Transient ischaemic attack | 0 | 0 | 0 | 0 | 1 | 0 | 0 | | 0 | 0 |  |
| Troponin increased | 0 | 0 | 0 | 0 | 0 | 0 | 1 | | 0 | 0 |  |
| Tumour pain | 1 | 0 | 0 | 0 | 0 | 0 | 0 | | 0 | 0 |  |
| Ulcerative keratitis | 0 | 0 | 0 | 1 | 0 | 0 | 0 | | 0 | 0 |  |
| Urinary incontinence | 1 | 0 | 0 | 1 | 0 | 0 | 0 | | 0 | 0 |  |
| Urinary tract disorder | 0 | 0 | 0 | 1 | 0 | 0 | 0 | | 0 | 0 |  |
| Urinary tract infection | 4 | 2 | 0 | 7 | 2 | 0 | 9 | | 0 | 0 |  |
| Urinary tract obstruction | 0 | 0 | 0 | 0 | 1 | 0 | 0 | | 0 | 0 |  |
| Vaginal discharge | 2 | 0 | 0 | 1 | 0 | 0 | 0 | | 0 | 0 |  |
| Vaginal haemorrhage | 4 | 0 | 0 | 4 | 0 | 0 | 1 | | 0 | 0 |  |
| Viral infection | 0 | 0 | 0 | 0 | 0 | 0 | 1 | | 0 | 0 |  |
| Vision blurred | 0 | 0 | 0 | 1 | 0 | 0 | 0 | | 0 | 0 |  |
| Vitamin B12 deficiency | 0 | 0 | 0 | 0 | 0 | 0 | 1 | | 0 | 0 |  |
| Vomiting | 6 | 0 | 0 | 11 | 1 | 0 | 18 | | 0 | 0 |  |
| Vulvovaginal discharge | 0 | 0 | 0 | 1 | 0 | 0 | 0 | | 0 | 0 |  |
| Weight decreased | 1 | 0 | 0 | 3 | 0 | 0 | 4 | | 0 | 0 |  |
| White blood cell count decreased | 2 | 0 | 0 | 1 | 0 | 0 | 0 | | 0 | 0 |  |

## Supplementary Figure S1: Adverse events of any grade experienced in ≥10% of patients.


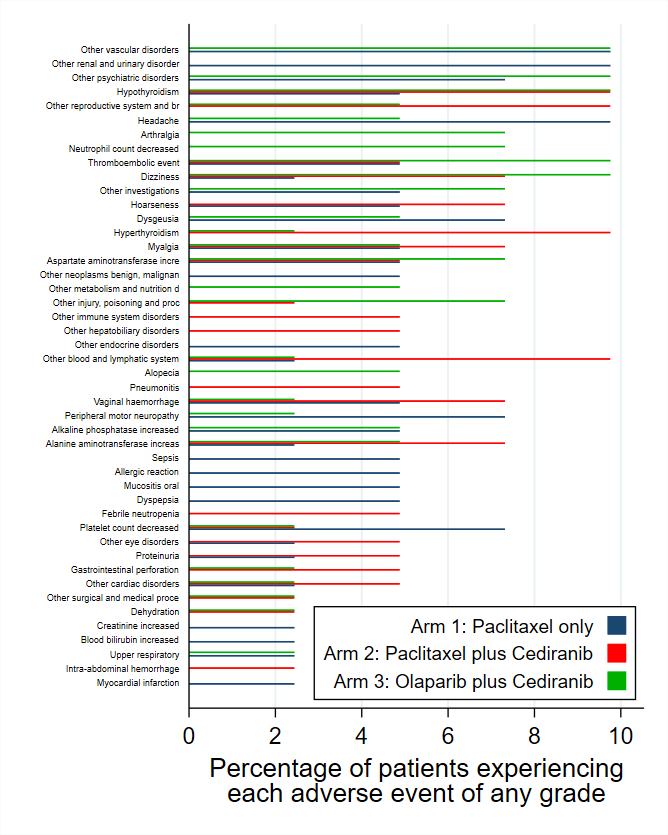


## Supplementary Figure S2: Adverse events of any grade experienced in <10% of patients.


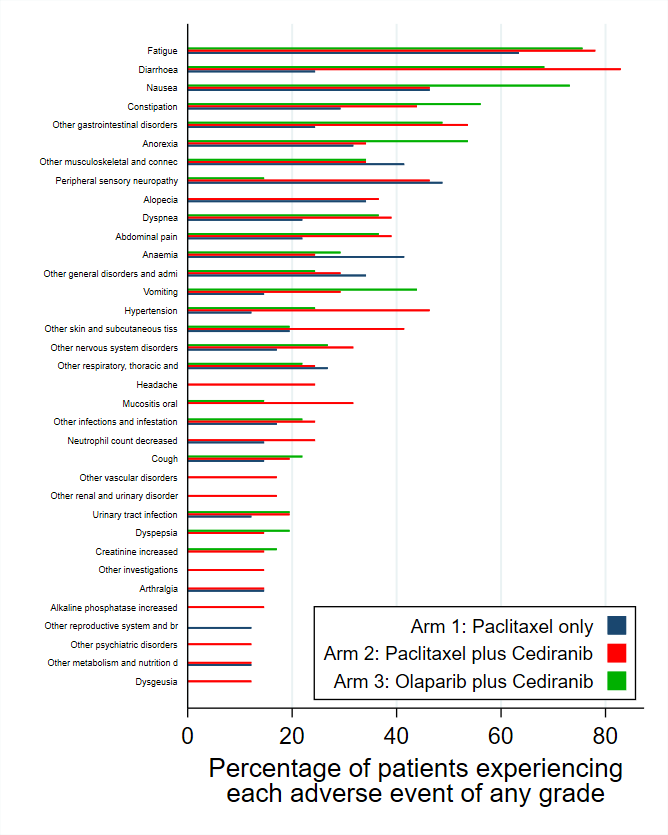


## Supplementary Table S3: Frequency of Serious Adverse Events and Reactions

Key: Serious adverse events (SAEs), serious adverse reactions (SARs) and suspected unexpected serious adverse reactions (SUSARs) by trial arm.

| **Event** | **Arm 1**  Paclitaxel | **Arm 2**  Paclitaxel/cediranib | **Arm 3**  Olaparib/cediranib |
| --- | --- | --- | --- |
| SAEs | 13 (32%) | 9 (22%) | 8 (19%) |
| SARs | 3 (7%) | 9 (22%) | 1 (2%) |
| SUSARs | 1 (2%) | 8 (20%) | 5 (12%) |

## Supplementary Table S4: Serious Adverse Events

| **Trial Arm** | **Serious Adverse Event** | **CTCAE grade** |
| --- | --- | --- |
| **Arm 1**  Paclitaxel | Brain metastases | 2 |
|  | COVID-19 | 3 |
|  | Embolism | 3 |
|  | Fistula | 3 |
|  | Neck pain | 3 |
|  | Pharyngeal inflammation | 1 |
|  | Pleural effusion | 3 |
|  | Pleural effusion | 3 |
|  | Seizure | 2 |
|  | Stress fractures | 2 |
|  | Urinary tract infection | 3 |
|  | Urinary tract infection | 3 |
|  | Vomiting | 1 |
| **Arm 2**  Paclitaxel/cediranib | Abdominal pain | 3 |
|  | Dehydration | 3 |
|  | Diarrhoea | 3 |
|  | Dyspnoea | 3 |
|  | Febrile neutropenia | 3 |
|  | Infection | 3 |
|  | Intestinal perforation | 5 |
|  | Pyrexia | 3 |
|  | Urinary tract obstruction | 3 |
|  | Vaginal haemorrhage | 2 |
| **Arm 3**  Olaparib/cediranib | Abdominal pain | 3 |
|  | Ascites | 3 |
|  | Confusional state | 3 |
|  | Fall | 3 |
|  | Fall | 3 |
|  | Small intestinal obstruction | 3 |
|  | Small intestinal obstruction | 4 |
|  | Troponin increased | 1 |

## Supplementary Table S5: Serious Adverse Reactions

| **Trial Arm** | **Serious Adverse Reactions** | **CTCAE grade** |
| --- | --- | --- |
| **Arm 1**  Paclitaxel | Infection | 3 |
|  | Sepsis | 2 |
|  | Sepsis | 3 |
| **Arm 2**  Paclitaxel/cediranib | Anaemia | 2 |
|  | Diarrhoea | 1 |
|  | Diarrhoea | 3 |
|  | Hypertension | 3 |
|  | Hypertension | 3 |
|  | Intestinal perforation | 3 |
|  | Pyrexia | 1 |
|  | Transient ischemic attack | 2 |
|  | Urinary tract infection | 3 |
| **Arm 3**  Olaparib/cediranib | Fatigue | 2 |

## Supplementary Table S6: Suspected Unexpected Serious Adverse Reactions

| **Trial Arm** | **Suspected Unexpected Serious Adverse Reactions** | **CTCAE grade** |
| --- | --- | --- |
| **Arm 1**  Paclitaxel | Device related infection | 3 |
| **Arm 2**  Paclitaxel/cediranib | Abnormal liver function tests | 3 |
|  | Acute kidney injury | 1 |
|  | Fatigue | 3 |
|  | Fatigue | 3 |
|  | Headache | 3 |
|  | Headache | 2 |
|  | Pneumonitis | 2 |
|  | Seizure | 2 |
| **Arm 3**  Olaparib/cediranib | Cerebrovascular accident | 3 |
|  | Dehydration | 3 |
|  | Intestinal perforation | 3 |
|  | Pneumonia | 3 |
|  | Soft tissue necrosis | 2 |

## Supplementary Table S7: Health-related quality-of-life data

A confidence interval (CI) that crosses 0 indicates that there is no evidence to reject the null hypotheses of no difference in the adjusted means; a CI that is completely <0 indicates that there is evidence that the mean score decreases over time or is lower in the treatment group; a CI that is completely >0 indicates that there is evidence that the mean score increases over time or is higher in the treatment group; Arm 1 (paclitaxel) = control group; Arm 2 (paclitaxel/cediranib) and Arm 3 (olaparib/cediranib) = treatment groups.

| **EORTC QLQ-C30**  **and -EN24 scales** | **Comparison Arm 2 or Arm 3 versus Arm 1 (paclitaxel only)** | **Effect** | | | | **Treatment × Time effect**  **(P value)** |
| --- | --- | --- | --- | --- | --- | --- |
|  |  | **Treatment** | | **Time** | |  |
|  |  | **Adjusted mean difference (95% CI)** | **P value** | **Adjusted mean difference (95% CI)** | **P value** |  |
| Global health status | Arm 2 (paclitaxel/cediranib) | 2.5 (-2.3-7.3) | 0.314 | 0.1 (0.0-0.2) | 0.046 | 0.289 |
|  | Arm 3 (olaparib/cediranib) | 2.3 (-0.3-5.0) | 0.080 | 0.1 (-0.0-0.2) | 0.050 | <0.001 |
| Physical functioning | Arm 2 | 0.7 (-4.0-5.3) | 0.776 | 0.2 (0.1-0.2) | <0.001 | 0.169 |
|  | Arm 3 | 2.3 (-0.1-4.7) | 0.059 | 0.2 (0.1-0.2) | <0.001 | <0.001 |
| Role functioning | Arm 2 | 5.8 (-0.4-11.9) | 0.069 | 0.2 (0.1-0.3) | <0.001 | 0.003 |
|  | Arm 3 | 4.4 (0.8-8.1) | 0.017 | 0.2 (0.1-0.3) | <0.001 | <0.001 |
| Emotional functioning | Arm 2 | 1.2 (-3.0-5.3) | 0.584 | 0.1 (0.1-0.2) | <0.001 | 0.345 |
|  | Arm 3 | -1.0 (-3.0-1.0) | 0.331 | 0.1 (0.1-0.2) | <0.001 | <0.001 |
| Cognitive functioning | Arm 2 | -0.7 (-4.9-3.4) | 0.727 | -0.0 (-0.1-0.0) | 0.239 | 0.615 |
|  | Arm 3 | -0.4 (-2.3-1.6) | 0.715 | -0.0 (-0.1-0.0) | 0.18 | 0.005 |
| Social functioning | Arm 2 | 1.3 (-5.2-7.7) | 0.701 | 0.2 (0.1-0.3) | <0.001 | <0.001 |
|  | Arm 3 | 0.4 (-3.0-3.8) | 0.819 | 0.2 (0.1-0.3) | <0.001 | <0.001 |
| Fatigue | Arm 2 | -2.3 (-7.8-3.2) | 0.405 | -0.1 (-0.2--0.0) | 0.028 | 0.742 |
|  | Arm 3 | -1.7 (-4.5-1.1) | 0.242 | -0.1 (-0.2--0.0) | 0.021 | <0.001 |
| Nausea and vomiting | Arm 2 | -0.9 (-4.9-3.1) | 0.662 | -0.0 (-0.1-0.1) | 0.793 | 0.941 |
|  | Arm 3 | 1.0 (-1.3-3.3) | 0.393 | -0.0 (-0.1-0.1) | 0.822 | <0.001 |
| Pain | Arm 2 | -4.7 (-10.7-1.3) | 0.125 | -0.2 (-0.3--0.1) | 0.002 | 0.085 |
|  | Arm 3 | -1.0 (-4.4-2.3) | 0.547 | -0.2 (-0.3--0.1) | <0.001 | <0.001 |
| Dyspnoea | Arm 2 | -0.1 (-6.9-6.6) | 0.969 | -0.0 (-0.1-0.1) | 0.674 | 0.881 |
|  | Arm 3 | -2.7 (-6.1-0.7) | 0.121 | -0.0 (-0.1-0.1) | 0.807 | 0.027 |
| Insomnia | Arm 2 | -0.7 (-5.7-4.3) | 0.791 | -0.1 (-0.2-0.0) | 0.251 | 0.614 |
|  | Arm 3 | -1.5 (-4.5-1.6) | 0.342 | -0.1 (-0.2-0.0) | 0.274 | 0.112 |
| Appetite loss | Arm 2 | 2.4 (-5.0-9.7) | 0.526 | -0.1 (-0.2-0.1) | 0.283 | 0.793 |
|  | Arm 3 | -0.1 (-4.3-4.1) | 0.953 | -0.1 (-0.2-0.1) | 0.328 | <0.001 |
| Constipation | Arm 2 | -3.7 (-10.4-3.1) | 0.288 | -0.1 (-0.2-0.0) | 0.074 | 0.022 |
|  | Arm 3 | -0.8 (-3.4-1.7) | 0.533 | -0.1 (-0.2-0.0) | 0.077 | 0.344 |
| Diarrhoea | Arm 2 | 11.5 (5.9-17.1) | <0.001 | -0.0 (-0.1-0.1) | 0.578 | <0.001 |
|  | Arm 3 | 2.4 (-0.6-5.4) | 0.123 | -0.0 (-0.1-0.1) | 0.830 | <0.001 |
| Financial difficulties | Arm 2 | 2.5 (-2.5-7.5) | 0.334 | -0.0 (-0.1-0.0) | 0.364 | 0.686 |
|  | Arm 3 | 1.3 (-0.8-3.3) | 0.234 | -0.0 (-0.1-0.0) | 0.347 | 0.028 |
| Lymphoedema | Arm 2 | -3.7 (-8.2-0.9) | 0.114 | 0.0 (-0.0-0.1) | 0.238 | 0.022 |
|  | Arm 3 | -0.8 (-3.3-1.6) | 0.502 | 0.0 (-0.0-0.1) | 0.333 | 0.425 |
| Urological symptoms | Arm 2 | -2.6 (-6.7-1.4) | 0.205 | -0.2 (-0.2--0.1) | <0.001 | 0.007 |
|  | Arm 3 | -0.5 (-2.4-1.4) | 0.591 | -0.2 (-0.2--0.1) | <0.001 | <0.001 |
| Gastrointestinal symptoms | Arm 2 | 2.5 (-0.7-5.6) | 0.121 | 0.0 (-0.0-0.1) | 0.793 | <0.001 |
|  | Arm 3 | 1.0 (-0.6-2.6) | 0.208 | 0.0 (-0.0-0.1) | 0.662 | 0.015 |
| Body image | Arm 2 | -2.6 (-9.2-4.1) | 0.450 | 0.0 (-0.0-0.1) | 0.297 | 0.301 |
|  | Arm 3 | -5.1 (-8.3--1.8) | 0.002 | 0.0 (-0.0-0.1) | 0.285 | 0.023 |
| Sexual/vaginal problems | Arm 2 | -2.0 (-9.0-4.9) | 0.567 | -0.5 (-1.8-0.7) | 0.386 | 0.175 |
|  | Arm 3 | -0.3 (-4.5-3.9) | 0.879 | -0.1 (-2.1-1.9) | 0.896 | 0.794 |
| Back/pelvic pain | Arm 2 | -1.2 (-10.1-7.8) | 0.797 | -0.2 (-0.3--0.1) | 0.005 | 0.483 |
|  | Arm 3 | -0.8 (-4.8-3.2) | 0.699 | -0.2 (-0.3--0.0) | 0.007 | 0.003 |
| Tingling/numbness | Arm 2 | -5.7 (-13.1-1.7) | 0.132 | 0.1 (0.0-0.2) | 0.010 | 0.325 |
|  | Arm 3 | -3.2 (-6.7-0.3) | 0.073 | 0.1 (0.0-0.2) | 0.004 | 0.003 |
| Muscular/joint pain | Arm 2 | -2.7 (-9.5-4.1) | 0.442 | -0.1 (-0.2-0.0) | 0.061 | 0.445 |
|  | Arm 3 | -0.1 (-3.8-3.5) | 0.937 | -0.1 (-0.2--0.0) | 0.031 | <0.001 |
| Hair loss | Arm 2 | -11.3 (-23.4-0.8) | 0.067 | -0.1 (-0.3-0.1) | 0.463 | 0.892 |
|  | Arm 3 | -15.4 (-20.3--10.5) | <0.001 | -0.1 (-0.2-0.1) | 0.498 | 0.380 |
| Taste change | Arm 2 | 7.8 (-0.6-16.1) | 0.067 | -0.1 (-0.2-0.0) | 0.124 | 0.736 |
|  | Arm 3 | -1.4 (-5.5-2.7) | 0.499 | -0.1 (-0.2-0.0) | 0.087 | <0.001 |
| Sexual interest | Arm 2 | 2.0 (-1.2-5.3) | 0.225 | -0.0 (-0.1-0.0) | 0.479 | 0.759 |
|  | Arm 3 | 0.5 (-1.0-1.9) | 0.520 | -0.0 (-0.1-0.0) | 0.295 | 0.143 |
| Sexual activity | Arm 2 | -0.2 (-3.1-2.6) | 0.879 | -0.0 (-0.0-0.0) | 0.830 | 0.985 |
|  | Arm 3 | 0.5 (-0.8-1.8) | 0.416 | -0.0 (-0.0-0.0) | 0.739 | 0.056 |
| Sexual enjoyment | Arm 2 | 34.2 (10.4-58.0) | 0.005 | -0.5 (-3.4-2.4) | 0.726 | 0.604 |
|  | Arm 3 | 2.8 (-3.8-9.5) | 0.403 | -0.7 (-4.0-2.7) | 0.699 | 0.163 |

## Supplementary Table S8: Molecular subtyping

Data are presented as number (percent; denominated is ‘*Total*’ for that Arm); dMMR, mismatch repair deficient; NSMP, no specific mutational pattern; p53abn, mutant p53 expression. The molecular subtype was unknown in 34 patients (19 did not optionally agree to donate archival tissue, 12 had tissue blocks that contained no tumour and 3 failed immunohistochemistry testing). There was no significant association between molecular subtype and trial arm (p=0·58).

| **Molecular subtype** | **Arm 1**  Paclitaxel | **Arm 2**  Paclitaxel/cediranib | **Arm 3**  Olaparib/cediranib |
| --- | --- | --- | --- |
| dMMR | 5 (12%) | 9 (22%) | 4 (10%) |
| p53abn | 19 (46%) | 12 (29%) | 17 (40%) |
| NSMP | 8 (20%) | 8 (20%) | 8 (19%) |
| Unknown | 9 (22%) | 12 (29%) | 13 (31%) |

## Supplementary Table S9: Association between clinical variables and PFS at 3 months

Chi‑squared tests were used to explore associations between clinical variables and the primary endpoint, PFS at 3 months. Age was dichotomised at the median, and category definitions for all other clinical variables are provided in Supplementary Table S13. Although molecular subtype showed only borderline evidence of association with PFS at 3 months in univariable analysis, it was significantly associated with the primary endpoint in multivariable analysis (Supplementary Table S10).

| **Clinical variable** | **Association with PFS at 3 months**  **(p value, chi-squared test)** |
| --- | --- |
| Age | 0·334 |
| ECOG performance status | 0·199 |
| FIGO stage | 0·443 |
| Histological subtype | 0·287 |
| Histological grade | 0·031 |
| Molecular subtype | 0·096 |
| Prior chemotherapy | 0·351 |
| Prior radiotherapy | 0·306 |
| Prior surgery | 0·388 |

## Supplementary Table S10: Multivariable logistic regression analysis of PFS at 3 months

Key: dMMR, mismatch repair deficient; NSMP, no specific mutational pattern; p53abn, mutant p53 expression. All variables shown in Supplementary Table S9 were included in this model regardless of the association between PFS at 3 months. Histological grade was not evaluable in the multivariable logistic model because it correlated highly with molecular subtype.

| **Variable** | **PFS at 3 months** | | |
| --- | --- | --- | --- |
|  | **Odds ratio** | **95% CI** | **P value** |
| **Treatment arm**  Paclitaxel/cediranib versus paclitaxel  Olaparib/cediranib versus paclitaxel | 4·17  1·00 | 1·45–13·10  0·39–2·59 | 0·011  0·999 |
| **Molecular subgroup**  p53abn versus dMMR  NSMP versus dMMR  Unknown versus dMMR | 6·55  3·79  4·37 | 1·79–27·33  0·95–16·74  1·16–18·31 | 0·006  0·066  0·034 |

## Supplementary Table S11: Average pre-treatment plasma Tie2 value in each trial arm.

Data are presented as mean (Log2) ± standard deviation. 106 patients had a pre-treatment measurable Tie2 value. The pre-treatment Tie2 value was the average of the plasma Tie2 concentration measured at the screening visit and cycle 1 day 1.

| **Arm 1**  Paclitaxel | **Arm 2**  Paclitaxel/cediranib | **Arm 3**  Olaparib/cediranib |
| --- | --- | --- |
| 15·4 ± 0·68 | 15·4 ± 0·49 | 15·5 ± 0·49 |

## Supplementary Table S12: Vascular response

A reduction in plasma Tie2 concentration of ≥5% within the first 9 weeks of trial treatment was defined as a vascular response. 72 were treated with cediranib in Arm 2 (n=34) or Arm 3 (n=38) were categorised as vascular responders or non-responders. The vascular non-responders group additionally included 19 patients who developed disease progression within the first 9 weeks of trial treatment (9 in Arm 2, 10 in Arm 3), leading to trial withdrawal. Of the 72 patients treated with cediranib in Arm 2 or Arm 3, 42 patients were categorised as vascular responders. The proportion of vascular responders in Arm 2 and Arm 3 did not differ significantly (p=0·89). The definition of vascular response does not apply to patients in Arm 1 (6/33 patients in Arm 1 had a reduction in plasma Tie2 of 5% or more).

| **Vascular responder** | **Arm 2**  Paclitaxel/cediranib | **Arm 3**  Olaparib/cediranib |
| --- | --- | --- |
| Yes | 20 (28%) | 22 (31%) |
| No | 14 (19%) | 16 (22%) |

##

## Supplementary Figure S3: Kaplan-Meier curves for PFS of cediranib-treated vascular response

42 patients treated with cediranib were classified as vascular responders (‘VEGFi Response’); 30 patients receiving cediranib were classified as vascular non responders (14 in Arm 2, 16 in Arm 3 [‘VEGFi No Response’]); 41 patients received paclitaxel only (Arm 1). The median PFS in the cediranib-treated vascular responders’ group was 8·4 months (95% CI 5·6–11·3). The median PFS in the cediranib-treated vascular non responders’ group was 5·9 months (95% CI 5·7–NA). The median PFS in paclitaxel only group was 5·5 months (95% CI 1·6–8·2). The p-value is derived using a two-sided log-rank test.


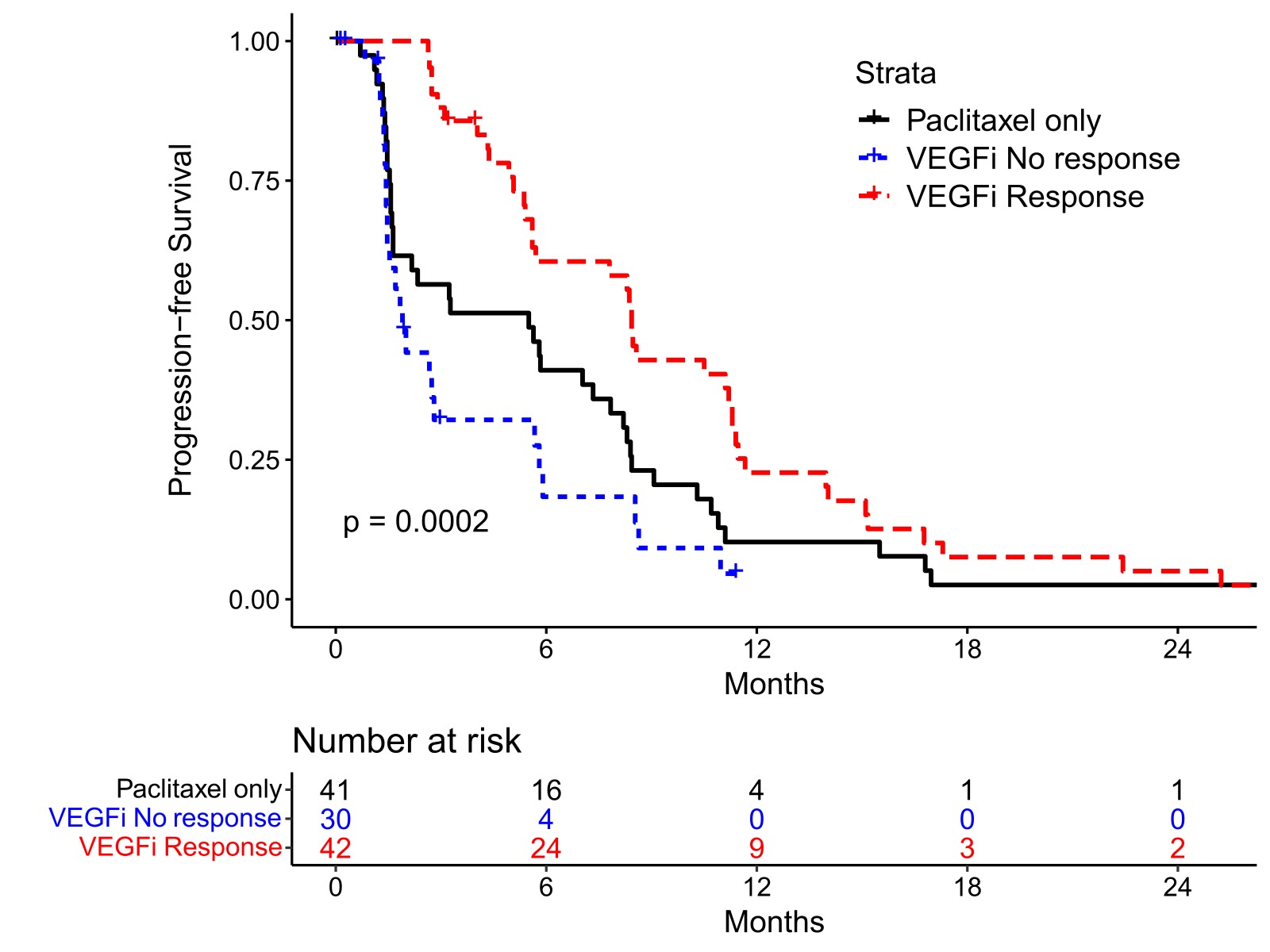


## Supplementary Figure S4: Kaplan-Meier curves for OS of cediranib-treated vascular response

42 patients treated with cediranib were classified as vascular responders (‘VEGFi Response’); 30 patients receiving cediranib were classified as vascular non responders (14 in Arm 2, 16 in Arm 3 [‘VEGFi No Response’]); 41 patients received paclitaxel only (Arm 1). The median OS in the in the cediranib-treated vascular responders’ group was 18·6 months (95% CI 14·1–31·0). The median OS in the cediranib-treated vascular non responders’ group was 9·0 months (95% CI 5·9–14·8). The median OS in paclitaxel group was 13·0 months (95% CI 8·5–17·7). The p-value is derived using a two-sided log-rank test.


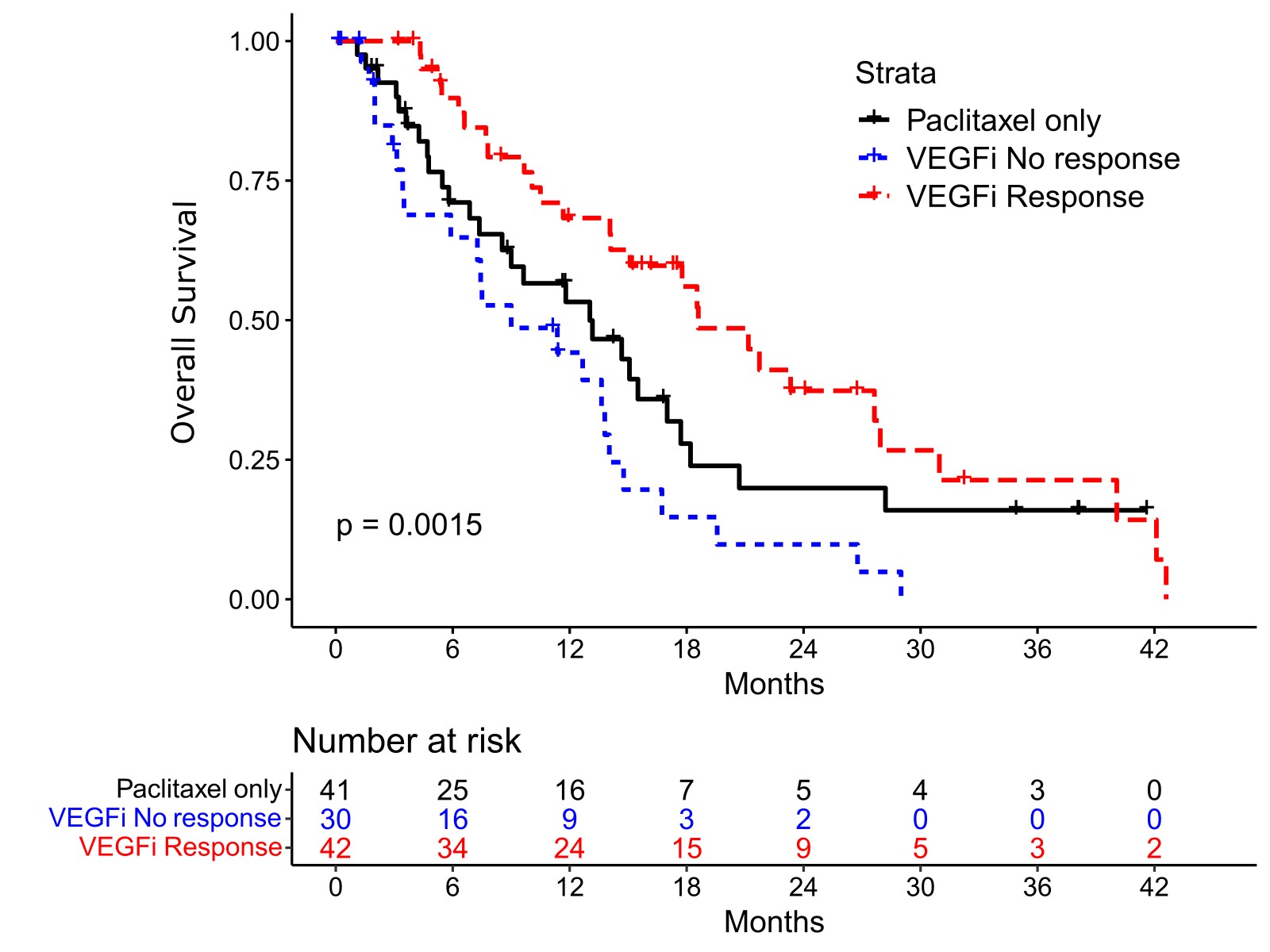


## Supplementary Table S13: Univariable Cox proportional hazards analysis for clinical variables at baseline

Univariable analysis found histological grade (PFS, OS), molecular subtype (PFS) and prior radiotherapy (PFS, OS) were prognostic in the 123 patients treated in COPELIA; dMMR, mismatch repair deficient; NSMP, no specific mutational pattern; p53abn, mutant p53 expression**.**

| **Clinical variable** | **PFS** | | **OS** | |
| --- | --- | --- | --- | --- |
|  | **Hazard ratio** | **P value** | **Hazard ratio** | **P value** |
| **Age** | 0·89 | 0·79 | 0·58 | 0·25 |
| **ECOG performance status**  0  1 | 1·00  1·20 | -  0·29 | 1·00  1·36 | -  0·18 |
| **FIGO stage**  I-II  III  IV | 1·00  0·95  1·29 | -  0·82  0·26 | 1·00  1·02  1·31 | -  0·95  0·30 |
| **Histological grade**  1  2  3 | 1·00  3·58  2·21 | -  0·004  0·03 | 1·00  4·94  2·60 | -  0·002  0·03 |
| **Histological subtype**  Carcinosarcoma  Endometrioid  Clear cell  Serous | 1·00  0·78  0·55  0·64 | -  0·42  0·24  0·18 | 1·00  0·57  0·44  0·56 | -  0·10  0·14  0·11 |
| **Molecular subtype**  dMMR  p53abn  NSMP  Unknown | 1·00  0·41  0·41  0·53 | -  0·003  0·008  0·04 | 1·00  0·90  0·66  0·85 | -  0·77  0·29  0·67 |
| **Prior surgery**  Yes  No | 1·00  1·30 | -  0·27 | 1·00  1·61 | -  0·07 |
| **Prior radiotherapy**  Yes  No | 1·00  1·43 | -  0·07 | 1·00  1·63 | -  0·03 |
| **Prior lines of cytotoxic chemotherapy not including adjuvant therapy**  0  1  2 | 1·00  1·39  1·59 | -  0·21  0·22 | 1·00  0·92  0·78 | -  0·77  0·56 |

## Supplementary Table S14: Multivariable Cox proportional hazards analysis for vascular response

Univariable analysis found histological grade (PFS, OS), molecular subtype (PFS) and prior radiotherapy (PFS, OS) were prognostic in the overall population (123 patients) (Supplementary Table S13). 42 patients receiving cediranib were classified as vascular responders; 30 patients receiving cediranib were classified as vascular non-responders; 41 patients receiving paclitaxel only were classified as controls. HR, hazard ratio; 95% CI, 95% confidence interval; dMMR, mismatch repair deficient; p53abn, mutant p53 expression.

| **Clinical variable** | **PFS** | |
| --- | --- | --- |
|  | **HR (95% CI)** | **P value** |
| **Vascular responder**  VEGFi vascular response versus paclitaxel | 0·54 (0·33–0·88) | 0·014 |
| VEGFi vascular non-responder versus paclitaxel | 1·63 (0·95–2·79) | 0.078 |
| **Histological grade**  2 versus 1  3 versus 1 | 3·83 (1·56–9·43)  2·77 (1·28–6·03] | 0·003  0·010 |
| **Molecular subtype**  p53abn versus dMMR  NSMP versus dMMR  Unknown versus dMMR | 0·32 (0·15–0·69)  0·70 (0·33–1·49)  0·51 (0·24–1·08) | 0·003  0·359  0·079 |
| **Prior radiotherapy**  No versus yes | 2·03 (1·29–3·19) | 0·002 |

| **Variable** | **OS** | |
| --- | --- | --- |
|  | **HR (95% CI)** | **P value** |
| **Vascular responder**  VEGFi vascular responder versus paclitaxel | 0·57 (0·32–1·01) | 0·052 |
| VEGFi vascular non-responder versus paclitaxel | 1·98 (1·12–3·51) | 0·019 |
| **Histological grade**  2 versus 1  3 versus 1 | 6·70 (2·29–19·59)  2·13 (0·89–5·09) | 0·001  0·088 |
| **Prior radiotherapy**  No versus yes | 1·87 (1·15–3·06) | 0·012 |

## Supplementary Figure S5: Additive PFS benefit in vascular responders receiving olaparib

Key: Chemo, paclitaxel only; VEGFi, cediranib; PARPi, olaparib. 42 patients treated with cediranib were classified as vascular responders; 30 patients receiving cediranib were classified as vascular non responders (14 in Arm 2, 16 in Arm 3). In vascular responders, an additional PFS benefit was observed in those receiving olaparib (p=0·026), while in vascular non-responders, a reduced PFS benefit was observed in those receiving olaparib (p=0·034).


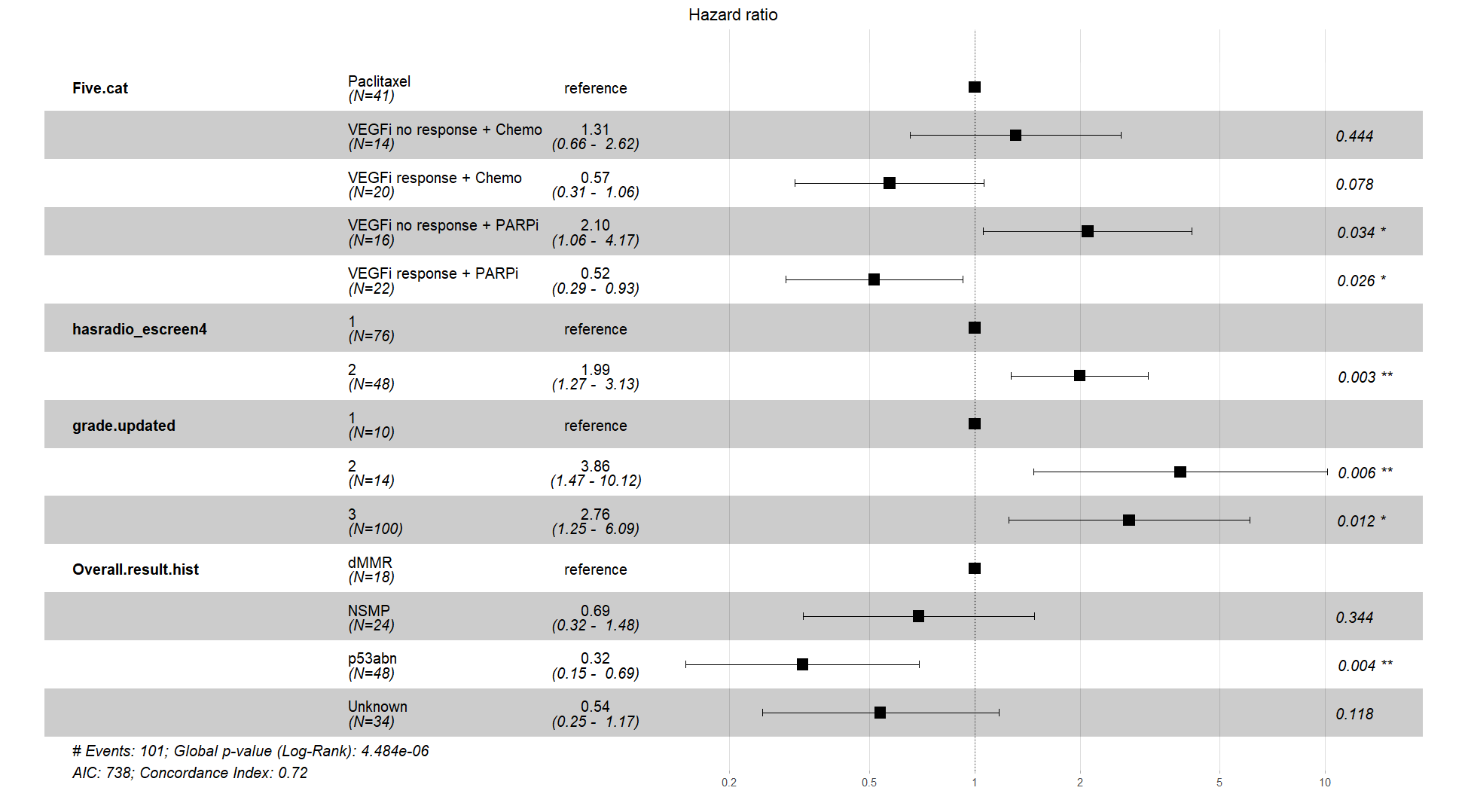


## Supplementary Figure S6: Additive OS benefit in vascular responders receiving olaparib

Key: Chemo, paclitaxel only; VEGFi, cediranib; PARPi, olaparib. 42 patients treated with cediranib were classified as vascular responders; 30 patients receiving cediranib were classified as vascular non responders (14 in Arm 2, 16 in Arm 3). In vascular responders, an additional OS benefit was observed in those receiving olaparib (p=0·023), while in vascular non-responders, a reduced OS benefit was observed in those receiving olaparib (p=0·028).

**
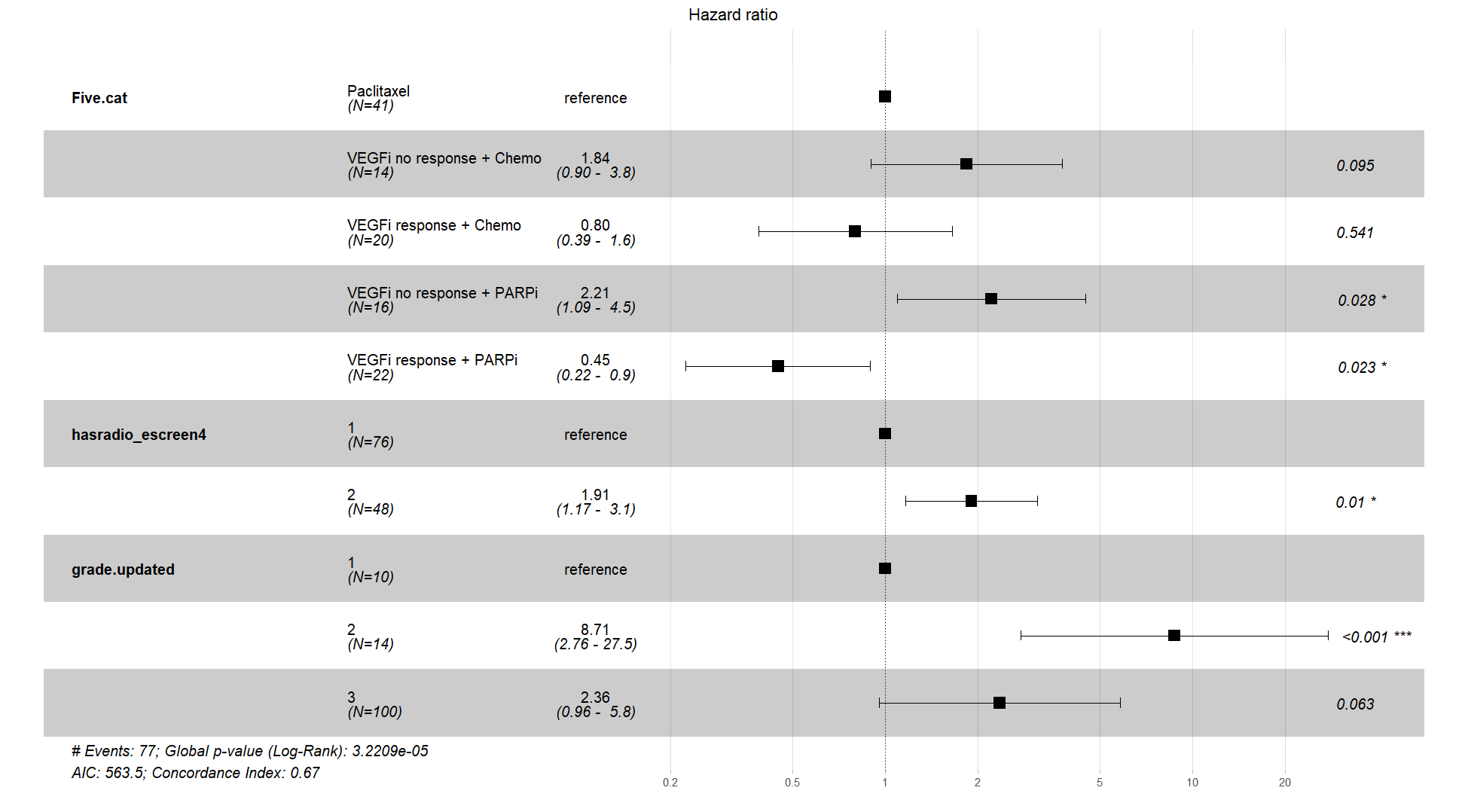
**

## Supplementary Table S15: Adverse events in vascular responders and non-responder

There was no difference in grade 3/4 adverse events in cediranib-treated vascular responders versus non-responders (p=0·94).

| **Grade 3/4**  **adverse event** | **Arm 1**  Paclitaxel | **Patients treated with cediranib (Arm 2/3)** | |
| --- | --- | --- | --- |
|  |  | **Vascular response** | **No vascular response** |
| Yes | 9 | 23 | 17 |
| No | 32 | 19 | 13 |

Supplementary Table S16: Circulating tumour cell data.

Key: * Denominator is the number of whole blood samples successfully tested; † CTCs are called when they have the morphology of an intact cell and are CK+/ DAPI+/CD45–; ‡ Denominator is the number of whole blood samples with ≥1 CTCs. Whole blood samples were collected at cycle 1 day 1 on all treatment arms.

|  | **Combined**  **cohort** | **Arm 1**  Paclitaxel | **Arm 2**  Paclitaxel/cediranib | **Arm 3**  Olaparib/cediranib |
| --- | --- | --- | --- | --- |
| **CTC analysis – no. (%)** * |  |  |  |  |
| Whole blood samples donated | 105 | 37 | 33 | 36 |
| Whole blood samples successfully tested | 93 | 34 | 28 | 31 |
| CTCs per 7.5 mL of whole blood † |  |  |  |  |
| ≥1 CTCs | 29 (31%) | 12 (35%) | 6 (21%) | 11 (35%) |
| ≥5 CTCs | 7 (8%) | 2 (6%) | 3 (11%) | 2 (6% |
| ≥10 CTCs | 5 (5%) | 1 (3%) | 2 (7%) | 2 (6%) |
| Range of CTCs | 1–53 | 1–17 | 1–53 | 1–17 |
| **Histology (≥1 CTCs) – no. (%)** ‡ |  |  |  |  |
| Carcinoma | 24 (83%) | 8 (67%) | 5 (83%) | 11 (100%) |
| Endometrioid | 15 | 5 | 4 | 6 |
| Grade 1 | 1 | 0 | 0 | 1 |
| Grade 2 | 4 | 1 | 1 | 2 |
| Grade 3 | 10 | 4 | 3 | 3 |
| Serous | 7 | 1 | 1 | 5 |
| Clear cell | 2 | 2 | 0 | 0 |
| Carcinosarcoma | 5 (17%) | 4 (33%) | 1 (17%) | 0 |
| **Prior lines of chemotherapy – no. (%)** |  |  |  |  |
| 0 | 5 (17%) | 0 | 2 (33%) | 3 (27%) |
| ≥1 | 24 (83%) | 12 (100%) | 4 (67%) | 8 (83%) |

## Supplementary Figure S7. Kaplan-Meier curves for progression-free survival according to CTC status

93 patients were successfully tested for CTCs; 64 had no CTCs (blue line ‘without CTC’) and 29 had ≥1 CTCs (red line ‘with CTC’). The median PFS for patients with and without CTCs were 5·0 months (95% CI 1·5–7·7) and 7·2 months (95% CI 5·4–8·9), respectively (p=0·0014).


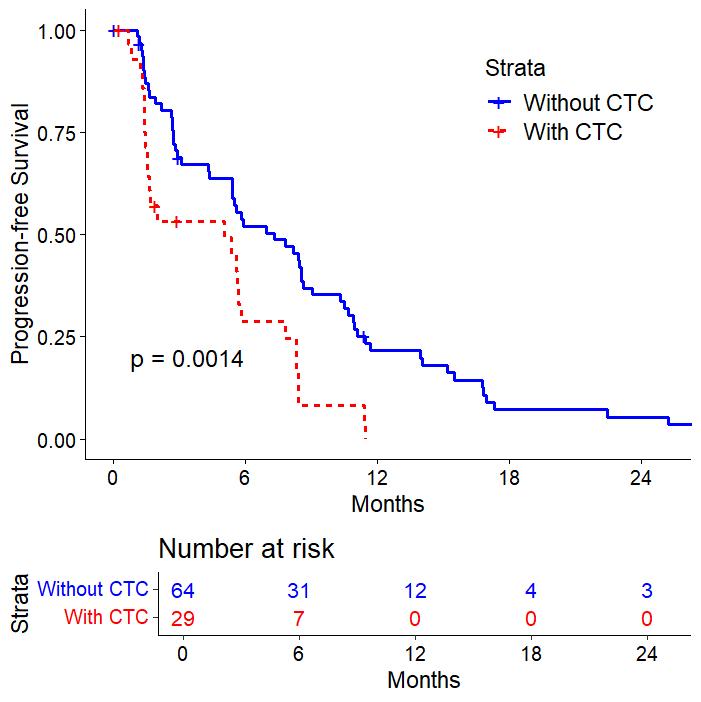


## Supplementary Figure S8. Kaplan-Meier curves for overall survival according to CTC status

93 patients were successfully tested for CTCs; 64 had no CTCs (blue line ‘without CTC’) and 29 had ≥1 CTCs (red line ‘with CTC’). The median OS for patients with and without CTCs was 11·2 months (95% CI 6·2–17·9) and 14·5 months (95% CI 11·5–20·4), respectively (p=0·017).


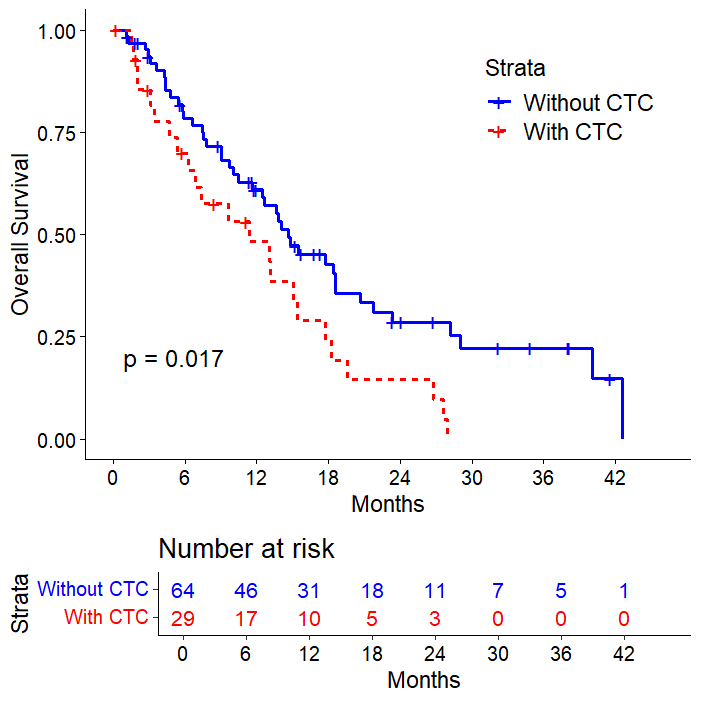


## Supplementary Table S17. Multivariable Cox proportional hazards analysis for circulating tumour cells

Key: HR, hazard ratio; 95% CI, 95% confidence interval; dMMR, mismatch repair deficient; p53abn, abnormal p53 expression. Univariable analysis found histological grade (PFS, OS), molecular subtype (PFS) and prior radiotherapy (PFS, OS) were prognostic in the overall population (123 patients) (Supplementary Table S13).

| **Variable** | **PFS** | |
| --- | --- | --- |
|  | **HR (95%CI)** | **P value** |
| **CTCs detected at cycle 1 day 1 (pre-treatment)**  ≥1 CTCs versus no CTCs | 2·13 (1·27–3·59) | 0·004 |
| **Histological grade**  2 versus 1  3 versus 1 | 2·37 (1·25–10·47)  2·54 (1·31–8·16) | 0·018  0·011 |
| **Molecular subtype**  p53abn versus dMMR  NSMP versus dMMR  Unknown versus dMMR | 0·39 (0·10–0·53)  0·36 (0·16–0·83)  0·60 (0·17–0·85) | 0·001  0·016  0·18 |
| **Prior radiotherapy**  No versus yes | 1·64 (0·99–2·73) | 0·054 |

| **Variable** | **OS** | |
| --- | --- | --- |
|  | **HR (95%CI)** | **P value** |
| **CTCs detected at cycle 1 day 1 (pre-treatment)**  ≥1 CTCs versus no CTCs | 1·75 (1·04–2·94) | 0·036 |
| **Histological grade**  2 versus 1  3 versus 1 | 3·00 (2·02–28·36)  2·06 (1·06–11·34) | 0·003  0·039 |
| **Prior radiotherapy**  No versus yes | 1·61 (0·96–2·71) | 0·07 |
