# Supplementary material for: Cediranib with weekly paclitaxel or olaparib versus weekly paclitaxel for advanced or recurrent endometrial cancer (COPELIA): a multicentre, open-label, randomised, phase 2 trial in the UK
Source: eClinicalMedicine. 2026 Jun 16;96:104012. doi: 10.1016/j.eclinm.2026.104012 (PMC13284432; doi:10.1016/j.eclinm.2026.104012)
Supplement: Trial Protocol [file mmc2.docx]

COPELIA

A 3-Arm Randomised Phase II Evaluation of Cediranib in Combination with Weekly Paclitaxel or Olaparib Versus Weekly Paclitaxel Chemotherapy for Advanced Endometrial Carcinoma or for disease relapse within 18 months of adjuvant carboplatin-paclitaxel chemotherapy.

V7.0 (22AUG2023)

| Sponsor: | University of Manchester |
| --- | --- |
| Sponsor ref: | R120537 |
| Funder: | AstraZeneca |
| Funder ref: | ESR-15-11357 |
| REC ref: | 17/SC/0536 |
| IRAS number: | 216069 |
| EudraCT ref: | 2016-004617-28 |
| WHO UT number: | U1111-1197-9991 |
| ISRCTN/ ClinicalTrials.gov ref: | ISRCTN16320634 |
| Q-Pulse Document Template Number: | TPL/003/001 v1.0 |

# Table of Contents

[Table of Contents 2](#_Toc228557432)

[Glossary of abbreviations 7](#_Toc228557433)

[Synopsis 9](#_Toc228557434)

[1 Trial summary & schema 13](#_Toc228557435)

[1.1 Participant flow diagram 13](#_Toc228557436)

[1.2 Overview of assessments during trial 14](#_Toc228557437)

[1.3 Trial lay summary: Does cediranib together with paclitaxel chemotherapy, or cediranib and olaparib, treat advanced endometrial cancer better than paclitaxel chemotherapy? 15](#_Toc228557438)

[2 Background 16](#_Toc228557439)

[2.1 Rationale for current trial/Justification of treatment options 18](#_Toc228557440)

[2.1.1 Rationale for evaluation of VEGF inhibitors in recurrent endometrial cancer 18](#_Toc228557441)

[2.1.2 Rationale for evaluation of cediranib in gynaecological cancers 19](#_Toc228557442)

[2.1.3 Rationale for weekly paclitaxel and VEGF inhibitors in recurrent endometrial cancer 20](#_Toc228557443)

[2.1.4 Rationale for evaluation of olaparib (a PARP inhibitor) in recurrent endometrial cancer 20](#_Toc228557444)

[2.1.5 Rationale for evaluation of combination therapy of cediranib and olaparib in recurrent endometrial cancer 21](#_Toc228557445)

[3 Trial objectives/endpoints and outcome measures 23](#_Toc228557446)

[3.1 Primary objectives 23](#_Toc228557447)

[3.2 Secondary objectives 24](#_Toc228557448)

[3.3 Exploratory/Translational objectives(s) 24](#_Toc228557449)

[3.4 Primary outcomes measure(s) 24](#_Toc228557450)

[3.5 Secondary outcomes measure(s) 25](#_Toc228557451)

[3.6 Exploratory/Translational outcomes measure(s) 25](#_Toc228557452)

[4 Trial design and setting 25](#_Toc228557453)

[4.1 Risk assessment 27](#_Toc228557454)

[5 Site and Investigator selection 27](#_Toc228557455)

[6 Participant selection 29](#_Toc228557456)

[6.1 Inclusion criteria 29](#_Toc228557457)

[6.2 Exclusion criteria 30](#_Toc228557458)

[7 Screening, Registration and Recruitment 33](#_Toc228557459)

[7.1 Participant identification 33](#_Toc228557460)

[7.2 Screening logs 33](#_Toc228557461)

[7.3 Recruitment rates 33](#_Toc228557462)

[7.4 Informed consent 33](#_Toc228557463)

[7.5 Registration and Randomisation 34](#_Toc228557464)

[7.5.1 Registration 34](#_Toc228557465)

[7.5.2 Randomisation 35](#_Toc228557466)

[8 Withdrawal & lost to follow-up 36](#_Toc228557467)

[8.1 Withdrawal 36](#_Toc228557468)

[8.2 Lost to follow up 38](#_Toc228557469)

[9 Trial Intervention 39](#_Toc228557470)

[9.1 Treatment(s) 39](#_Toc228557471)

[9.1.1 Paclitaxel 39](#_Toc228557472)

[9.1.2 Cediranib 39](#_Toc228557473)

[9.1.3 Olaparib 40](#_Toc228557474)

[9.2 Treatment supply and storage 41](#_Toc228557475)

[9.2.1 Supply 41](#_Toc228557476)

[9.2.2 Storage 43](#_Toc228557477)

[9.2.3 Destruction 44](#_Toc228557478)

[9.3 Treatment prescribing and dispensing 44](#_Toc228557479)

[9.4 Dosing schedule 45](#_Toc228557480)

[9.4.1 Dosing schedule: Paclitaxel 45](#_Toc228557481)

[9.4.2 Dosing schedule: Cediranib 47](#_Toc228557482)

[9.4.3 Dosing schedule: Olaparib 48](#_Toc228557483)

[9.5 Dose modifications for toxicity 49](#_Toc228557484)

[9.5.1 Paclitaxel dose modifications 50](#_Toc228557485)

[9.5.2 Cediranib dose modifications 52](#_Toc228557486)

[9.5.3 Olaparib dose modifications 58](#_Toc228557487)

[9.6 Pre-medication 64](#_Toc228557488)

[9.7 Management of an overdose 64](#_Toc228557489)

[9.7.1 Overdose of cediranib 65](#_Toc228557490)

[9.7.2 Overdose of olaparib 65](#_Toc228557491)

[9.8 Prohibited medications and interaction with other drugs 65](#_Toc228557492)

[9.8.1 Cediranib-restricted concomitant medications 65](#_Toc228557493)

[9.8.2 Olaparib-restricted concomitant medications 65](#_Toc228557494)

[9.9 Permitted concomitant medications 67](#_Toc228557495)

[9.10 Trial restrictions 67](#_Toc228557496)

[9.10.1 Special warning and precautions/ restrictions during the study for olaparib: 67](#_Toc228557497)

[9.10.2 Special warnings and precautions for cediranib: 68](#_Toc228557498)

[9.10.3 General warnings and precautions for all treatment arms: 68](#_Toc228557499)

[9.11 Accountability procedures 70](#_Toc228557500)

[9.12 Compliance 70](#_Toc228557501)

[10 Sample Management 71](#_Toc228557502)

[10.1 Samples for measurement of angiomodulatory molecules in plasma 73](#_Toc228557503)

[10.2 Whole blood samples for evaluation of circulating tumour cells (CTCs) and future use 74](#_Toc228557504)

[10.3 Tumour samples for evaluation of *POLEm*, MMRd, p53abn and NSMP 74](#_Toc228557505)

[Trial visits and procedures 74](#_Toc228557506)

[10.4 Screening for eligibility and registration to the trial 75](#_Toc228557507)

[10.5 Randomisation 76](#_Toc228557508)

[10.6 Treatment associated visits for up to six cycles 77](#_Toc228557509)

[10.7 CT scans 77](#_Toc228557510)

[10.8 End-of-treatment assessment 77](#_Toc228557511)

[10.9 Monthly assessments in hospital until disease progression 78](#_Toc228557512)

[10.10 Remote follow-up: 3-monthly 78](#_Toc228557513)

[10.11 Schedule of Assessments 78](#_Toc228557514)

[10.12 Follow-up 83](#_Toc228557515)

[11 Pharmacovigilance 83](#_Toc228557516)

[11.1 Definitions 83](#_Toc228557517)

[11.2 Trial Specific SAE Reporting requirements 84](#_Toc228557518)

[11.3 Causality 85](#_Toc228557519)

[11.4 Expectedness 86](#_Toc228557520)

[11.5 Reporting procedures 87](#_Toc228557521)

[11.5.1 Participating site responsibilities 87](#_Toc228557522)

[11.5.2 CTR responsibilities 88](#_Toc228557523)

[11.6 SUSAR reporting 89](#_Toc228557524)

[11.7 Unblinding for the purposes of SUSAR reporting 89](#_Toc228557525)

[11.8 Safety Reports 89](#_Toc228557526)

[11.9 Contraception and pregnancy 90](#_Toc228557527)

[11.9.1 Contraception 90](#_Toc228557528)

[11.9.2 Pregnancy reporting whilst participating in the trial 91](#_Toc228557529)

[11.10 Urgent Safety Measures (USMs) 91](#_Toc228557530)

[12 Statistical considerations 91](#_Toc228557531)

[12.1 Randomisation 91](#_Toc228557532)

[12.2 Blinding 91](#_Toc228557533)

[12.3 Sample size 91](#_Toc228557534)

[12.4 Missing, unused & spurious data 92](#_Toc228557535)

[12.5 Procedures for reporting deviation(s) from the original SAP 92](#_Toc228557536)

[12.6 Termination of the trial 92](#_Toc228557537)

[12.7 Inclusion in analysis 93](#_Toc228557538)

[13 Analysis 93](#_Toc228557539)

[13.1 Main analysis 93](#_Toc228557540)

[13.1.1 Safety Analysis: 93](#_Toc228557541)

[13.1.2 Efficacy Analysis: 94](#_Toc228557542)

[13.1.3 Exploratory Analysis: 94](#_Toc228557543)

[13.1.4 Quality of life Analysis: 95](#_Toc228557544)

[13.1.5 Interim analysis 95](#_Toc228557545)

[14 Data Management 95](#_Toc228557546)

[14.1 Completion of Paper CRFs 96](#_Toc228557547)

[15 Translational research 97](#_Toc228557548)

[16 Protocol/GCP non-compliance 98](#_Toc228557549)

[17 End of Trial definition 99](#_Toc228557550)

[18 Archiving 99](#_Toc228557551)

[19 Regulatory Considerations 99](#_Toc228557552)

[19.1 CTA 99](#_Toc228557553)

[19.2 Ethical and governance approval 99](#_Toc228557554)

[19.3 Data protection and participant confidentiality 100](#_Toc228557555)

[19.3.1 Data at sites 100](#_Toc228557556)

[19.3.2 Data at the CTR 100](#_Toc228557557)

[19.4 Indemnity and Sponsorship 101](#_Toc228557558)

[19.5 Funding 101](#_Toc228557559)

[20 Trial committees 101](#_Toc228557560)

[20.1 TMG (Trial Management Group) 101](#_Toc228557561)

[20.2 TSC (Trial Steering Committee) 102](#_Toc228557562)

[20.3 Independent Data Monitoring Committee (IDMC) 102](#_Toc228557563)

[21 Quality Control and Assurance 103](#_Toc228557564)

[21.1 Monitoring 103](#_Toc228557565)

[21.2 Audits & inspections 103](#_Toc228557566)

[22 Publication policy 104](#_Toc228557567)

[23 References 104](#_Toc228557568)

[24 Appendices 111](#_Toc228557569)

[24.1 Appendix 1 111](#_Toc228557570)

[24.2 Appendix 2 112](#_Toc228557571)

# Glossary of abbreviations

| ADL | Activities of Daily Living |
| --- | --- |
| AE | Adverse Event |
| AR | Adverse Reaction |
| AML | Acute Myeloid Leukaemia |
| ANC | Absolute Neutrophil Count |
| AUC | Area Under the Curve |
| C | Cycle (e.g., C1 is cycle 1) |
| CI | Chief Investigator |
| CRF | Case Report Form |
| CT | Computerised Tomography |
| CTA | Clinical Trials Authorisation |
| CTC(s) | Circulating Tumour Cell(s) |
| CTCAE | National Cancer Institute Common Terminology Criteria for Adverse Events |
| CTR | Centre for Trials Research |
| CTU | Clinical Trials Unit |
| D | Day of the cycle, (e.g., D1 is day 1) |
| DNA | Deoxyribonucleic acid |
| DSUR | Development Safety Update Report |
| ECG | Electrocardiogram |
| ECOG | Eastern Cooperative Oncology Group |
| EudraCT | European Clinical Trials Database |
| GCP | Good Clinical Practice |
| GFR | Glomerular Filtration Rate |
| GI | Gastrointestinal |
| GOG | Gynaecologic Oncology Group |
| Hb | Haemoglobin |
| HR | Hazard ratio |
| HRA | NHS Health Research Authority |
| HRR | Homologous Recombination Repair |
| HTA | Human Tissue Authority |
| IB | Investigator’s Brochure |
| ICF | Informed consent form |
| IDMC | Independent Data Monitoring Committee |
| IMP | Investigational Medicinal Product |
| INR | International normalised ratio |
| ISF | Investigator Site File |
| ISRCTN | International Standard Randomised Controlled Trial Number |
| IV | Intravenous |
| MDS | Myelodysplastic Syndrome |
| MHRA | Medicine and Healthcare products Regulatory Agency |
| MSI | Microsatellite Instability |
| nIMP | non-Investigational Medicinal Product |
| OS | Overall Survival |
| PARP | Poly ADP Ribose Polymerase |
| PD | Progressive disease |
| PDGFR | Platelet Derived Growth Factor Receptor |
| PFS | Progression Free Survival |
| PI | Principal Investigator |
| PIS | Participant Information Sheet |
| PRES | Posterior Reversible Encephalopathy Syndrome |
| PSF | Pharmacy Site File |
| QL | Quality of Life |
| REC | Research Ethics Committee |
| RPLE | Reversible Posterior Leukoencephalopathy syndrome |
| SAE | Serious Adverse Event |
| SAR | Serious Adverse Reaction |
| SOP | Standard Operating Procedure |
| SmPC | Summary of Product Characteristics |
| SUSAR | Suspected Unexpected Serious Adverse Reactions |
| TFT | Thyroid Function Test |
| TM | Trial Manager |
| TMF | Trial Master File |
| TMG | Trial Management Group |
| TSC | Trial Steering Committee |
| ULN | Upper limit of normal |
| VEGF | Vascular Endothelial Growth Factor |
| VEGFR | VEGF receptor |

# Synopsis

| Short title | A 3-Arm Randomised Phase II Evaluation of Cediranib in Combination with Weekly Paclitaxel or Olaparib Versus Weekly Paclitaxel Chemotherapy for Advanced Endometrial Carcinoma or for disease relapse within 18 months of adjuvant carboplatin-paclitaxel chemotherapy | | |
| --- | --- | --- | --- |
| Acronym | COPELIA | | |
| Clinical phase | Phase II | | |
| Sponsor ref. | COPELIA01 | | |
| Funder and ref. | AstraZeneca (Reference: ESR-15-11357) | | |
| Number of Sites | 15 | | |
| Trial design | Randomised, controlled, 3-arm, open-label, parallel group, multi-arm-multi-stage trial.  Participants will be randomised 1:1:1 as follows:   - Arm 1: Paclitaxel at 80 mg/m^2^ IV on days 1, 8 and 15 of a 28-day cycle for 6 cycles. - Arm 2: Paclitaxel at 80 mg/m^2^ IV on days 1, 8 and 15 of a 28-day cycle for 6 cycles with cediranib 20 mg orally once daily continuously in 28-day cycles until disease progression. - Arm 3: Cediranib 20 mg orally once daily and Olaparib 300 mg orally twice daily continuously in 28-day cycles until disease progression.   The multi-arm-multistage design allows one of the experimental arms to be dropped, or the trial to be stopped early, for lack of benefit at a set interim timepoint. | | |
| Trial participants | Patients with advanced inoperable/metastatic/recurrent endometrial carcinoma or carcinosarcoma, recruited from the hospital setting. | | |
| Planned sample size | 129 (n=43 per arm) | | |
| Main inclusion criteria | For full and detailed inclusion criteria see section 8.1.   - Women (aged > 16 years) with histologically confirmed advanced or recurrent endometrial carcinoma or carcinosarcoma. - All participants must have received at least one prior line of platinum-based chemotherapy (either in the adjuvant or recurrent disease setting). In addition, ONE of the following must apply:   1. have disease recurrence/ progression within 18 months of completing adjuvant chemotherapy and have received no cytotoxic chemotherapy for recurrent/ progressive endometrial cancer.   OR   - 1. have received one or two prior lines of cytotoxic chemotherapy for recurrent/ progressive endometrial cancer (not counting adjuvant treatment). For clarity, a patient who has locally advanced (inoperable) or metastatic disease at initial diagnosis and receives carboplatin-paclitaxel chemotherapy as their primary treatment is eligible for COPELIA at disease progression i.e. they do not have to receive a second line of chemotherapy before trial entry. - Dose-dense weekly paclitaxel is an appropriate treatment option. - ECOG Performance Status 0-1. - Life expectancy greater than 16 weeks. - Measurable disease by RECIST v1.1. - Adequate hematological, liver, renal and thyroid function. - Ability to swallow oral medication. | | |
| Main exclusion criteria | For full and detailed exclusion criteria see section 8.2.   - Prior treatment with dose-dense weekly paclitaxel. - Concomitant use of strong or moderate CYP3A inhibitors or inducers. - If side effects of previous treatments have not resolved to grade I or less, with the exception of alopecia. - Radiotherapy, chemotherapy, surgery or tumour embolisation within 28 days before the first dose of IMP. - Additional concurrent anti-cancer therapy. - Inadequately controlled hypertension, defined as ≥150/90 mmHg - Prior or concurrent therapy with a PARP or VEGF inhibitor. - Patients with known hypersensitivity to olaparib, cediranib or paclitaxel or any of the excipients of the products. - Patients with myelodysplastic syndrome (MDS), acute myeloid leukaemia (AML) or with features suggestive of MDS/AML. - Other malignancy within the last 5 years. - Resting ECG with QTc > 470 ms on 2 or more time points within a 24-hour period or family history of long QT syndrome. - Causes of malabsorption e.g., uncontrolled diarrhea or poorly controlled stoma are not permitted. - Bowel obstruction, fistulae, extensive rectosigmoid involvement by cancer are not permitted. | | |
| Treatment duration | Expected duration 6 months. Treatment will end before 6 months if disease progresses. Paclitaxel will not be given beyond 6 months, but oral cediranib and olaparib in Arms 2 or 3 may be given until disease progression. | | |
| Follow-up duration | Follow-up involves hospital visits until disease progression. Beyond disease progression, there will be remote 3-monthly follow-up—with survival data obtained from the participant’s medical notes or by phone call to the participant or their GP. The trial, and all follow-up, will end once all participants have met at least one of the criteria: completed 12 months’ follow-up (including treatment), withdrawn from follow-up, been lost to follow-up, experienced disease progression, or died. | | |
| Planned trial period | 6 months set-up, 1month minimum screening time, 30 months accrual, 6 months treatment time, 12month follow-up (including treatment), 6 months to close down = 55 months. | | |
| Primary objective | To evaluate the therapeutic benefit of two novel combination regimens: cediranib and weekly paclitaxel (Arm 2) and cediranib-olaparib (Arm 3) compared to a widely-accepted standard treatment of weekly paclitaxel (Arm 1) for measurable, advanced, inoperable endometrial cancer where disease recurrence/ progression has occurred within 18 months of receiving platinum-containing adjuvant therapy or after no more than two lines of prior cytotoxic chemotherapy for recurrent disease. | | |
| Secondary objectives | To evaluate the tolerability and safety of cediranib added to weekly paclitaxel and cediranib-olaparib compared to weekly paclitaxel for measurable, advanced, inoperable endometrial cancer where disease recurrence/ progression has occurred within 18 months of receiving platinum-containing adjuvant therapy or after no more than two lines of prior cytotoxic chemotherapy for recurrent disease.  To also evaluate QL using validated patient reported outcome measures during treatment with cediranib and weekly paclitaxel, cediranib-olaparib or weekly paclitaxel. | | |
| Exploratory/Translational research objectives | To evaluate putative predictive and resistance biomarkers in an integrated translational research programme that includes:   1. Measurement of angiomodulatory molecules in plasma. 2. Evaluation of proportion of advanced endometrial cancer patients with detectable CTCs at baseline, and determination of whether dynamic changes in CTC levels correlate with radiological response. 3. Evaluation of gamma H2AX in CTCs. 4. Evaluation of *POLE*m, MMRd, p53abn and NSMP molecular groups. | | |
| Primary outcomes | The primary outcome is the proportion of participants who are disease progression free at three months as determined by RECIST v1.1. This time point was chosen as three months is the predicted median PFS for patients receiving weekly paclitaxel chemotherapy alone. | | |
| Secondary outcomes | 1. The radiological response rate assessed by RECIST v1.1. 2. The median PFS. 3. Six-month PFS rate. 4. The median OS, calculated as median time from enrolment to death with those still alive censored at date last seen. 5. All toxicities associated with each treatment regimen as assessed by CTCAE version 4.03. 6. QL as measured by EORTC QLQ-C30 and EN28. | | |
| Exploratory/Translational research outcomes | 1. Plasma concentration of circulating angiogenesis-related cytokines. 2. The proportion of participants with detectable CTCs at baseline, and the correlation between CTC levels and radiological response. 3. Evaluation of gamma H2AX in CTCs. 4. Evaluation of *POLE*m, MMRd, p53abn and NSMP molecular groups. | | |
| Investigational medicinal products | Olaparib (AZD2281) | Cediranib (AZD2171) | Paclitaxel |
| Form | Tablet | Tablet | Solution for dilution for infusion. |
| Dose | Olaparib 300 mg twice daily continuously in 28 day cycles until disease progression. | Cediranib 20 mg once daily continuously in 28 day cycles until disease progression. | 80 mg/m^2^ IV on days 1, 8 and 15 of a 28-day cycle for 6 cycles. |
| Route | Oral | Oral | IV |

# Trial summary & schema

## Participant flow diagram


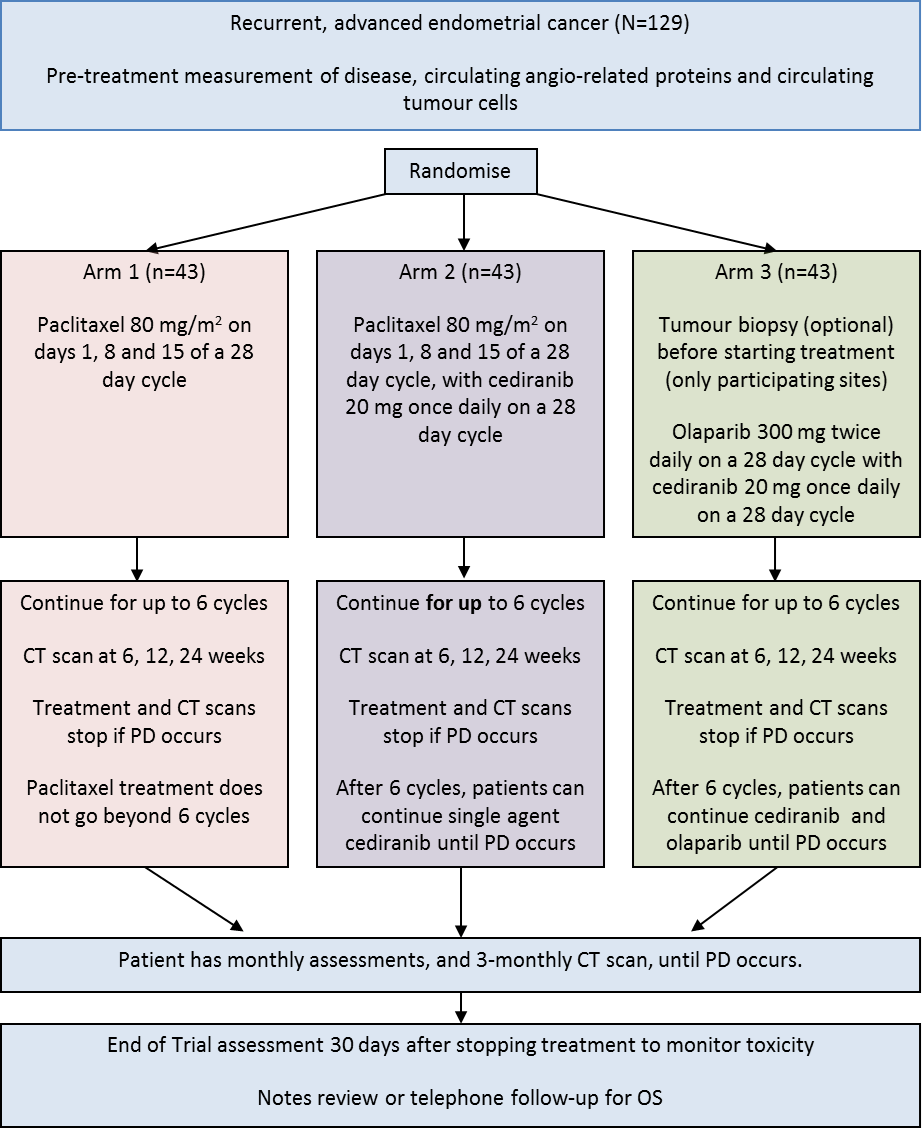

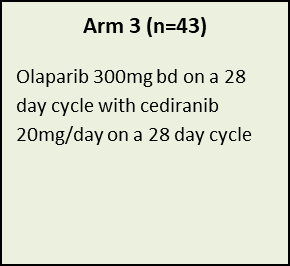


## **Overview of assessments during trial**


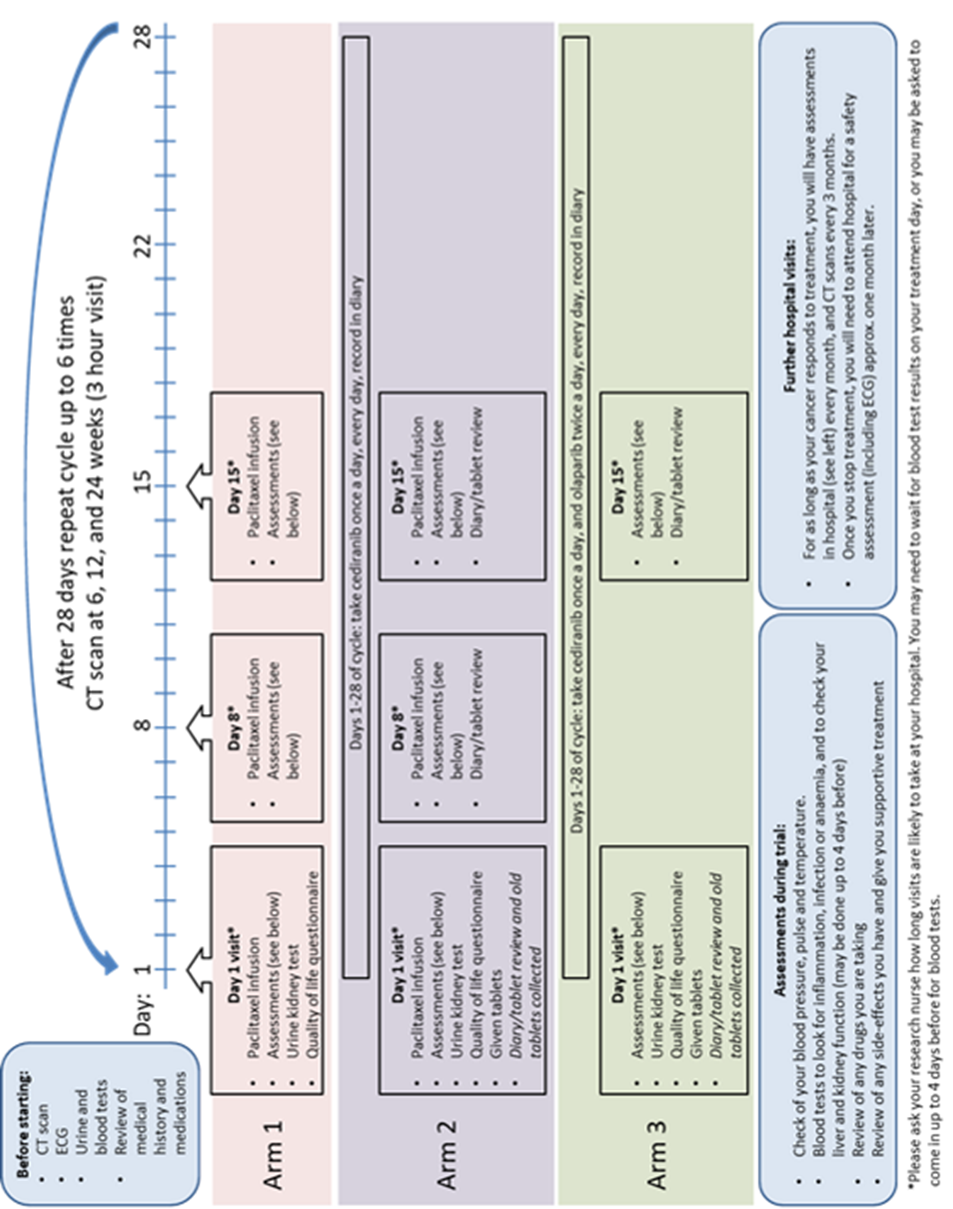


## Trial lay summary: Does cediranib together with paclitaxel chemotherapy, or cediranib and olaparib, treat advanced endometrial cancer better than paclitaxel chemotherapy?

In the UK there has been a 50 % increase over the last 20 years in the number of women developing endometrial cancer (cancer arising from the lining of the womb). Most of these women are cured by surgery to remove the womb and ovaries. However, in some cases, the cancer recurs or is at an advanced stage when it is first diagnosed. In these women, chemotherapy treatment to control the endometrial cancer is often recommended. The benefits from this initial chemotherapy are often limited and more treatment may be required. Currently, there has been little research looking at new drug treatments for recurrent endometrial cancer.

The COPELIA trial is evaluating two new tablet medications in endometrial cancer for the first time. It will include 129 women aged 16 years or older with advanced endometrial cancer whose cancer has worsened after their initial chemotherapy treatment. Participants will be allocated at random to one of three groups:

1. The first group (Arm 1) will receive a standard (routine) treatment for patients with endometrial cancer known as paclitaxel. This is a chemotherapy drug that is routinely used to treat patients with different cancers including ovarian, breast, lung and endometrial cancer. Paclitaxel works by stopping the growth of cancer cells. In this trial, paclitaxel is given via an hour-long infusion into a vein once a week.
2. The second group (Arm 2) will receive the standard paclitaxel treatment once a week in addition to a new drug called cediranib. Cediranib is a tablet medication that is taken once a day and works by blocking new blood vessel formation. Cediranib has been tested in women with endometrial cancer before but not alongside chemotherapy treatment.
3. The third group (Arm 3) will receive two new tablet medications, cediranib and olaparib. Cediranib is taken once a day and olaparib twice daily. Olaparib works by preventing cancer cells repairing DNA effectively. The use of olaparib and cediranib together has been shown to be effective in a common type of ovarian cancer but has not been evaluated as a treatment for endometrial cancer before.

The main objectives of the COPELIA trial are to work out:

1. Whether the two new treatments, cediranib-paclitaxel (Arm 2) and cediranib-olaparib (Arm 3) are more effective at controlling endometrial cancer than standard paclitaxel chemotherapy (Arm 1)
2. Whether the two new treatments cause more or fewer side-effects than standard chemotherapy
3. How each of these treatments impact on the daily life of women receiving the treatment by asking trial participants to regularly complete QL questionnaires
4. Whether we can learn how these treatments work in women with endometrial cancer by taking some additional blood tests for research.

# Background

The incidence of endometrial cancer is rising rapidly. There were 8,475 cases diagnosed in the UK in 2011, a 50 % increase in the last 20 years, and 320,000 cases worldwide. Risk factors for endometrial cancer include increasing age, unopposed or prolonged oestrogen exposure (e.g., early menarche, late menopause, nulliparity, obesity, use of oestrogen replacement therapy) and genetic predisposition (e.g., Lynch syndrome).

Endometrial cancers can be classified into two groups based on clinicopathologic features and oestrogen-dependence. Type I cancers are well-differentiated, low grade cancers of endometrioid histology that are oestrogen-dependent, whilst Type II cancers are usually poorly differentiated, high grade tumours, most commonly of serous or clear cell histology that are oestrogen-independent. They have an aggressive clinical course and are more likely to present with higher stage disease. This classification is of limited utility for predicting prognosis or response to treatment (Salvesen et al 2012, Hecht et al 2006).

Recent integrated genomic analysis led to molecular classification of endometrial cancer with enhanced prognostic value (Cancer Genome Atlas 2013). . Thus, it has become standard practice to stratify endometrial cancers in to one of four distinct molecular groups, by testing tumour tissue for somatic mutations in certain genes and analysing the expression level of certain proteins (Kommoss et al 2018, Leon-Castillo et al 2020). These four molecular groups include tumours with a *POLE* mutation (*POLEm*), mismatch repair deficiency (MMRd), abnormal p53 expression (p53abn) and no specific molecular profile (NSMP) (Kommoss et al 2018, Leon-Castillo et al 2020). To comply with current international standards, archival tissue from patients enrolled on COPELIA will undergo molecular testing to determine the number of tumours with a *POLEm*, MMRd, p53abn and NSMP.

Endometrial (uterine) carcinosarcomas are now recognised as aggressive, dedifferentiated carcinomas that share some clinical and biological characteristics with type II endometrial cancers. Detailed molecular profiling confirms common mutational profiles in the carcinomatous and sarcomatous components of the same tumours reflecting a common origin. Molecular heterogeneity resembles that seen in type II carcinomas, with some carcinosarcomas exhibiting endometrioid-type mutational profiles and other serous-type profiles (McConechy et al 2015). Response rates and clinical efficacy of carboplatin-paclitaxel in recurrent carcinosarcoma are also similar to that seen in type II endometrial carcinomas (Powell et al 2010). They are currently managed using similar clinical pathways. These data support the inclusion of patients with advanced/ recurrent uterine carcinosarcoma in the COPELIA trial.

While many patients are diagnosed with early stage disease (FIGO stage I and II) that is often curable with surgery with or without adjuvant treatment, about 20 % of these patients experience disease relapse, and 25-30 % of women present with FIGO stage III-IV disease. Overall mortality rates have increased by more than 20 % since 1990 and there were almost 2000 deaths from endometrial cancer in the UK in 2011 (Cancer Research UK Cancer Statistics 2014).

Cytotoxic chemotherapy is a key component of the management of advanced/ recurrent endometrial cancer with the carboplatin-paclitaxel doublet now established as the de facto first-line standard-of-care following two key GOG phase III trials. GOG 177 randomised 263 women to doxorubicin/cisplatin (AP) or doxorubicin/cisplatin/paclitaxel (TAP) and found a survival benefit for the triplet combination (Fleming et al 2004). However, TAP was associated with significantly increased toxicity limiting its clinical utility. Subsequently, GOG 209 recruited 1300 patients who were allocated to receive adjuvant therapy for stage III endometrial cancer or with measurable advanced/ recurrent disease and demonstrated approximate equivalence in response rate, PFS and OS (OS; 38 versus 32 months, not significant) when carboplatin and paclitaxel was compared with TAP (Miller et al. 2012). However, in recurrent endometrial cancer, multiple phase II trials have shown that, despite response rates of 50-60 % with carboplatin-paclitaxel, median OS is disappointing (median of 15-18 months).

No defined standard for second-line therapy exists, and phase II evaluations of multiple cytotoxic agents have reported response rates in this setting of approximately 10 % with median PFS and OS of 3 and 10 months respectively (Fleming et al 2015). There is therefore an urgent need to define more effective treatment strategies for recurrent/ progressive endometrial cancer, and to evaluate the activity of targeted agents in this patient group.

In the absence of a defined second-line or later standard treatment, many centres are utilising weekly paclitaxel in this setting as it is well-tolerated, has little negative impact on QL and is supported by phase II data (Homesley et al 2008) documenting a 27 % response rate.

## Rationale for current trial/Justification of treatment options

### Rationale for evaluation of VEGF inhibitors in recurrent endometrial cancer

The endometrium is an angiogenic tissue with VEGF-dependent vascular remodelling occurring physiologically during menstruation. Retrospective series in endometrial cancer have also shown that tumour VEGF expression and microvessel density are independently associated with poor prognosis (Kamat et al 2007).

Phase II evaluations of both bevacizumab and sunitinib have been conducted in recurrent endometrial cancer after 1-2 prior lines of chemotherapy with response rates of 14 % and 18 % respectively (Aghajanian et al 2011; Castonguay et al 2014). Most importantly, six month PFS rates of 40 % and 30 % were reported, equating with substantially longer duration of disease control than seen in previous studies of novel cytotoxic agents. Similar results have been reported more recently with brivanib (Powell et al 2014).

The potential utility of anti-VEGF therapies in combination with chemotherapy has been reinforced by the recent presentation of the phase II MITO-END2 trial (Lorusso et al 2015), which evaluated the addition of bevacizumab to first-line chemotherapy. This randomised 108 women to 3-weekly carboplatin-paclitaxel with or without concurrent and maintenance bevacizumab. Treatment with bevacizumab was associated with improvements in response rate (72 % vs 54 %) and importantly in median PFS with a 4.3 month increase noted (13.0 months vs 8.7 months HR 0.59 p=0.036). On subgroup analysis, there were trends towards increased benefit from bevacizumab in the elderly (over 65 years old), patients with ECOG performance status 1-2, and those with non-endometrioid histotypes. The toxicity spectrum was as anticipated, although 11.5 % of patients treated with bevacizumab experienced a thromboembolic event compared to 0 % in those receiving chemotherapy alone. This incidence is higher than that reported in other disease settings and may reflect the profile of co-morbidities seen in patients with endometrial cancer such as pre-existing vascular disease, diabetes and obesity.

### Rationale for evaluation of cediranib in gynaecological cancers

Cediranib is an oral VEGFR-1, VEGFR-2 and VEGFR-3 tyrosine kinase inhibitor with additional activity against platelet-derived growth factor (PDGF) receptors and c-kit, which has been evaluated in gynaecological malignancies. Promising single agent activity has been documented in recurrent ovarian cancer—two separate phase II trials determined that at 30 mg once daily, cediranib was well-tolerated and active with a response rate of 26 % in platinum-sensitive recurrent disease reported by Hirte et al (2015), and a clinical benefit rate at 16 weeks of 30 % reported by Matulonis et al (2009) in a group of patients with disease of mixed platinum sensitivity.

Subsequently, the ICON6 trial (Ledermann et al 2016), a phase III randomised placebo-controlled evaluation of concurrent and maintenance cediranib on a backbone of platinum doublet chemotherapy for platinum-sensitive recurrent ovarian cancer, demonstrated that cediranib 20 mg once daily can be combined safely with cytotoxic chemotherapy in recurrent gynaecological cancer. A promising efficacy signal was seen, with the addition of cediranib improving median PFS from 8.7 to 11.1 months (HR 0.56 95 % CI- 0.44-0.72) and median OS by 5.3 months (HR 0.77 95 % CI- 0.55-1.07). The most commonly seen adverse events associated with cediranib were hypertension, diarrhoea and fatigue, but the toxicity profile was manageable during chemotherapy and in the subsequent maintenance phase.

The CIRCCa randomised phase II trial has also shown promising activity for cediranib in combination with carboplatin-paclitaxel chemotherapy in recurrent cervical cancer (Symonds et al 2015). The addition of cediranib to chemotherapy increased radiological response rate from 45 % to 64 % and median PFS was extended from 6.7 to 8.1months (HR 0.57).

Cediranib at a dose of 30 mg once daily has also been evaluated as a single agent in endometrial cancer. A single-arm phase II trial in 48 patients with recurrent/persistent disease after 1-2 prior lines of chemotherapy has recently been reported (Bender et al 2015). A response rate of 12.5 % was seen with a 6-month PFS rate of 33 %, this result met the predefined criteria for activity warranting further investigation. Of note, 15 patients completed at least 6 months of therapy. Cediranib was well-tolerated although 29 % of participants discontinued therapy because of an adverse event. As anticipated, the most common grade 3-4 toxicities were hypertension, fatigue and diarrhoea. Three patients had pulmonary emboli, but no arterial thromboembolic events were reported.

### Rationale for weekly paclitaxel and VEGF inhibitors in recurrent endometrial cancer

Weekly paclitaxel is a promising treatment strategy that has been adopted as a standard treatment option for many epithelial malignancies, including ovarian and more recently endometrial cancer, because of favourable activity and excellent tolerability.

In platinum-resistant ovarian cancer, phase II trials have shown that weekly administration is better tolerated than 3-weekly dosing and that it is active—with a 25 % response rate documented in patients with disease resistant to conventional 3-weekly platinum-paclitaxel treatment (Rosenberg et al 2002). This differential activity suggests either an improved pharmacokinetic profile or a differential mode of action. Notably, pre-clinical evaluations have demonstrated that metronomic taxane administration may have anti-angiogenic activity and, importantly, synergistic activity with VEGF inhibitors (Aparna et al 2007). Data from the phase III AURELIA trial in platinum-resistant ovarian cancer now provide clinical validity for the combination of an anti-VEGF therapy with weekly paclitaxel (Pujade-Lauraine et al 2012; 2014). In this trial, the addition of bevacizumab to weekly paclitaxel increased both response rate and PFS substantially (PFS HR 0.46; median PFS 3.9 to 10.4 months) with documented improvement in QL parameters (Stockler et al 2014).

This synergistic activity is likely to be also seen with oral VEGFR tyrosine kinase inhibitors. Pazopanib has recently been evaluated in combination with weekly paclitaxel in platinum-resistant ovarian cancer in a randomised phase II trial. There was no concerning toxicity signal and encouraging efficacy with an improvement in median PFS from 3.5 to 6.3 months (HR 0.45; Pignata et al 2015). Together these reports provide a strong rationale and support the feasibility and potential efficacy for evaluating the combination of weekly paclitaxel and cediranib in advanced/ recurrent endometrial cancer.

### Rationale for evaluation of olaparib (a PARP inhibitor) in recurrent endometrial cancer

Many cancers have dysregulated DNA repair pathways that can be exploited as a target for novel drug therapy as they have increased sensitivity to DNA-damaging anticancer therapies (Curtin 2012). One of the DNA repair pathways in tumours that can be therapeutically targeted is homologous recombination. Defective homologous recombination caused by different mechanisms, including loss of BRCA function (genetic and epigenetic), can sensitise cells to PARP inhibitors (PARPi) as a result of synthetic lethality (Mukhopadhyay et al 2012, Daemen et al 2012, Dedes et al 2012, Mendes-Pereira et al 2009, Vilar et al 2011, Ying et al 2012, Drew et al 2011, Moskwa et al 2011). Clinical proof-of-principle has been established in BRCA-mutated breast and ovarian cancers (Audeh et al 2010, Tutt et al 2010, Ledermann et al 2014) but is not yet confirmed for other potentially sensitising lesions despite sporadic cancers showing response. Functional loss of PTEN or MRE11 has been implicated as sensitising to PARPi (Dedes et al 2010; Mendes-Pereira et al 2009; Koppensteiner et al 2014, Vilar et al 2011, Ying et al 2012). Both of these molecular defects are relatively common in endometrial cancer with an estimated incidence of approximately 50 % (Hecht et al 2006, Salvesen et al 2012).

Several groups have reported an association between microsatellite instability (MSI) and components of the DNA double strand break repair pathway including PTEN and MRE11. The presence of MSI has also been linked to susceptibility to PARP inhibition (Bryant et al 2005). MSI is present in approximately 30-40 % of endometrial cancers (Hecht et al 2006, Salvesen et al 2012) as assessed by immunohistochemistry that is well-validated and used in routine clinical practice. The frequency of MSI, as well as MRE11 and PTEN loss-of-function (coupled with their potential for predicting response to PARP inhibitors), in endometrial cancer justifies evaluation of olaparib in this patient population. This is further supported by published data that describe 25 % of endometrial cancer cell lines as manifesting marked sensitivity to olaparib *in vitro* (Miyasaka et al 2014). Olaparib has also shown dramatic clinical activity in a patient with recurrent endometrial cancer (Forster et al 2011).

### Rationale for evaluation of combination therapy of cediranib and olaparib in recurrent endometrial cancer

A combined oral regimen using a VEGFR inhibitor (such as cediranib) and PARP inhibitor (such as olaparib) is a logical strategy in cancers such as endometrial cancer where there is biological and clinical evidence to support the activity of both approaches independently. A phase I trial demonstrated that cediranib and olaparib in capsule formulation can be given safely in combination with manageable toxicities and meaningful activity in recurrent ovarian and breast cancer (Liu et al 2013). Liu et al (2013) defined a recommended phase II dose of cediranib 30 mg once daily and olaparib capsules 200 mg twice daily.

This combination was subsequently compared to olaparib alone (400 mg capsules twice daily) in a randomised phase II trial in 90 women with platinum-sensitive recurrent high-grade ovarian cancer (Liu et al 2014). Cediranib-olaparib demonstrated high levels of activity compared to olaparib alone. Response rates were 80 % and 48 %, respectively, while median PFS was 17.7 months with combination therapy and 9.0 months with olaparib alone (HR 0.42 p=0.005). Interestingly, a post-hoc exploratory analysis suggested that the greatest benefit of combination therapy compared to olaparib alone was seen in patients with wild-type or unknown BRCA status (HR 0.32 compared to 0.55 in patients with a germline BRCA mutation). This potential signal of synergy in patients with alternative markers of PARP inhibitor sensitivity is intriguing and suggests that this oral combination is a relevant novel therapeutic approach to evaluate in recurrent endometrial cancer where PARP inhibitor sensitivity may be due to functional loss of PTEN or MRE11 (Hecht et al 2006, Salvesen et al 2012). This supports the use of cediranib-olaparib as an experimental arm in COPELIA, rather than olaparib as a single agent.

The cediranib-olaparib combination was more toxic than olaparib alone, with a higher incidence of grade 3-4 adverse events reported. The most common grade 3-4 events were fatigue (27 % vs 0 %), diarrhoea (23 % vs 0 %) and hypertension (41 % vs 0 %). Cediranib dose reductions to 20 mg once daily, and then to 15 mg once daily, were allowed, and seventy-seven percent of patients receiving combination therapy required at least one dose reduction of cediranib (Liu et al 2014). In contrast, only twenty-four percent of patients in the olaparib monotherapy group (receiving 400 mg twice daily) required a dose reduction, and twenty-three percent of patients in the combination group required an olaparib dose reduction.

The olaparib capsule formulation (50mg size 0 capsules) imposes a substantial pill burden on women who must take 16 capsules daily at the 400mg twice daily monotherapy dose. Because of this, a tablet formulation of olaparib has been developed. Using an adaptive phase I clinical trial design, it has been shown that following multiple dosing, steady state exposure with olaparib tablets at ≥300 mg twice daily matched that for capsules at 400 mg twice daily, with similar clinical efficacy and toxicity, thus reducing the pill burden to four 150 mg tablets per day (Mateo et al 2016).

The recommended monotherapy tablet dose of 300 mg twice daily has been evaluated as maintenance therapy in BRCA-mutation associated platinum-sensitive ovarian cancer in a placebo-controlled randomised phase 3 trial (SOLO-2, Pujade-Lauraine et al 2017) where it demonstrated similar efficacy to that reported for the capsule formulation in the initial seminal randomised phase II trial which resulted in EMEA approval for olaparib (Ledermann et al 2012).

Subsequently, a further phase I trial has confirmed that the all tablet cediranib-olaparib combination was associated with toxicities consistent with those observed when olaparib capsules were given in combination with cediranib (Liu et al 2015). This study established two acceptable options for phase II dosing, either cediranib 20 mg once daily and olaparib 300 mg twice daily, or cediranib 30 mg once daily and olaparib 200 mg twice daily. Based on the earlier combination study showing that a high proportion of cediranib dose reductions were required from a starting dose of 30 mg once daily (Liu et al 2014), participants randomised to receive cediranib in COPELIA (both experimental arms) will receive cediranib 20 mg once daily. Consequently, in accordance with the combination dose recommendations of Liu et al (2015), participants randomised to receive olaparib (Arm 3) will receive olaparib tablets 300 mg twice daily. Intensive symptom management guidelines will also be applied and QL assessments are incorporated within this trial.

The COPELIA trial is a randomised phase II trial that will evaluate whether the addition of cediranib to weekly paclitaxel, or the use of the all oral non-cytotoxic cediranib-olaparib combination, will improve PFS compared with the widely accepted treatment option, weekly paclitaxel, in the treatment of measurable advanced, recurrent endometrial cancer after prior platinum-based chemotherapy. Key secondary endpoints will be the tolerability of each experimental regimen, their effect on QL measures and an exploratory evaluation of a selected biomarker panel.

# Trial objectives/endpoints and outcome measures

## Primary objectives

1. To evaluate the therapeutic benefit of two novel combination regimens: cediranib and weekly paclitaxel (Arm 2) and cediranib-olaparib (Arm 3) compared to a widely-accepted standard treatment of weekly paclitaxel (Arm 1) for measurable, advanced, inoperable endometrial cancer where disease recurrence/ progression has occurred within 18 months of receiving platinum-containing adjuvant therapy or after no more than two lines of prior cytotoxic chemotherapy for recurrent disease.

The null hypothesis is that there will be no difference in the percentage of participants who are progression-free at the three month time-point.

The alternative hypothesis is that one or both of the experimental trial arms will demonstrate at least a 20 % increase in the proportion of participants who are progression-free at three months compared with participants in the control arm.

This is a multi-arm trial with two experimental arms and one control. Each experimental arm will be compared to control; the two experimental arms will not be compared to each other.

## Secondary objectives

1. To evaluate the tolerability and safety of cediranib added to weekly paclitaxel and cediranib-olaparib compared to weekly paclitaxel in advanced, inoperable endometrial cancer where disease recurrence/ progression has occurred within 18 months of receiving platinum-containing adjuvant therapy or after no more than two lines of prior cytotoxic chemotherapy for recurrent disease.
2. To evaluate QL using validated questionnaires during treatment with cediranib-weekly paclitaxel, cediranib-olaparib or weekly paclitaxel in advanced, inoperable endometrial cancer where disease recurrence/ progression has occurred within 18 months of receiving platinum-containing adjuvant therapy or after no more than two lines of prior cytotoxic chemotherapy for recurrent disease.

## Exploratory/Translational objectives(s)

To evaluate putative predictive and resistance biomarkers in an integrated translational research programme that includes:

- 1. Measurement of angiomodulatory molecules in plasma.
  2. Evaluation of proportion of advanced endometrial cancer patients with detectable CTCs at baseline, and determination of whether dynamic changes in CTC levels correlate with radiological response.
  3. Evaluation of gamma H2AX in CTCs.
  4. Evaluation of *POLEm*, MMRd, p53abn and NSMP molecular groups.

## Primary outcomes measure(s)

1. The primary outcome measure is PFS rate at three months. This is defined as the proportion of participants free from investigator assessed objective disease progression by RECIST v1.1, or death from any cause, three months from the date of randomisation. Measurable disease and non-measurable disease visible on the baseline CT scan will be identified and described by radiologists according to strict RECIST v1.1 criteria. The CT scan will be repeated at six- and twelve-weeks’ post-randomisation and the changes assessed against the baseline scan. In each trial arm, the proportion of participants who are alive and do not meet the RECIST v1.1 criteria for disease progression will be calculated out of all participants allocated to that arm.

## Secondary outcomes measure(s)

1. Radiological response rate assessed by RECIST v1.1.
2. Median PFS where PFS is measured as the time from date of randomisation to date of investigator-assessed objective progression via RECIST v1.1 or death from any cause in the absence of progression.
3. Six-month PFS rate, defined as the proportion of participants free from investigator assessed objective disease progression by RECIST v1.1, or death from any cause, six months from the date of randomisation.
4. Toxicities of any grade associated with each regimen as assessed by CTCAE version 4.03.
5. Median OS defined as the time from date of randomisation to date of death.
6. QL measured using the EORTC QLQ-C30 tool and EN24 endometrial cancer-specific module.

## Exploratory/Translational outcomes measure(s)

1. Plasma concentration of circulating angiogenesis-related cytokines.
2. The proportion of participants with detectable CTCs at baseline, and the correlation between CTC levels and radiological response.
3. Evaluation of gamma H2AX in CTCs will be used as an exploratory tool to correlate presence or absence with radiological response.
4. Evaluation of *POLE*m, MMRd, p53abn and NSMP molecular groups.

# Trial design and setting

COPELIA is a phase II, randomised, three-arm open-label trial which will recruit 129 women aged 16 years or older, with advanced, inoperable endometrial cancer who require further treatment for recurrent/ progressive disease within 18 months of platinum-containing adjuvant chemotherapy or after no more than two prior lines of cytotoxic chemotherapy for recurrent disease. Potential participants will be recruited from the hospital setting. For information on the risk assessment, see section 6.1.

Participants will be allocated to one of the following three trial arms on a 1:1:1 basis using centralised internet randomisation, stratified for prognostic factors:

**Arm 1:** (Control Arm): Paclitaxel 80 mg/m^2^ administered on days 1, 8 and 15 of a 28-day cycle for up to 6 cycles.

**Arm 2**: Cediranib 20 mg once daily for 28 days given with weekly paclitaxel 80 mg/m^2^ administered on days 1, 8 and 15 of a 28-day cycle for up to 6 cycles. Participants with stable disease, partial response or complete response at 6 months as determined by RECIST v1.1 (see Appendix 2) will be eligible to continue treatment with single agent cediranib once daily until disease progression.

**Arm 3**: Cediranib 20 mg once daily with olaparib 300 mg twice daily, continuously on a 28 day cycle for up to 6 cycles. Participants with stable disease, partial response or complete response at 6 months as determined by RECIST v1.1 (see Appendix 2) will be eligible to continue treatment with both olaparib and cediranib until disease progression.

Randomisation will be balanced for the following factors:

1. Histological subtype
   1. Grade (I, II or III) endometrial cancer (serous or clear cell)
   2. Carcinosarcoma of the uterus
2. Number of prior cytotoxic chemotherapy regimens, not including adjuvant treatment.

Recruitment to the trial is expected to take 30 months. The total duration of the trial is expected to be 55 months. Participants will receive initial trial treatments for up to six months as long as they remain progression free. As mentioned above, participants in the experimental arms may continue treatment with cediranib/olaparib beyond the six months, and beyond the end of the trial, if they remain progression free. The trial will end once all participants have met at least one of the criteria: completed 12 months’ follow-up (including treatment), withdrawn from follow-up, been lost to follow-up, experienced disease progression, or died. Participants consent for their medical notes to be reviewed at later dates if required to obtain data such as OS.

The trial has a multi-arm-multi-stage (MAMS) design. The appropriate sample size was calculated for this MAMS design, which allows one (or both) ineffective experimental arms to be dropped following a planned interim analysis after 60 participants have been assessed at three months (see section 15.3).

Data will be collected on paper-based CRFs and submitted to the trial unit through the postal system (see section 17.1). Translational samples will be collected as per section 12.

## Risk assessment

A trial risk assessment has been completed to identify the potential hazards associated with the trial and to assess the likelihood of those hazards occurring and resulting in harm. This risk assessment has been completed in accordance with the MRC/DH/MHRA Joint project guidance document ‘Risk-adapted approaches to the management of Clinical Trials of Investigational Medicinal Products’ and includes:

- The known and potential risks and benefits to human subjects
- An evaluation of how high the risk is compared to normal standard practice
- How the risk will be minimised/managed

This trial has been categorised as Risk Type C where the level of risk is markedly higher than the risk of standard medical care. A copy of the trial risk assessment may be requested from the Trial Manager (TM). The trial risk assessment is used to determine the intensity and focus of monitoring activity (see section 24.1).

# Site and Investigator selection

This trial will be carried out at participating sites within the UK. All sites who are interested in participating in the trial will be required to complete a registration form to confirm that they have adequate resources and experience to conduct the trial. The TM will liaise with sites to ensure the site is opened in accordance with the CTR procedures for site activation.

Potential participants will be identified and recruited at the participating hospital sites. A maximum of 15 cancer centres will participate in the trial. Each participating site will:

- Have an identified PI
- Be provided with protocol specific training before being activated for recruitment
- Be provided with a local document package in line with HRA guidance (http://www.hra.nhs.uk/resources/hra-approval-nhs-organisation-guidance/). For sites in England this package will be provided simultaneously to both the study delivery team and the research management team. For sites in Scotland and Wales the package will be provided as required by the devolved administrations.
- Be provided with copies of the REC, HRA and competent authority approvals for the trial. The approval process includes granting favourable opinion of the host care organisation/PI.
- Have local Trust R&D approval by confirmation of site capability and capacity to undertake the study.
- Execute a signed trial site agreement and other required agreements. A material transfer agreement will be required unless the material transfer was integrated into the site agreement.
- Have a current Curriculum Vitae and GCP training certificate of the PI.
- Complete a Site Delegation Log and Roles and Responsibilities document. It is the responsibility of the PI to ensure only trained and appropriately delegated staff work on the trial.
- Be provided with an ISF which will be maintained and stored securely.
- Be provided with a PSF which will be maintained and stored securely in Pharmacy.
- Be provided with Participant Packs containing paper CRFs which will be stored securely.
- Provide full contact details for all host care organisation personnel involved, indicating the preferred contact.
- Provide a set of laboratory normal ranges and laboratory certification/accreditation from the host care organisation laboratory being used for analyses.
- Return a copy of the Self-Evident Correction Log signed by the PI.
- Provide Pharmacy confirmation that they have received the first shipment of IMP prior to opening the site.

Site initiation will be by attendance at a national COPELIA launch meeting, or by teleconference or a meeting at site if attendance of key personnel at a launch meeting is unfeasible.

Once a site is ready to open for recruitment this will be confirmed in writing to them via an email of authorisation sent by the TM.

# Participant selection

Participants are eligible for the trial if they meet all of the following inclusion criteria and none of the exclusion criteria apply. The eligibility decision for each participant will be made by a medically qualified doctor and documented in the CRF and medical notes. All queries about participant eligibility should be directed to the Trial Manager before registration. Protocol waivers are not permitted.

## Inclusion criteria

1. Histologically confirmed advanced or recurrent endometrial carcinoma or carcinosarcoma.
2. Aged >16 years.
3. All participants must have received at least one prior line of platinum-based chemotherapy (either in the adjuvant or recurrent disease setting). In addition, ONE of the following must apply:
   1. have disease recurrence/ progression within 18 months of completing adjuvant chemotherapy and have received no cytotoxic chemotherapy for recurrent/ progressive endometrial cancer.

OR

- 1. have received one or two prior lines of cytotoxic chemotherapy for recurrent/ progressive endometrial cancer (not counting adjuvant treatment). For clarity, a patient who has locally advanced (inoperable) or metastatic disease at initial diagnosis and receives carboplatin-paclitaxel chemotherapy as their primary treatment is eligible for COPELIA at disease progression i.e. they do not have to receive a second line of chemotherapy before trial entry.

1. Dose-dense weekly paclitaxel is an appropriate treatment option.
2. Ability to provide written informed consent that includes genetic research on tissue derived from biopsies and biomarker research. (If a participant declines to participate in optional exploratory genetic research or the optional biomarker research, there will be no penalty or loss of benefit to the participant. The participant will not be excluded from other aspects of the study).
3. Willing and able to comply with the trial visits and undergo treatment as scheduled.
4. ECOG Performance Status 0-1.
5. Life expectancy greater than 16 weeks.
6. Measurable disease by RECIST v1.1 including at least one not previously irradiated lesion that is ≥ 10 mm in the longest diameter (lymph nodes must have short axis ≥ 15 mm) as determined by CT.
7. Adequate haematological function: Hb ≥ 90.0 g/l with no requirement for blood transfusion in the last 28 days, neutrophils ≥ 1.5 x 10^9^/l, platelets ≥ 100 x 10^9^/l; coagulation: INR <1.4 (unless therapeutically anti-coagulated) and APPT ratio <1.4.
8. Adequate liver function: bilirubin ≤1.5 x ULN, transaminases ALT and AST ≤2.5x ULN. (AST or ALT <5x ULN allowed in the presence of parenchymal liver metastases.
9. Adequate renal function defined as calculated creatinine clearance using modified Wright or Cockcroft-Gault formula ≥ 51 ml/min or measured radioisotopic GFR ≥ 51ml/min.
10. Negative or trace proteinuria reading on urine dipstick. Patients with 1+ proteinuria on dipstick must have ≤1+ proteinuria on consecutive dipstick taken no less than 1 week later. Patients with ≥2+ proteinuria on dipstick must have 24 hour urinary protein excretion ≤1 g.
11. Adequately controlled thyroid function, with no symptoms of thyroid dysfunction.
12. Ability to swallow oral medication (tablets).
13. Willing to stop taking herbal supplements, and (if allocated to Arm 3) willing to not consume grapefruit or grapefruit juice, during the treatment period and for 30 days after end of trial treatment.

## Exclusion criteria

1. Prior treatment with dose-dense weekly paclitaxel.
2. Uncontrolled brain metastases or seizures. A scan to confirm the absence of brain metastases is not required.
3. Known positivity for hepatitis B, hepatitis C or HIV due to the risk of transmitting the infection through blood or other body fluids and potential for reactivation during treatment.
4. Resting ECG with QTc > 470 ms on 2 or more time points within a 24 hour period or family history of long QT syndrome.
5. Concomitant use of known strong CYP3A inhibitors (eg. itraconazole, telithromycin, clarithromycin, protease inhibitors boosted with ritonavir or cobicistat, indinavir, saquinavir, nelfinavir, boceprevir, telaprevir) or moderate CYP3A inhibitors (eg. ciprofloxacin, erythromycin, diltiazem, fluconazole, verapamil). The required washout period prior to starting olaparib is two weeks.
6. Concomitant use of known strong CYP3A inducers (e.g., phenobarbital, enzalutamide, phenytoin, rifampicin, rifabutin, rifapentine, carbamazepine, nevirapine and St John’s Wort) or moderate CYP3A inducers (e.g., bosentan, efavirenz, modafinil). The required washout period prior to starting olaparib is 5 weeks for enzalutamide or phenobarbital and 3 weeks for other agents.
7. Pregnant or lactating. Pregnancy status in women of childbearing potential will be confirmed via a serum or urine pregnancy test prior to randomisation, monthly during the treatment period, and at the end of treatment assessment.
8. Of childbearing potential AND not willing to ensure they use effective contraception throughout the treatment period and for six months following the end of treatment. Acceptable methods of contraception are:
   1. true sexual abstinence (when this is in line with the preferred and usual lifestyle of the participant)
   2. a combination of male condom **plus** one of:

- vasectomised sexual partner, with participant assurance that partner received post-vasectomy confirmation of azoospermia
- Tubal occlusion
- Intrauterine device provided coils are copper-banded
- Etonogestrel implants (e.g., Implanon®, Norplant®)
- Normal and low dose combined oral pills
- Hormonal shot or injection (e.g., Depo-Provera)
- Intrauterine system device (e.g., levonorgestrel-releasing intrauterine system -Mirena®)
- Norelgestromin/ethinyl estradiol transdermal system
- Intravaginal device (e.g., ethinyl estradiol and etonogestrel)
- Cerazette (desogestrel). Cerazette is currently the only highly efficacious progesterone based pill.

1. Side effects of previous treatments have not resolved to grade 1 or less, with the exception of alopecia that is considered related to cytotoxic chemotherapy.
2. Radiotherapy, chemotherapy, surgery or tumour embolisation within 28 days before the first dose of IMP.
3. Additional concurrent anti-cancer therapy.
4. Causes of malabsorption, e.g., uncontrolled diarrhoea or poorly controlled stoma.
5. Bowel obstruction, fistulae, impending fistulation seen on radiological imaging, or extensive rectosigmoid involvement by cancer.
6. Inadequately controlled hypertension, defined as ≥150/90 mmHg.
7. Prior or concurrent therapy with a PARP or VEGF inhibitor.
8. Known hypersensitivity to olaparib, cediranib or any of the excipients of the products.
9. Known hypersensitivity to paclitaxel that in the opinion of the investigator would prevent administration of a weekly paclitaxel regimen.
10. Exposure to an investigational agent within 30 days or 5 half-lives (whichever is the longer) prior to enrolment.
11. Considered a poor medical risk due to a serious, uncontrolled medical disorder, non-malignant systemic disease or active, uncontrolled infection. Examples include, but are not limited to, uncontrolled ventricular arrhythmia, recent (within 3 months) myocardial infarction, uncontrolled major seizure disorder, unstable spinal cord compression, superior vena cava syndrome, extensive interstitial bilateral lung disease on High Resolution Computed Tomography (HRCT) scan or any psychiatric disorder that prohibits obtaining informed consent.
12. Myelodysplastic syndrome (MDS), acute myeloid leukaemia (AML) or features suggestive of MDS/AML.
13. Other malignancy within the last 5 years except: adequately treated non-melanoma skin cancer, curatively treated in situ cancer of the cervix, ductal carcinoma in situ (DCIS), or other solid tumours including lymphomas (without bone marrow involvement) curatively treated with no evidence of disease for ≥5 years.
14. Prior allogeneic bone marrow transplant or double umbilical cord blood transplantation.

# Screening, Registration and Recruitment

## Participant identification

Potential participants will be under the care of a consultant who specialises in the treatment of endometrial cancer. Once a participant has been identified as potentially eligible to participate, the opportunity will be discussed with the patient, and she will be given a copy of the PIS and ICF. The patient will be given adequate time to consider the trial and given the opportunity to ask further questions. Investigators should keep a record of how many patients were considered for the trial by maintaining the participant screening log.

No sites will be opened as a Participant Identifying Centre. The trial will not be promoted through posters. Information about the trial may appear on websites, but the purpose of these sites is to provide information and not to promote the study to potential participants. If the CTR or Sponsor is contacted directly by a potential participant, then they will be asked to discuss the trial with their consultant.

## Screening logs

A screening log of all patients who were considered for potential participation in the trial will be kept by each site. This log will record whether the person was considered eligible based on their medical notes, whether they were approached, and whether they consented. A screening log enables any biases from differential recruitment to be detected. Copies of completed screening logs will be sent by site to the CTR periodically or when requested.

## Recruitment rates

A total of 129 participants will be recruited at an expected rate of 4 per month.

## Informed consent

Consent will be taken by a member of the trial team who is GCP trained, suitably qualified and experienced, and who has been delegated by the PI to undertake this activity (it is expected that, due to the poor prognosis patients are facing, consent will be taken by a medically qualified doctor). The participant’s written informed consent must be obtained using the trial ICF, which follows the PIS. The participant will be given sufficient time after the initial invitation to participate before commencing the consent process. The participant will also have the opportunity to question the PI, their GP or other independent parties to decide whether they will participate in the trial. The participant must personally sign and date the current approved version of the ICF before any trial specific procedures are performed.

Only when written informed consent has been obtained from the participant, and they have been randomised into the trial, will they be considered a trial participant.

One copy of the ICF will be given to the participant, the original copy will be kept in the ISF, and a further copy will be kept with participant’s hospital notes.

The consent process includes some optional consent in addition to the standard informed consent. The participant may choose to not consent to any, or all, of these optional parts without being excluded from the trial. These optional consents are for:

- Donation of blood samples.
- Donation of archived tissue samples collected before starting in the trial.

It will be clearly stated to the participant that they are free to withdraw from the trial at any time for any reason without prejudice to future care, and with no obligation to give the reason for withdrawal. After the participant has entered the trial, the clinician remains free to give alternative treatment to that specified in the protocol, at any stage, if they feel it to be in the best interest of the participant. However, the treatment given and reason for doing so will be recorded and the participant will remain within the trial for the purpose of follow-up and data analysis.

New safety information may necessitate changes to the PIS and ICF. In this event, it may be necessary to ask some, or all, participants to decide whether to re-consent or withdraw from the trial. Decisions on whether, and which, participants need to re-consent will be made by the TMG and documented in the TMF. The TMG will set timelines for the re-consent process to be completed. Some participants, for example those who are no longer receiving the IMP concerned, may not need to re-consent but should be informed of the new information as required by the TMG. The CTR will communicate local requirements to participating sites and initiate a process to track progress.

## Registration and Randomisation

### Registration

Participants will be registered to the trial after written informed consent has been obtained, and at least one day before randomisation. Sites will register a participant by emailing [COPELIA@cardiff.ac.uk](mailto:COPELIA@cardiff.ac.uk) . CTR staff will then register the participant on behalf of the site using a secure online service, with an offline system for registering available for occasions when the online service is not accessible. The registration process will issue each participant with a Screening ID which will be used in place of the Trial ID when required e.g., for plasma samples collected before randomisation.

### Randomisation

The participant’s research nurse and/or doctor will screen the participant to ensure that they meet all of the inclusion criteria and none of the exclusion criteria. Eligibility must be confirmed in the participant’s medical notes by the PI (or their medically qualified delegate as documented on the Site Delegation Log) prior to randomisation.

Similar to the registration process, sites will randomise a participant by emailing [COPELIA@cardiff.ac.uk](mailto:COPELIA@cardiff.ac.uk) CTR staff will then randomise the participant on behalf of the site. Participants will be randomised through a secure online service, using a computer-based minimisation algorithm with a random element. If the online service is not accessible at the time of randomisation, then a paper-based offline method for randomisation will be used. The randomisation procedures will be fully documented, reviewed, tested and approved prior to the start of the study.

Participants will be randomised into one of the three arms of the trial, with equal allocation between the control and the treatment groups (1:1:1). Forty-three participants will be recruited to each arm, making 129 participants recruited in total.

Randomisation will be balanced by cancer type and by the number of prior cytotoxic chemotherapy regimens for recurrent/ progressive disease (not counting adjuvant treatment).

Following randomisation, the participant will be assigned a Trial ID, which is the same as their Screening ID, and a confirmation email will be sent to the recruiting site and members of the trial team. From this point forward the Trial ID will be used in all correspondence and on all relevant trial documentation.

Upon randomisation, participants will be given a trial specific Participant Emergency Card which details the trial title and EudraCT code, the participant’s trial number and the contact details of the local PI and out of hours contact details in case of emergency.

# Withdrawal & lost to follow-up

## Withdrawal

Participants have the right to withdraw consent for participation in any aspect of the trial at any time. Participants’ care will not be affected by declining to participate or withdrawing from the trial.

If a participant initially consents but subsequently withdraws from the trial, clear distinction must be made as to which aspect(s) of the trial the participant is withdrawing from. In the COPELIA trial these aspects will be:

- Withdrawal of some or all trial treatment: e.g., if the participant is in a trial arm with two IMPs and one IMP is discontinued then this should be recorded here.
- Partial withdrawal from further data collection*: this is withdrawal from some, or all, further data collection that involves participant contact, but not withdrawal from remote follow-up (select from: sample collection, questionnaires, clinical assessments).
- Complete withdrawal from further data collection*: this is withdrawal from ALL further data collection including remote follow-up which does not require participant contact.
- Withdrawal of permission to use samples already collected*: if this is requested then no further sample collection will happen.

*For effective safety monitoring, participants cannot withdraw from further data-collection or follow-up without also withdrawing from all trial treatment.

Data collected before a participant withdraws consent may still be used. Samples collected before a participant withdraws consent may still be used unless the participant requests for the samples to be excluded from the trial.

If, during the trial, the participant loses the mental capacity to give continuing consent then the PI will withdraw the participant from trial treatment, and the person will be treated according to local standard NHS practice. Remote follow-up, which does not involve participant contact and which the participant had consented to, may continue and data and samples already collected may be used.

Participants may choose to withdraw consent verbally or in writing, and this will be documented in the participant’s medical notes. If a participant withdraws consent for participation, it is the responsibility of the PI to ensure the participant’s withdrawal is documented and implemented.

The PI may withdraw participants from the trial for the following reasons:

- Intolerance to treatment, usually CTCAE grade 3 or 4 AE or SAE
- Evidence of radiological disease progression based on RECIST v1.1
- Participant choice
- PI’s clinical judgement that withdrawal is in best interest of participant
- Symptomatic deterioration including participants who experience rapid deterioration before completion of 4 weeks of protocol treatment.
- Sponsor’s decision to terminate the trial
- Pregnancy in participant
- Incorrect enrolment
- Participant lost to follow-up
- Participant is persistently non-compliant with the trial protocol
- Participant loses mental capacity to give continuing consent

All the results of the evaluations and observations, together with the reason for withdrawal from the trial (if known), must be recorded in the participant’s medical notes and in the CRF.

Participants who are withdrawn from some, but not all trial treatment (i.e. in Arm 2 or 3 and one IMP is discontinued) will continue with trial visits as per the schedule. Participants who are withdrawn from all trial treatment, but who have not withdrawn their consent for follow-up, will continue with follow-up assessments (and reporting of SAEs) as per other participants who have ended treatment (see section 13).

If a withdrawn participant is due to start a new therapeutic intervention, then, whenever possible, the end-of-treatment assessment should be completed before starting the new therapy. This means that the end-of-treatment assessment, which is usually done 30 days after the end of treatment, may be done sooner. If it is not possible to complete the assessment before starting the new therapy, then it should be done as soon as possible after. At their discretion, the PI may also schedule the end-of-treatment assessment (or the collection of research samples) sooner than 30 days after the end of treatment, for example if the participant is going on holiday.

Participants will only be withdrawn from further follow-up if the participant explicitly states that she does not wish to be followed-up further in the trial; if the participant dies or the end of the trial has been reached.

For monitoring of safety, all participants will be encouraged to attend the end-of-treatment assessment. If the participant does not attend their end-of-treatment assessment, or if the end-of-treatment assessment has been done earlier than 30 days after the end of treatment (as discussed above), then the participant’s notes will be reviewed after 30 days to record whether an SAE has occurred.

## Lost to follow up

Participants who cease to attend trial visits prior to the end of the follow-up period, or for whom remote follow-up is unsuccessful, will be classed as lost to follow up if we do not have confirmation of disease progression or death.

Every effort will be made to obtain follow-up information on these participants, unless they have completely withdrawn from the trial. Participants who are not present for a scheduled visit will be contacted by their local research team by telephone or letter. If they are contactable, the local research team will ask them to make an appointment to be seen at the next available clinic. If the participant declines or cannot be contacted, the local research team will inform their GP and will aim to complete the remote follow-up as per the study schedule. The minimum information we will aim to collect is date of death and SAE data.

If the participant is alive but not compliant (as agreed with the CI, see section 11.12) with trial medication or the approved visit schedule, they will be withdrawn from trial medication and all further data collection that involves participant contact, and their data collected up until the point of withdrawal will be used for analysis. Participants lost to follow up will not be replaced.

# Trial Intervention

## Treatment(s)

### Paclitaxel

Paclitaxel is an anti-microtubule cytotoxic drug that promotes the assembly of microtubules from tubulin dimers and stabilises microtubules by preventing depolymerisation. It is licensed for the treatment of ovarian, breast, advanced non-small cell lung cancers and AIDS-related Kaposi’s sarcoma. The use of paclitaxel in endometrial cancer is outside the drug license, but its use in combination with carboplatin is the worldwide standard-of-care for the first-line treatment of advanced or recurrent disease (Miller et al. 2012).

The administration of paclitaxel on a weekly schedule is a commonly used treatment strategy in standard clinical practice for the treatment of recurrent ovarian, breast and endometrial cancers.

Sites should use the current SmPC for the brand of paclitaxel they use to guide the clinical management of participants receiving paclitaxel.

### Cediranib

Cediranib is an orally bioavailable VEGF-R1, -R2 and R3 tyrosine kinase inhibitor. Recent data from ICON6 have shown that the drug improves PFS with a trend towards improved OS in recurrent platinum-sensitive ovarian cancer (Ledermann et al 2014). At this time the drug is not licensed.

Cediranib (AZD2171) is a potent small molecule vascular endothelial growth factor (VEGF) receptor tyrosine kinase inhibitor of all three VEGF receptors (VEGFR-1, -2 and -3) at nanomolar concentrations. Inhibition of VEGF signalling leads to the inhibition of angiogenesis, lymphangiogenesis, neovascular survival and vascular permeability. Cediranib has additional activity against stem cell factor receptor (c-kit) tyrosine kinase, inhibiting this kinase with a similar potency to that at which it inhibits VEGFRs. Cediranib is less active versus platelet-derived growth factor receptor (PDGFR) tyrosine kinases, and inactive against other kinases tested (Matulonis et al 2009).

Cediranib inhibited the growth of tumours in preclinical models in a dose-dependent manner. At doses that reduce tumour growth, VEGFR-2 and c-kit were inhibited, but only partial inhibition of PDGFR was observed. Anti-tumour activity was associated with a reduction in micro-vessel density and changes in vascular permeability. Cediranib reduced ascites accumulation in pre-clinical models, and in several models also inhibited metastatic dissemination, also blocking VEGFR-3 inhibited lymphangiogenesis. Collectively, these changes indicate that cediranib limits tumour growth, metastases and microvascular permeability. Following once daily dosing with 20 mg cediranib, the unbound minimum steady-state plasma concentration (Css, min) was approximately 5-fold greater than the human umbilical vein endothelial cell (HUVEC) proliferation inhibitory concentration 50 % (IC50) reported in non-clinical studies (Wedge et al 2005).

At a clinical dose of 20 mg in patients, there was a small increase in diastolic blood pressure (DBP) and systolic blood pressure (SBP); a significant reduction in serum soluble VEGFR2 was observed; and a decrease in tumour vessel permeability and vascularity in liver lesions, as measured by dynamic contrast enhanced magnetic resonance imaging, was detected.

### Olaparib

Olaparib (Lynparza^TM^) is licensed for the maintenance treatment of epithelial ovarian cancer after response to platinum-based chemotherapy in patients with germline BRCA mutations. In COPELIA, the use of olaparib is outside of the license.

Olaparib is a potent inhibitor of human poly-ADP ribose polymerase enzymes (PARP1, PARP2 and PARP3). PARP is required for the efficient repair of DNA single strand breaks, and an important aspect of PARP-induced repair requires that after chromatin modification, PARP auto-modifies itself and dissociates from the DNA to facilitate access for base excision repair (BER) enzymes. When olaparib is bound to the active site of DNA-associated PARP it prevents the dissociation of PARP and traps it on the DNA, thus blocking repair. In replicating cells this leads to DNA double strand breaks (DSBs) when replication forks meet the PARP DNA adduct. In normal cells, homologous recombination repair (HRR), which requires functional BRCA1 and 2 genes, is effective at repairing these DNA double strand breaks. In the absence of functional BRCA1 or 2, or when other defects in the HRR pathway exist, DNA DSBs cannot be repaired via HRR. Instead, alternative and error prone pathways are activated, such as the nonhomologous end-joining pathway, leading to increased genomic instability. After a number of rounds of replication, genomic instability can reach intolerable levels resulting in cancer cell death, as cancer cells have a high DNA damage load relative to normal cells. In BRCA-deficient *in vivo* models (used as an exemplar of HRR deficiency), olaparib given after platinum treatment resulted in a delay in tumour progression and an increase in OS compared to platinum treatment alone; an effect that was corroborated in ovarian cancer patients with confirmed deleterious or suspected deleterious BRCA mutation (i.e., a mutation that disrupts normal gene function) in either the germline or the tumour (detected using an appropriately validated test).

Following oral administration of olaparib via the tablet formulation, absorption is rapid. Co-administration of food slows the rate of absorption (tmax delayed by 2.5 hours and Cmax reduced by 20 %). However, food did not significantly affect the AUC. Therefore, olaparib can be taken without regard to food (exceptions may be required when taken with cediranib, see section 11.4.3). Note that this advice differs from the advice with the marketed capsule formulation which should not be taken with food.

Olaparib can be administered in patients with mild renal impairment (creatinine clearance ≥ 51 ml/min). There are limited data in patients with moderate impairment (creatinine clearance ≤ 50 ml/min) or severe impairment (creatinine clearance ≤ 30 ml/min) and hence these patients are excluded from entry into the COPELIA trial (participants who develop moderate impairment during the trial may have a dose reduction of olaparib, see section 11.5.3.1.4). Olaparib is not recommended for use in patients with hepatic impairment (serum bilirubin > 1.5 time ULN) and so these patients are also excluded from the trial.

## Treatment supply and storage

### Supply

Paclitaxel 6 mg/ml concentrate for solution for infusion, will be sourced from standard hospital stock.

Cediranib and olaparib (Table 1) will be supplied by AstraZeneca Pharmaceuticals Ltd. Descriptive information for cediranib and olaparib can be found in their respective Investigator’s Brochures (IB).

Cediranib will be supplied for oral administration as 15 mg and 20 mg plain tablets, equivalent to 18.9 or 25.2 mg of cediranib maleate respectively. The tablets are round bi-convex beige film coated tablets, 7 mm (15 mg) and 8 mm (20 mg). Tablets will be provided to participants in high-density polyethylene bottles (each bottle contains one strength of tablet) with tamper evident seals.

Olaparib is presented for oral administration as a green film-coated tablet containing 100 mg or 150 mg of drug substance. Tablets will be provided to participants in high-density polyethylene bottles (each bottle contains one strength of tablet) containing desiccant and with tamper evident seals.

| **Investigational product** | **Dosage form and strength** | **Manufacturer** |
| --- | --- | --- |
| Cediranib (AZD2171) | 15 and 20 mg tablets | AstraZeneca |
| Olaparib (AZD2281) | 100 and 150 mg tablets | AbbVie Deutschland GmbH & Co |

Table 1: Cediranib and olaparib formulation.

Guy’s and St Thomas’ NHS Foundation Trust will over label the cediranib and olaparib, provide Qualified Person (QP) release certification, and distribute the cediranib and olaparib on behalf of the Sponsor. Prior to distribution, cediranib and olaparib will be stored at Guy’s and St Thomas’ NHS Foundation Trust in temperature monitored facilities. Labels will be prepared in accordance with Good Manufacturing Practice Annex 13 requirements for labelling. All cediranib and olaparib is only to be used by the named investigators, for the participants recruited to this trial.

Prior to opening to recruitment, all participating site pharmacies will receive a shipment of cediranib and olaparib which is appropriate for anticipated levels of participant recruitment. Throughout the trial, the site Pharmacy team will liaise with the TM to ensure adequate cediranib and olaparib levels are kept on site for the number of participants enrolled. Further shipments of cediranib and olaparib may be delivered to site pharmacies throughout the trial if required (procedures for ordering cediranib and olaparib are detailed in the Pharmacy Manual).

Cediranib and olaparib will be temperature monitored during transit to sites. Receipt of cediranib and olaparib at participating site pharmacies will be fully documented using logs and forms provided by the CTR. This documentation includes information on when the IMP was received, as well as the amount, condition and integrity (including confirmation that the IMP remained within the acceptable temperature range) of the cediranib and olaparib received. Originals of receipt documentation will be filed in the Pharmacy Site File. Copies of receipt documentation will be sent promptly by Pharmacy to the CTR who will acknowledge receipt of the documentation back to Pharmacy. Any concerns over the condition of the cediranib and olaparib received, or discrepancies in the amount received, will be investigated by the CTR immediately. The Sponsor, AstraZeneca representative, or the distributor, may need to be consulted to determine the appropriate action to be taken. Decisions will be communicated to the Pharmacy and documented in the TMF.

### Storage

#### Paclitaxel

Paclitaxel will be dispensed directly from standard hospital stock and will therefore be stored according to the standard requirements for paclitaxel and with the pharmacy’s local procedures for temperature monitoring.

#### Cediranib and olaparib

The participating site pharmacy will store cediranib and olaparib in their original containers in a secured area in accordance with applicable regulatory requirements.

A calibrated temperature monitoring device will be used to record the temperature conditions in the drug storage facility. The recommended storage temperatures, and actions to take in the event of a temperature excursion, are shown in Table 2.

|  | **Cediranib** | **Olaparib** |
| --- | --- | --- |
| Acceptable storage temperature | 2-30°C | 0-30°C |
| Action to take if storage temperature outside the acceptable ranges above: | Pharmacy:   - immediately return drug to recommended storage temperature - quarantine drug until instructions received from CTR - immediately report details of temperature excursion to CTR - implement actions from CTR   CTR:   - if required, liaise with Sponsor and AstraZeneca representative for decision on action - inform Pharmacy of action to be taken - ensure reported temperature excursions and communications/decisions on actions are documented in the TMF | |

Table 2: Cediranib and olaparib: acceptable storage temperatures and actions to be taken in event of temperature excursion.

In the event of a temperature excursion, CTR will use the current advice issued by AstraZeneca to guide the actions required by pharmacy at site. Sites must quarantine the drug until suitability has been confirmed by CTR—they must not make their own judgements as it is possible the advice from AstraZeneca may change during the study period. For all excursions, CTR will document the packs affected, the temperatures reached and the duration at outlier temperatures.

CTR may obtain guidance from the Sponsor and AstraZeneca on a case-by-case basis if there are concerns over the extent or frequency of temperature excursions.

### Destruction

Paclitaxel used in this study is from hospital stock and therefore permission to destroy is not required from the Sponsor.

Approval of the Sponsor (obtained via the CTR) must be obtained before any trial stock of cediranib or olaparib is destroyed. A detailed description of the IMP destruction procedure will be provided in the Pharmacy Manual which will be issued to participating sites by the CTR. On receipt of permission to destroy IMP, IMP can be destroyed as per local procedures and with the completion of the required trial paperwork. This paperwork includes recording the destruction in the site accountability log and site destruction log and creating a destruction certificate. Copies of the destruction paperwork will be sent to the CTR and the originals stored in the PSF.

## Treatment prescribing and dispensing

Upon randomisation of the participants, the CTR will inform the participating centre pharmacist and research team of the participant allocation via email. The email does not allocate particular pack numbers.

Pharmacy will receive trial specific prescriptions, signed by a delegated member of the research team, for each participant per cycle, with additional prescriptions if dose-reductions are required mid-cycle. Pharmacy will select the appropriate IMP(s) which will then be dispensed in accordance with the prescription, and Pharmacy will ensure the required accountability forms in the PSF are completed.

A maximum of 2 months’ worth of oral IMP can be dispensed at a visit if the participant is unable to attend site as required from cycle 3 for participants on Arms 2 & 3 only. Cycle 1 and 2 of IMP for participants on Arms 2 & 3 must continue monthly dispensing to allow Investigators to assess whether the patient is tolerating treatment well and gaining clinical benefit. Confirmation that a patient is fit for treatment must be assessed by the clinician using the assessments at the protocol defined timepoints before patients are given the go-ahead to continue with the next cycle.

Postal or delivery via next day service such as courier or Royal Mail Special Delivery is permitted at PI discretion if patient cannot attend a trial site and is being monitored by telephone assessment. The participant must consent verbally (and this should be documented in their notes) to providing contact details for shipping purposes. A follow up phone call could be used to confirm they have received the oral IMP.

## Dosing schedule

### Dosing schedule: Paclitaxel

Paclitaxel will be administered at a dose of 80 mg/m^2^ on days 1, 8 and 15 of a 28 day cycle for a maximum of 6 cycles (see section 11.5.1 for dose modifications).

Before each paclitaxel infusion, the results from the blood tests must be checked to ensure treatment can be given. The infusion should only be given if all the required parameters are met. Table 3 shows the required blood values and the action to be taken if any parameter is not met. Treatment decisions should be based on bloods taken as recently as possible—it is expected that in most cases this will be up to 2 days prior to the planned treatment administration, though up to 4 days is permitted where this is essential (for example where the infusion is immediately after a bank holiday weekend) and if allowed by local procedures.

Note that for participants in Arm 2, cediranib treatment can continue (it should not be started for participants on C1D1) whilst paclitaxel is paused or stopped provided the advice in the cediranib toxicity section is adhered to.

If the participant is unable or unsuitable to receive paclitaxel on the planned day, for example due to a missed visit or illness, then if the planned treatment was for:

- D1, then defer D1 until the next week
- D8 or D15, then, if possible, give treatment within 3 days of scheduled date. If this is not possible, then omit treatment and the next treatment should be given in accordance with original timeline.

| **Day** | **Required blood parameters** | **Action if any parameter is not met** |
| --- | --- | --- |
| 1 | Neutrophil count (ANC) ≥ 1.5 x 10^9^/l  Platelet count ≥ 100 x 10^9^/l  Bilirubin < 1.5 x ULN  AST/ALT <2.5 x ULN (or <5 x ULN in the presence of liver metastases) | Interrupt treatment and repeat bloods at least weekly until recovery. Guidance on recommencing treatment and appropriate dose reductions are given in Table 4. |
| 8, 15 | Neutrophil count (ANC) ≥ 1.0 x 10^9^/l  Platelet count ≥ 75 x 10^9^/l | Omit treatment. Repeat bloods at least weekly until recovery. Guidance on recommencing treatment and appropriate dose reductions are given in Table 4. |

Table 3: Required blood parameters for paclitaxel infusion.

Dose banding using the NHS England dose-banding tables for paclitaxel (https://www.england.nhs.uk/wp-content/uploads/2016/03/pss-cquin-schemes.pdf) is permitted where this is standard local practice. The use of other banding protocols must be notified to and approved by the Sponsor prior to trial initiation.

Dose capping is not recommended. However, sites may use dose-capping if this is their standard local practice and if this is notified to and approved by the Sponsor prior to trial initiation.

Prior to administration of paclitaxel, hypersensitivity prophylaxis, including H1/H2 antagonists and corticosteroids, should be given as per local procedures, for example, 30 minutes prior to paclitaxel one of the following may be administered:

- - - Dexamethasone 7.6-8.0 mg IV. If participants are unable to tolerate weekly dexamethasone at this dose and have not experienced paclitaxel hypersensitivity, the dose can be gradually reduced at the investigator’s discretion. If a hypersensitivity reaction, then develops, dexamethasone should be reintroduced at least 7.6-8.0 mg IV prior to ALL subsequent paclitaxel infusions.
    - Chlorphenamine 10 mg IV, given as per local practice.
    - Ranitidine 50 mg IV (in 20 ml sodium chloride 0.9 % over 2 minutes).

Immediately pre-chemotherapy, administer anti-emetics as per local procedures. Ondansetron and related drugs which impact the QTc interval should be used with caution.

Anaphylaxis precautions should be available during infusion for the emergency treatment of hypersensitivity reactions.

Reconstitute and administer paclitaxel via a non-PVC giving set and connectors incorporating a filter ≤ 0.22μm.

Reconstitute paclitaxel 80 mg/m^2^ to a target concentration between 0.3 mg/ml and 1.2 mg/ml in sodium chloride 0.9 %, or glucose 5 %, according to local standard practice.

Give paclitaxel IV over one hour via a rate-controlling device.

Monitor closely for allergic reactions and cardiac arrhythmias as per local procedures.

Use of a cold cap is permitted.

N.B. on extravasation, paclitaxel is a vesicant. Local procedures for the management of extravasation should be followed.

### Dosing schedule: Cediranib

All participants receiving cediranib will start the trial at a dose of 20 mg once daily. On the first day of each 28-day cycle, participants will be given one bottle containing 35 tablets. Further cediranib may be dispensed mid-cycle if necessary to manage dose reductions. Within each bottle all tablets will be the same strength (either 20 mg or 15 mg).

Treatment decisions should be based on bloods taken as recently as possible—it is expected that in most cases this will be up to 2 days prior to the dispensing day, though up to 4 days is permitted where this is essential (for example over a bank holiday weekend).The 20 mg dose of cediranib can be taken with or without a light meal or snack (e.g., two pieces of toast or a couple of biscuits), in a similar way each morning. If cediranib is dose reduced to 15 mg per day then it has to be taken on an empty stomach to preserve its activity—i.e. it should be taken at least 1 hour before, and at least 2 hours after, food ingestion.

Cediranib should be swallowed whole with approximately 240 ml of water and not chewed, crushed, dissolved or divided. If vomiting occurs shortly after a cediranib tablet is swallowed, the dose should only be replaced if the tablet can be seen to be intact. If a scheduled dose is missed, the participant will be allowed to take the scheduled dose up to a maximum of 2 hours after the scheduled dose time. If greater than 2 hours after the scheduled dose time, the missed dose should not be taken, and the participant should take a single dose at the next scheduled time. The number of omitted doses will be recorded in the participant’s diary and transferred to the participant’s notes and the CRF. If a participant’s toxicity has been managed through a dose reduction or a 5 days on/ 2 days off approach, this will also be documented in the participant’s notes and CRF.

### Dosing schedule: Olaparib

All participants receiving olaparib will start the trial at a dose of 300 mg twice daily, equivalent to a total daily dose of 600 mg. On the first day of each 28-day cycle, participants will be given four bottles containing 32 tablets each. Further olaparib may be dispensed mid-cycle if necessary to manage dose reductions. Within each bottle all tablets will be the same strength (either 100 mg or 150 mg). Once a bottle has been opened the tablets cannot be taken after 3 months.

Treatment decisions should be based on bloods taken as recently as possible—it is expected that in most cases this will be up to 2 days prior to the dispensing day, though up to 4 days is permitted where this is essential (for example over a bank holiday weekend).

In this trial, olaparib is given in combination with cediranib, and this influences the way olaparib should be taken. The daily dose of cediranib and the morning dose of olaparib can be taken together and should be taken at a similar time each day; the evening dose of olaparib should be taken 12 hours after the morning olaparib dose.

- If cediranib is being taken at a dose of 20 mg per day (or if cediranib is being withheld) then the morning doses of olaparib and cediranib can be taken with or without a light meal or snack (e.g., two pieces of toast or a couple of biscuits).
- If cediranib has been dose reduced to 15 mg per day then the morning doses of olaparib and cediranib must be taken on an empty stomach—i.e. at least 1 hour before, and at least 2 hours after, food ingestion.
- The evening dose of olaparib can be taken without food, or with a light meal or snack (e.g., two pieces of toast or a couple of biscuits), irrespective of the cediranib dose.

Olaparib should be taken with approximately 240 ml of water, and should be swallowed whole and not chewed, crushed, dissolved or divided. If vomiting occurs shortly after olaparib tablets are swallowed, the dose should only be replaced if all of the intact tablets can be seen and counted. If a scheduled dose is missed, the participant will be allowed to take the scheduled dose up to a maximum of 2 hours after the scheduled dose time. If greater than 2 hours after the scheduled dose time, the missed dose is not to be taken, and the participant should take a single dose at the next scheduled time. The number of omitted doses will be recorded in the participant’s diary and transferred to the participant’s notes and the CRF. If a participant’s toxicity has been managed through a dose reduction this will also be documented in the participant’s notes and CRF.

## Dose modifications for toxicity

For many toxicities, interruptions in treatment will be used to manage the condition and allow recovery.

If the administration of any IMP is interrupted due to toxicities for a continuous period of 4 weeks or more then administration of that IMP to the participant will be discontinued permanently. If cediranib has been withheld for 2 weeks due to toxicity, then advice from the CI (via the CTR) should be obtained before continuing dosing.

If an IMP is discontinued, then a withdrawal form should be completed (see section 10.1) and the following will be considered:

- **Arm 1:** If paclitaxel is discontinued for a participant in Arm 1 then the participant must be withdrawn from trial treatment (follow-up continues).
- **Arms 2 and 3:** If a participant has developed intolerable toxicities related to one of the IMPs, but (in the opinion of the investigator) the participant is experiencing ongoing clinical benefit and therefore the risk-benefit remains favourable, then it may be possible (see below) for the participant to continue only on the unrelated drug and remain in the trial (a withdrawal form will be completed for the discontinued IMP). If it is not considered appropriate for the participant to remain on the unrelated drug, then this will also be discontinued, and the participant will be withdrawn from all trial treatment (follow-up continues).
  - Arm 2: it is not necessary for the investigator to seek CI approval to continue a participant only on the unrelated drug and remain in the trial.
  - Arm 3: the investigator should contact the CI (via the CTR), on a case-by-case basis, to gain written approval to continue a participant only on the unrelated drug.

In this study, both experimental arms (Arms 2 and 3) involve concurrent administration of two drugs. The drugs have a partially overlapping toxicity spectrum and investigators should evaluate carefully which drug is the likely cause of adverse events on treatment. Investigators should refer to the sections for both IMPs being taken when deciding the toxicity management strategy.

### Paclitaxel dose modifications

The dose levels for Paclitaxel are as follows:

- Starting dose: 80 mg/m^2^
- Dose Level 1: 65 mg/m^2^
- Dose Level 2: 50 mg/m^2^
- Dose Level 3: STOP

#### Management of paclitaxel-related toxicities

There are no paclitaxel dose reductions planned for nausea, vomiting, diarrhoea, constipation or venous thrombo-embolism. These should be managed with standard appropriate supportive measures according to local guidelines.

Recommendations for the management of key paclitaxel-related toxicities are listed in Table 4. For any other adverse event of CTCAE Grade 4 considered at least possibly related to weekly paclitaxel, treatment should be discontinued until the PI and CI have agreed on the most appropriate course of action.

| **Event** | **Toxicity** | **Recommended action** |
| --- | --- | --- |
| Neutropenia | Grade 1 or worse (ANC <1.5 x10^9^/l) on D1 | Interrupt treatment until recovered to ≥ 1.5 x 10^9^/l. If recovery occurs in ≤ 1 week then continue at current dose. If recovery takes > 1 week then either use prophylactic G-CSF or reduce to next available dose level. |
|  | Grade 3 or worse (ANC <1.0 x10^9^/l) on D8 or D15 | Omit current dose. At next scheduled dose either use prophylactic G-CSF or reduce to next available dose level. |
| Febrile neutropenia | Grade 3 or worse  (ANC <1.0 x10^9^/l and temperature ≥38^o^C) | Interrupt treatment until recovery and then either use prophylactic G-CSF or reduce to next available dose level. |
| Thrombocytopenia | Platelets <100x10^9^/l on D1 | Interrupt treatment until recovered to ≥ 100x 10^9^/l. If recovery occurs in ≤ 1 week then continue at current dose. If recovery takes > 1 week then reduce to next available dose level. |
|  | Grade 1 or worse (platelets <75 x 10^9^/l on D8 or D15) | Omit current dose. At next scheduled dose reduce to next available dose level. |
|  | Grade 4 or worse (platelets <25 x 10^9^/l), or bleeding associated with Grade 3 or worse (<50 x 10^9^/l) | Interrupt treatment until platelet recovery (must be ≥ 100x 10^9^/l for D1 and ≥ 75x10^9^/l for D8 and D15) and reduce to next available dose level. |
| Liver function tests | Bilirubin >1.5 x ULN  AST/ALT >2.5 x ULN (or >5 x ULN in the presence of liver metastases) on D1 | Interrupt treatment until recovery and evaluate for alternative cause. If recovery occurs in ≤ 1 week or an alternative cause is demonstrated continue at current dose. If recovery takes >1 week then reduce to next available dose level. |
| Neuropathy (peripheral sensory or peripheral motor) | Grade 2  ≥ Grade 3 | Interrupt paclitaxel until recovery to ≤ grade 1 and then reduce to next available dose level. If recovery takes >4 weeks stop paclitaxel.  Discontinue paclitaxel. |
| Mucositis (any type) | ≥ Grade 3 | Interrupt paclitaxel until recovery to ≤ grade 1 and then reduce to next available dose level. |
| Rash (commonly affecting dorsal surface of hands/ forearms) | ≥ Grade 2 | Use supportive management such as emollients, analgesia and antihistamines at investigators discretion.  Interrupt paclitaxel until recovery to ≤ grade 1 and then reduce to next available dose level. |
| Pneumonitis or Pneumocystis pneumonia (PCP) | Any grade | Discontinue paclitaxel. |

Table 4: Paclitaxel dose modifications.

#### Management of paclitaxel-related hypersensitivity reactions

Acute management of any hypersensitivity reactions will follow local standard of care. Re-challenge with paclitaxel at full dose following a grade 1-2 hypersensitivity reaction is permitted if the PI feels that this is in the participant’s best interests. Local standard of care should be followed, and consideration should be given to increased prophylactic medications and slowing of initial infusion rates.

Re-challenge following a grade 3-4 hypersensitivity reaction is not permitted.

### Cediranib dose modifications

A single dose reduction to 15 mg once daily and the use of a 5 days on/ 2 days off dosing schedule are allowed to manage treatment-related toxicity. The 5 days on/ 2 days off dosing schedule allows the investigator flexibility in managing cediranib related toxicities but is not considered a dose reduction. The 5 days on/ 2 days off schedule can be implemented with any cediranib dose. The 7 days on schedule can be reinstated if the investigator believes this to be in the best interest of the participant and repeated 5 days on/ 2 days off schedules are allowed.

Note that if cediranib is dose reduced to 15 mg per day then it has to be taken on empty stomach to preserve its activity (see section 11.4.2).

The dosing levels for cediranib are as follows:

- Starting dose: 20 mg once daily
- Dose Level 1: 15 mg once daily
- Dose Level 2: STOP

Cediranib will be administered with paclitaxel (Arm 2) or olaparib (Arm 3).

#### Management of cediranib-related toxicities

The toxicities associated with VEGF pathway inhibitors such as cediranib are well known and there are established management protocols to address these toxicities. The most common side effects observed in patients treated with cediranib include hypertension, diarrhoea, nausea, fatigue and proteinuria. Thrombo-embolism, fistula formation, GI perforation and posterior reversible encephalopathy syndrome (PRES) have also been associated with cediranib use.

N.B. The IB for cediranib refers to PRES, but as this is not listed in the CTCAE version 4.03, the condition will be reported on the CRF as reversible posterior leukoencephalopathy syndrome (RPLE).

Any participant who experiences one of the complications listed below should permanently discontinue cediranib:

1. Any grade of fistula.
2. GI perforation.
3. Grade 4 diarrhoea.
4. Grade 4 hypertension despite maximal anti-hypertensive treatment.
5. PRES/RPLE
6. Arterial thrombo-embolism (e.g., myocardial infarction or cerebrovascular accident).
7. Grade 4 venous thrombo-embolism.
8. Severe (Grade 3 or 4) haemorrhage.

The recommended management of specific toxicities is as follows:

##### Hypertension

The goal of managing cediranib-associated hypertension is to maintain blood pressure below 150/90. Because of hospital-related exacerbations of hypertension (white-coat phenomenon), participants should be encouraged to acquire a portable blood pressure monitor and take regular home readings. The participants blood pressure machine should be calibrated against a hospital machine to ensure the correct readings are obtained. Home readings of blood pressure will be taken into consideration when making decisions on initiating anti-hypertensive therapy. All readings including repeat readings, home readings or readings taken GP must be recorded in the participants notes as evidence that the participant does not have inadequately controlled hypertension, defined as ≥150/90 mmHg.

For participants taking cediranib, if serial measurements identify blood pressure readings that exceed the threshold of 150/90 mmHg, participants will be commenced on anti-hypertensive therapy with one or more of a calcium channel antagonist (e.g., amlodipine), an ACE inhibitor (e.g., enalapril) or another anti-hypertensive drug. If a participant is already taking anti-hypertensives, the dose of these should be increased or an additional drug added. Management of hypertension will be in keeping with local practice and the exact choice of anti-hypertensive agents will be influenced by co-morbid conditions and the toxicities of anti-hypertensive agents.

Participants who develop CTCAE grade 4 hypertension (life-threatening consequences e.g., hypertensive crisis, transient or permanent neurologic deficit) or PRES/RPLE should permanently discontinue cediranib.

##### Proteinuria

Proteinuria is a common but usually clinically non-significant toxicity. Proteinuria will be monitored by dipstick examination of urine every 4 weeks in clinic. If the urine contains at least 3+ protein, a sample will be sent to test for infection, and a 24 hour collection will be made to quantify the protein output. If urinary protein excretion is more than 2 g/24 hours, cediranib will be held until the toxicity has resolved to grade 1 or less (<1 g/ 24 hours) in the absence of any urinary tract infection and then recommenced at the next available dose level. It is recommended that 24-hour urinary protein excretion is repeated weekly until resolution.

##### Thrombo-embolic events

The prevalence of venous thrombo-embolism is slightly increased in patients receiving VEGF inhibitors.

For participants who develop thrombosis/embolism during cediranib treatment, the following actions are recommended:

- Cediranib should be permanently discontinued in participants who develop any grade of arterial thromboembolic event e.g., myocardial infarction or ischaemic cerebrovascular event.
- Grade 2 Venous thromboembolic event: commence therapeutic dose anti-coagulant therapy with low molecular weight heparin. Cediranib can be continued or held for up to 2 weeks at clinician’s discretion.
- Grade 3 or incidentally discovered pulmonary embolus: hold cediranib for up to 2 weeks. Commence therapeutic-dose low molecular weight heparin. If stable within 2 weeks recommence cediranib; if not stable within 2 weeks, then the PI and CI will agree on the most appropriate course of action.
- Symptomatic Grade 4 venous thromboembolic event: permanently discontinue cediranib.

##### Diarrhoea

Diarrhoea has been commonly reported in patients receiving cediranib. 11 % of patients receiving 20 mg once daily in the maintenance phase of ICON6 trial suffered grade 3 diarrhoea. It is important to adopt a clear management strategy to rapidly control symptoms associated with diarrhoea.

All participants randomised to receive cediranib in COPELIA should be counselled that they are likely to experience diarrhoea during treatment and prescribed loperamide to use in case diarrhoea occurs. If diarrhoea develops, they should immediately commence loperamide and inform the local PI or research nurse. Guidance on management, dependent on severity of diarrhoea is given in Table 5. The possibility of neutropenic colitis should be considered in participants with grade 3 or 4 diarrhoea.

| **Severity** | **Recommended action** |
| --- | --- |
| **Grade 1** ( Increase of <4 stools per day from baseline) | Commence loperamide as recommended. Follow dietary advice and ensure adequate fluid intake. Continue cediranib dosing. |
| **Grade 2** (increase 4-6 stools per day or nocturnal diarrhoea) | Commence loperamide and consider addition of codeine phosphate if already taking loperamide. Follow dietary advice and ensure adequate fluid intake. If not reduced to ≤ grade 1 within 24 hours, hold cediranib for at least 48 hours. Resume dosing when ≤ grade 1 at same dose. If grade 2 diarrhoea recurs, reduce cediranib dose to next dose level at recovery, or consider 5 days on, 2 days off dosing at current dose. |
| **Grade 3** (increase of ≥ 7 stools per day or requiring intravenous rehydration or hospitalisation) | Commence loperamide and codeine phosphate, intravenous hydration as required. Hold cediranib for at least 48 hours and until recovery to ≤ grade 1. On recovery reduce cediranib to next dose level or consider 5 days on, 2 days off dosing at current dose. Consider dose reduction of paclitaxel by one dose level. |
| **Grade 4** (life-threatening consequences) | Commence loperamide and codeine phosphate. Resuscitate. Permanently discontinue cediranib. |

Table 5: Management of cediranib-associated diarrhoea.

If cediranib dosing is held due to diarrhoea for longer than 2 weeks, advice should be sought from the CI (via contact with the CTR) before restarting cediranib.

Weekly paclitaxel may also cause low grade diarrhoea. If Grade 2 or Grade 3 diarrhoea recurs despite a cediranib dose reduction during concomitant cediranib-paclitaxel treatment, reduce paclitaxel by one dose level.

The recommended loperamide regimen is 4 mg at first episode of loose stool and then 2 mg every 2-4 hours until participant is free from diarrhoea for at least 12 hours. Alternative regimens consistent with established local practice may be used. Codeine phosphate should be administered according to local guidelines.

Participants should be advised to modify their diet during episodes of diarrhoea as an adjunct to loperamide treatment. Current best recommendations are to; eat low-fat, high protein food and stay away from fatty, high-fibre or spicy foods. Eat cooked vegetables instead of raw vegetables. Take off the skin of fruits before eating them. Stay away from milk, milk products and herbal supplements. Eat two grated apples a day that have been left after grating for at least one hour to oxidise (skin peeled off).

##### Fatigue

Mild to moderate fatigue is commonly seen with cediranib therapy and can be rapid in onset. Grade 3 fatigue was seen in 16 % patients when cediranib was given in combination with chemotherapy and 6 % patients during cediranib maintenance therapy in the ICON6 trial. Fatigue can often be managed successfully with short treatment breaks without reducing cediranib dose and participants should be advised to seek advice from the research team early in the event of ≥ Grade 2 fatigue (not relieved by rest and interfering with normal levels of activity).

The research team should evaluate for other causes of fatigue, in particular other cediranib-related toxicities such as diarrhoea and hypothyroidism. Advice on cediranib dosing in the event of fatigue is given in Table 6. If fatigue recurs during concurrent cediranib-paclitaxel treatment despite following the advice in Table 6 consider reducing paclitaxel dose by one dose level. If cediranib dosing is held due to fatigue for longer than 2 weeks, advice should be sought from the CI (via contact with the CTR) before restarting cediranib.

| **Severity** | **Recommended action** |
| --- | --- |
| **Grade 1** (relieved by rest) | Continue cediranib dosing. |
| **Grade 2** (not relieved by rest, affecting instrumental ADLs) | Hold cediranib dosing for at least 48 hours and until improved to ≤ grade 1. Check TFTs. If recovery takes ≤ 1 week restart at current dose level. If recovery takes longer than 1 week consider 5 days on, 2 days off dosing at current dose or reduce to next dose level. |
| **Grade 3** (not relieved by rest, affecting self-care ADLs) | Hold cediranib dosing for at least 48 hours and until improved to ≤ grade 1. Check TFTs. At recovery, restart cediranib but use 5 days on, 2 days off dosing at current dose or reduce to next dose level. |

Table 6: Cediranib fatigue management guidelines.

##### Abnormal Thyroid Function Tests

Cediranib treatment can cause hypothyroidism and up to 25 % of patients develop abnormal TFTs during cediranib therapy. In most cases, these are asymptomatic and do not require intervention. TFTs should be checked in all participants with ≥ grade 2 fatigue and thyroid replacement therapy initiated for symptomatic hypothyroidism as per standard local practice. Cediranib dosing should not be altered for hypothyroidism although treatment may be held until resolution of symptoms at the discretion of the local investigator.

##### Other cediranib-related toxicities

Other expected adverse events for cediranib include nausea/ vomiting, dysphonia (hoarseness), oral mucositis and thrombocytopenia. Note that thrombocytopenia is listed on CTCAE version 4.03 as ‘platelet count decreased’. These are generally mild and overlap with the expected toxicities for olaparib and paclitaxel. Investigators should follow specific guidance for olaparib and paclitaxel dosing. If in the investigator’s opinion, cediranib has made a significant contribution to a particular AE, then general guidance for cediranib dosing is given in Table 7.

Guidance can be sought from the CTR if required and advice should be obtained from the CI (via the CTR) if cediranib dosing is held for longer than 2 weeks due to toxicity.

| **Severity** | **Recommended action** |
| --- | --- |
| **Grade 1** | Symptomatic care. Continue cediranib dosing. |
| **Grade 2** | Symptomatic care. Hold cediranib dosing if toxicity does not recover to ≤G1 within 48 hours. On recovery, resume dosing at current dose level. If toxicity recurs, consider 5 days on, 2 days off dosing at current dose or reduce to next dose level. |
| **Grade 3** | Symptomatic care. Hold cediranib dosing if toxicity does not recover to ≤G1 within 48 hours. On recovery, restart cediranib but use 5 days on, 2 days off dosing at current dose or reduce to next dose level. |
| **Grade 4** | Discontinue Cediranib permanently. |

Table 7: Cediranib guidance for the management of other related toxicities.

### Olaparib dose modifications

Olaparib will be administered concurrently with cediranib in participants randomised to Arm 3. As these drugs have a partially overlapping toxicity spectrum, investigators should evaluate carefully which drug is the likely cause of adverse events on treatment and refer to the guidance in both this section and that in section 11.5.2 when deciding management strategy.

The dosing levels for olaparib are as follows:

- Starting dose: 300 mg twice daily
- Dose Level 1: 250 mg twice daily
- Dose Level 2: 200 mg twice daily
- Dose Level 3: STOP

#### Management of olaparib-related toxicities

The most frequently observed adverse reactions across clinical trials in patients receiving olaparib monotherapy (≥ 10 %) were nausea, vomiting, diarrhoea, dyspepsia, fatigue, headache, dysgeusia, decreased appetite, dizziness, anaemia, neutropenia, lymphopaenia, cough, mean corpuscular volume elevation and increase in creatinine. For the most part these are CTCAE grade 1 or 2 and do not require treatment discontinuation.

Specific guidance for management of key toxicities is given below:

##### Haematological toxicities

Anaemia and other haematological toxicities are generally low grade (CTCAE grade 1 or 2). Pre-treatment testing, followed by monthly monitoring of complete blood counts is mandatory during the trial and clinically significant changes during treatment in any parameter should be managed as described below. Common treatable causes of anaemia should be excluded in all cases of anaemia including iron, Vitamin B12 and folate deficiencies and hypothyroidism.

In the event of Grade 3 or Grade 4 haematological toxicity, complete blood counts should be checked at least weekly until recovery to Grade 1 or better. Table 8 shows the guidelines for managing haematological toxicities in participants taking olaparib.

| **Event** | **Toxicity** | **Recommended action** |
| --- | --- | --- |
| Anaemia | Grade 2: 80 < Hb ≤ 90 g/l | PI to investigate and manage as they deem appropriate. Treatment options include interruption of olaparib for up to 4 weeks, or olaparib dose reduction, taking into account previous history of anaemia.  After 4 weeks, if Hb remains low (80 < Hb ≤ 90 g/l), dose interrupt (up to total interruption of 4 weeks) until Hb ≥ 90 g/l. Upon recovery (Hb > 90 g/l) consider reducing olaparib to next available dose level.  Cediranib can be continued at the PI’s discretion. |
|  | Grade 3 or worse: Hb < 80 g/l | Interrupt olaparib treatment for up to 4 weeks* until Hb ≥ 90 g/l. Give supportive management including transfusion* and initiate appropriate haematological testing.  Upon recovery to Hb ≥ 90 g/l, olaparib should be restarted at the next available dose level. If Hb decreases again, immediately reduce to next available dose level.  Cediranib can be continued at the PI’s discretion. |
| Neutropenia | Grade 3 or 4 (ANC <1.0 x10^9^/l) | First occurrence: interrupt olaparib treatment for up to 4 weeks* until recovered to CTCAE grade 1 or better (ANC ≥ 1.5 x 10^9^/l).  Repeat occurrence: interrupt olaparib treatment for up to 4 weeks until recovered to CTCAE grade 1 or better (ANC ≥ 1.5 x 10^9^/l). Recommence olaparib at the next available dose level.  Cediranib can be continued at the PI’s discretion.  Primary prophylaxis with G-CSF is not recommended. |
| Febrile neutropenia | Grade 3 or worse (ANC <1.0 x10^9^/l and temperature > 38^o^C) | Interrupt olaparib treatment until neutrophil recovery to CTCAE grade 1 or better (ANC ≥ 1.5 x 10^9^/l), then dose reduce and recommence olaparib at the next available dose level.  Prophylaxis with G-CSF can be used in accordance with local guidelines. G-CSF should not be used within 24 hours of the last dose of olaparib. Growth factor support should be stopped at least 24 hours before restarting olaparib (7 days for pegylated G-CSF).  Cediranib can be continued at the PI’s discretion. |
| Thrombocytopenia | Grade 3 or worse (platelets < 50 x 10^9^/l) | Interrupt treatment with both olaparib and cediranib for up to 4 weeks* until recovery to CTCAE grade 1 or better (platelets > 75 x 10^9^/l).  If recovery (platelets > 75 x 10^9^/l) occurs within 2 weeks, and thrombocytopenia was not worse than grade 3 (platelets > 25 x 10^9^/l), resume cediranib and olaparib dosing at current levels.  If CTCAE grade 4 thrombocytopenia (platelets < 25 x 10^9^/l) had occurred, or recovery takes longer than 2 weeks, then once recovered, recommence olaparib at the next available dose level. Cediranib dosing can be resumed at the same level. |
| * If a participant has a ≥2-week interruption in olaparib due to CTCAE grade 3 or worse anaemia, neutropenia (CTCAE lists as decrease in neutrophil count) or thrombocytopenia (CTCAE lists as decrease in platelet count), or if they develop blood/platelet dependence then refer to the information below on prolonged haematological toxicities. | | |

Table 8: Management of olaparib-associated haematological toxicities.

If a participant develops prolonged haematological toxicity such as:

- ≥2-week interruption in olaparib treatment due to CTCAE grade 3 or worse anaemia and/or development of blood transfusion dependence.
- ≥2-week interruption in olaparib treatment due to CTCAE grade 3 or worse neutropenia (ANC < 1.0 x 10^9^/l).
- ≥2-week interruption in olaparib treatment due to CTCAE grade 3 or worse thrombocytopenia (CTCAE lists as decrease in platelet count) and/or development of platelet transfusion dependence (platelets < 50 x 10^9^/l).

Check weekly differential blood counts including reticulocytes and peripheral blood smear. If any blood parameters remain clinically abnormal after 4 weeks of dose interruption, the participant should be referred to a haematologist for further investigations. Bone marrow analysis and/or blood cytogenetic analysis to exclude MDS should also be considered at this stage. Study treatment should be discontinued if haematological toxicities requiring dose interruption do not recover to CTCAE grade 1 or better within 4 weeks of dose interruption.

Development of confirmed MDS, AML or other clonal blood disorder should be reported as an SAE for as long as the participant is being followed-up (see section 14.2) and full reports must be provided by the investigator. Olaparib treatment will be discontinued if a diagnosis of MDS and/or AML is confirmed. If olaparib is discontinued due to MDS or AML, then cediranib treatment may be continued only if the PI considers the cediranib is giving ongoing clinical benefit and the risk benefit remains favourable, and if the CI has provided written approval (see section 11.5). If a clonal blood disorder other than MDS/AML is diagnosed during olaparib treatment, the CI should be contacted via the CTR for advice regarding continuation of olaparib and/or cediranib.

##### Gastrointestinal (GI) toxicities

GI toxicities are frequently reported with olaparib therapy and are generally low grade (CTCAE grade 1 or 2) and intermittent and can be managed by dose interruption for up to 4 weeks and/or concomitant medicinal products (e.g., single agent antiemetic therapy). Routine antiemetic prophylaxis is not required but appropriate therapy should be provided at the first onset of nausea or vomiting and as required thereafter, in accordance with local treatment practice guidelines.

In the event of diarrhoea, please refer to the cediranib management guidelines (section 11.5.2.1.4).

In the event of repeated frequent episodes of CTCAE grade 2 GI toxicities related to olaparib, a dose reduction to the next available level should be considered.

In the event of CTCAE grade 3 GI toxicity, considered related to olaparib and not resolving to ≤ CTCAE grade 1 within 48 hours with supportive care, olaparib dosing should be held until recovery to grade 1 or better. Olaparib can then subsequently be restarted at a reduced dose (see section 11.5.3).

##### Management of new or worsening respiratory symptoms

Pneumonitis has been reported in a small number of patients receiving olaparib, and some reports have been fatal. Pneumonitis had no consistent clinical pattern and was confounded by a number of predisposing factors (cancer and/or metastases in lungs, underlying pulmonary disease, smoking history, and/or previous chemotherapy and radiotherapy). If participants present with new or worsening respiratory symptoms such as dyspnoea, cough and fever, or a radiological abnormality occurs, olaparib should be interrupted and prompt investigation initiated, for example with high resolution CT scan of the chest. Following investigation, if no evidence of abnormality is observed on CT imaging and symptoms resolve, then olaparib treatment can be restarted, if deemed appropriate by the investigator.

If any grade of pneumonitis is confirmed, olaparib should be discontinued permanently and the participant treated appropriately. Pneumonitis grade 2 or worse should be reported as an SAE for as long as the participant is being followed-up (see section 14.2) and full reports must be provided by the investigator.

If olaparib is discontinued due to pneumonitis, then cediranib treatment may be continued only if the PI considers the cediranib is giving ongoing clinical benefit and the risk benefit remains favourable, and if the CI has provided written approval (see section11.5).

##### Management of abnormal renal function during olaparib therapy

A dose reduction to 200 mg twice daily is recommended for participants who develop moderate renal impairment (creatinine clearance of between 31 and 50 ml/min) for any reason during the course of the trial. If the participant is already on a dose of 200 mg twice daily, then it is not necessary to reduce their olaparib dosage further.

Olaparib has not been studied in patients with severe renal impairment (creatinine clearance ≤ 30 ml/min) or end-stage renal disease; if participants develop severe impairment or end stage disease it is recommended that olaparib be discontinued.

If olaparib is discontinued due to abnormal renal function, then cediranib will also be discontinued.

##### Management of other toxicities considered related to olaparib

A flexible approach will be taken to allow participants to gain maximum benefit from treatment. Any toxicity observed during the course of the trial that is deemed related to olaparib can be managed by olaparib treatment interruption if deemed appropriate by the investigator. Trial treatment must be interrupted until the participant recovers completely or the toxicity reverts to CTCAE grade 1 or better. Repeat dose interruptions for CTCAE grade 2 toxicities are allowed as required, for a maximum of 4 weeks on each occasion. If an interruption of longer than 4 weeks is required then the participant will be withdrawn from further treatment with both olaparib and cediranib, unless specific approval has been granted by the CIs (via the CTR) for cediranib to continue as a single agent. See Table 9 and section 11.5 for further guidance.

| **Severity** | **Recommended action** |
| --- | --- |
| **Grade 1** | Symptomatic care. Continue olaparib dosing. |
| **Grade 2** | Symptomatic care. Hold olaparib dosing if toxicity does not recover to ≤ grade 1 within 48 hours. On recovery resume dosing at current dose level. If toxicity recurs, continue current dose or reduce to next dose level at investigator’s discretion. |
| **Grade 3** | Symptomatic care. Hold olaparib dosing if toxicity does not recover to ≤ grade1 within 48 hours. On recovery, restart olaparib but reduce to next dose level. |
| **Grade 4** | Discontinue olaparib (cediranib must also be discontinued unless written approval has been granted by the CI (via the CTR) for cediranib to continue). |

Table 9: Olaparib guidance for the management of other related toxicities.

## Pre-medication

In anticipation of diarrhoea, participants allocated to receive cediranib (arms 2 and 3) will be prescribed loperamide in advance of commencing cediranib. The loperamide can then be taken without delay if required (see section 11.5.2.1.4).

## Management of an overdose

The trial drugs must only be used in accordance with the dosing recommendations in this protocol. Any dose or frequency of dosing that exceeds the dosing regimen specified in this protocol must be reported as an overdose.

In the event of an overdose, the physician will administer the most appropriate treatment for the participant and treat any ARs associated with the overdose symptomatically.

Overdoses must be initially reported to the CTR within 24 hours of the research team becoming aware of the overdose. To report an overdose, the research team must complete the form for reporting overdoses, and email or fax a copy to the CTR Safety Team (see contact details in section 0). The local research team is responsible for the completion of this form and the PI (or another delegated medically qualified doctor from the trial team) is required to sign the form. The local research team is required to provide the CTR with any requested follow-up information as soon as possible. The CTR will report all overdoses to AstraZeneca within 24 hours of becoming aware of the overdose.

An overdose is not an AE and may not result in any noticeable effect on the participant. However, if the participant experiences a SAE that the PI considers may be causally related to an overdose, then this must be clearly stated on the SAE form (see section 14 for SAE reporting procedures).

### Overdose of cediranib

There is no specific treatment for cediranib overdose. In cases of suspected overdose, cediranib should be interrupted, blood pressure monitored and appropriate supportive care instituted. If the PI wishes to recommence cediranib treatment they must first obtain written permission from the CI (via the CTR).

### Overdose of olaparib

There is no specific treatment for overdose of olaparib and possible symptoms are not established. In the event of a suspected overdose with olaparib, olaparib should be interrupted and appropriate supportive care should be instituted. If the PI wishes to recommence olaparib treatment, they must first obtain written permission from the CI (via the CTR).

## Prohibited medications and interaction with other drugs

### Cediranib-restricted concomitant medications

Use of cediranib should be avoided with the following potent inducers of UCT/P-gp (e.g., rifampicin, carbamazepine, phenobarbital, phenytoin and St. John’s Wort). Cediranib is not an inhibitor of MDR1, but it has a low potential to inhibit BCRP. The possibility that cediranib may induce GI CYP3A and UGT enzymes cannot be excluded. Co-administration of a CYP3A4/P-gp inhibitor with cediranib does not require a priori dose adjustment.

### Olaparib-restricted concomitant medications

#### Drugs that modulate CYP3A4 enzyme activity, herbal supplements and grapefruit/Seville oranges

Olaparib can inhibit CYP3A4 and uridine di-phosphoglucuronosyl transferase 1A1 (UGT1A1) enzyme *in vitro*. These findings suggest that olaparib has the potential to cause clinically significant interactions with other CYP3A4 substrates or UGT1A1 substrates in the liver or GI tract. *In vitro* data have shown that the principal enzyme responsible for the formation of the three main metabolites of olaparib is CYP3A4 and consequently, participants should avoid concomitant use of drugs known to modulate CYP3A4 enzyme activity, herbal supplements and/or ingestion of foods such as grapefruit juice or Seville oranges (often found in marmalade), from the time they enter the screening period until 30 days after the last dose of trial medication.

Specific guidance on restricted medication with olaparib is given below:

**Strong or Moderate CYP3A inhibitors:** Concomitant use of known strong CYP3A inhibitors (e.g., itraconazole, telithromycin, clarithromycin, boosted protease inhibitors, indinavir, saquinavir, nelfinavir, boceprevir, telaprevir) or moderate CYP3A inhibitors (ciprofloxacin, erythromycin, diltiazem, fluconazole, verapamil) with olaparib should be avoided where possible.

If there is no suitable alternative concomitant medication, then olaparib should be stopped for the period of concomitant administration. Olaparib should only be restarted once the CYP3A inhibitor has been discontinued and after discussion with the CI (via the CTR). A maximum of 4 weeks off olaparib will be permitted in this circumstance.

**Strong or Moderate CYP3A inducers:** Concomitant use of strong (e.g., phenobarbital, phenytoin, rifampicin, rifabutin, rifapentine, carbamazepine, nevirapine, enzalutamide and St John’s Wort) and moderate inducers (eg. bosentan, efavirenz, modafinil) of CYP3A with olaparib should be avoided where possible. The use of any strong or moderate CYP3A inducers may diminish the clinical efficacy of olaparib. If the use of any strong or moderate CYP3A inducers are considered necessary for the participant’s safety and welfare this will be discussed with the CI (via the CTR) on a case-by-case basis. Participants should be monitored carefully for any reduction in the efficacy of olaparib.

**P-gp inhibitors:** It is possible that co-administration of P-gp inhibitors (e.g., amiodarone, azithromycin) may increase exposure to olaparib. Caution should therefore be observed.

**Effect of olaparib on other drugs:**

Based on limited *in vitro* data, olaparib may increase the exposure to substrates of CYP3A4, P-gp, OATP1B1, OCT1, OCT2, OAT3, MATE1 and MATE2K.

Based on limited *in vitro* data, olaparib may reduce the exposure to substrates of CYP3A4, CYP1A2, 2B6, 2C9, 2C19 and P-gp.

The efficacy of hormonal contraceptives may be reduced if co administered with olaparib.

Caution should therefore be observed if substrates of these isoenzymes or transporter proteins are co-administered. In particular, caution should be exercised if olaparib is administered in combination with any statin.

Examples of substrates include:

CYP3A4 hormonal contraceptive, simvastatin, cisapride, cyclosporine, ergot alkaloids, fentanyl, pimozide, sirolimus, tacrolimus and quetiapine

CYP1A2 duloxetine, melatonin

CYP2B6 bupropion, efavirenz

CYP2C9 warfarin

CYP2C19 lansoprazole, omeprazole, S-mephenytoin

P-gp—simvastatin, pravastatin, digoxin, dabigatran, colchicine

OATP1B1 bosentan, glibenclamide, repaglinide, statins and valsartan

OCT1, MATE1, MATE2K—metformin

OCT2 serum creatinine

OAT3 furosemide, methotrexate

UGT1A1 irinotecan, nintedanib, ezetimibe, raltegravir or buprenorphine

## Permitted concomitant medications

Concomitant medication may be given if clinically mandated. Details (including doses, frequency, route and start and stop dates) of the trial IMP and the concomitant medication will be recorded in the participant’s medical records and relevant details entered into the CRF. Recording of concomitant medication should continue until 30 days after the last dose of IMP.

## Trial restrictions

### Special warning and precautions/ restrictions during the study for olaparib:

**Haematological**: Participants should not start olaparib until they have recovered from the myelosuppressive effects of prior cytotoxic therapy. In a small number of patients who received olaparib, MDS and AML have been reported—usually in patients with germline BRCA gene mutations and in whom there were additional haematological risk factors such as prior cytotoxic therapy or radiation treatment.

**Pneumonitis**: Pneumonitis has been reported in a small number of patients, without a clear underlying cause. If participants present with shortness of breath, worsening respiratory symptoms or with radiological abnormalities on imaging of the chest, then olaparib should be stopped and prompt investigation initiated. If pneumonitis is confirmed olaparib should be stopped and appropriate treatment sought.

Pneumonitis grade 2 or worse should be reported as an SAE for as long as the participant is being followed-up (see section 14.2) and full reports must be provided by the investigator.

### Special warnings and precautions for cediranib:

**Elderly (>65 years)**: No adjustment in starting dose is required for elderly participants. There are limited data in patients aged 75 or over.

**Renal impairment**: No dose adjustment is recommended for participants with mild (creatinine clearance ≥60 mL/min to <90 mL/min) or moderate (creatinine clearance ≥30 mL/min to <60 mL/min) renal impairment, based on population pharmacokinetic analysis. No data are available in patients with severe (creatinine clearance <30 mL/min) renal impairment or patients on dialysis.

**Hepatic impairment**: No dose adjustment is required in participants with mild or moderate hepatic impairment (Child-Pugh class A or B). No data are available in patients with severe Hepatic impairment.

Cediranib is not known to alter blood glucose levels.

### General warnings and precautions for all treatment arms:

**Pregnancy and Teratogenicity**: All IMPs should be avoided.

**Anticoagulant Therapy**: Participants who are taking coumarin-type anticoagulants (such as warfarin) may participate in this trial; however, it is recommended that prothrombin time (international normalised ratio (INR) and activated partial thromboplastin time (APTT)) be monitored carefully at least once per week for the first month, then monthly if the INR is stable.

Subcutaneous heparin is permitted without the need for additional monitoring. PIs should consider on a per-participant basis whether to convert therapeutic anticoagulation from coumarin-type to low molecular weight heparin while participants are receiving trial therapy.

**Other drugs**: Ondansetron and related drugs which impact the QTc interval should be used with caution.

**Surgery**: Prior to planned surgery; olaparib should be stopped 3 days before, and cediranib should be stopped 14 days before. No stoppage of olaparib is required for a needle biopsy procedure or radiologically-guided drain insertion. However, cediranib should be stopped 3 days before these procedures, and can be restarted 2 days after, provided there are no haemorrhagic complications. In the event of unplanned surgery, management will be dictated by the participant’s clinical needs.

Cediranib and olaparib can be re-started a minimum of 14 days after surgery provided that the participant has recovered from surgery and the wound has healed satisfactorily. Note that if surgery was performed due to disease progression, then the participant will no longer receive trial treatment. If a participant experiences wound healing complications whilst on trial treatment, cediranib dosing should be interrupted until the wound is fully healed. It is anticipated that cediranib will be restarted within 28 days of surgery. If the surgical wound has not fully healed by 28 days, further delays in commencing cediranib will be considered on a case-by-case basis on discussion with the trial CI (via the CTR).

**Radiotherapy**: Palliative radiotherapy is allowed for pre-existing small areas of painful metastases that cannot be managed with local or systemic analgesics as long as no evidence of disease progression is present. No other radiotherapy is allowed during trial treatment. Olaparib should be discontinued for a minimum of 3 days before a participant undergoes radiation treatment. Olaparib should be restarted within 4 weeks as long as any bone marrow toxicity from radiotherapy has recovered to grade 1 or less. Cediranib and paclitaxel may be continued during radiotherapy if the local investigator considers this to be in the best interest of the participant.

**Anti-emetics**: From screening onwards, should a participant develop nausea or vomiting, then these symptoms should be reported as AEs and appropriate treatment of the event given as per local guidelines.

**Prohibited treatments**: The following treatments are not allowed while the participant is on this trial treatment and or during the follow up:

- No other chemotherapy, hormonal therapy or other novel agent is to be permitted during the course of the trial for any participant.
- No other IMP can be administered while the participant is on the trial.
- Live virus and bacterial vaccines should not be administered whilst the participant is receiving trial IMP and during the 30 day follow up period. An increased risk of infection by the administration of live virus and bacterial vaccines has been observed with conventional chemotherapy drugs and the effects with olaparib are unknown.

## Accountability procedures

For drug accountability, pharmacies at participating sites will be provided with log forms from the CTR which will be stored in the PSF. Sites will be trained in the procedures for completing, storing and returning these logs. Sites may be permitted by the CTR to use their local versions of logs if the logs capture all the required information. In this case sites should provide the CTR with a copy of their local logs for the TMF.

A risk-adapted approach to drug accountability, in accordance with the Good Clinical Practice Guide (2012), will be used. The Pharmacy Manual provides full details on the accountability required for each IMP. In brief, paclitaxel requires “low” accountability procedures as it will be sourced from general site pharmacy stock and its off-label use for treating endometrial cancer is established practice. Low accountability does not require shipping receipt or destruction records. Recording of batch numbers/and or expiry dates will be in accordance with local practice for paclitaxel administration. Cediranib and olaparib are unlicensed for use in endometrial cancer and are sourced from trial-specific supplies; they therefore require “high” accountability. High accountability requires full accountability records of receipt, use and return/destruction.

Compliance will be checked as described in section 11.12. If required, trial drug will be destroyed as in section 11.2.3.

## Compliance

Participants will be monitored for compliance and for attendance at protocol-scheduled visits. Participants are highly motivated to be compliant with the drug and visit schedule, and it is very unusual for a participant to be persistently non-compliant. For this reason, there are not a defined number of missed visits or doses which would make the participant be classed as non-compliant. PIs who are concerned about compliance should discuss the circumstances with the CI (via the CTR) on a case-by-case basis to determine if action is necessary.

If participants do not present for a scheduled visit or follow up, the procedure in section 10.2 will be followed.

For participants in arms 1 and 2, the administration of paclitaxel will be recorded in the appropriate sections of the CRF and participant notes.

Participants in arms 2 and 3 will self-administer cediranib and olaparib and will be given clear instructions on how and when to take their trial treatment. They will be instructed to record dates of missed or held doses in their participant diary and to notify trial site personnel of missed doses. At each clinic visit, the participant will be asked to confirm how many drug doses were taken or omitted. Administration and omission of drug doses will be recorded by site staff in the CRF and participant notes. All participants must return their bottle(s) of cediranib and olaparib, preferably at the start of the next treatment cycle. Trial site staff will count the unused tablets on return, and the information will be recorded in the appropriate section of the CRF. After the tablet count has been performed, the remaining tablets will not be returned to the participant but will be retained by the local clinical trial pharmacist for reconciliation. Detailed instructions regarding IMP reconciliation checks will be provided to Pharmacy. If the participant is continuing trial medication, new bottles will be dispensed. At the end of trial treatment, participants must return all containers and any remaining tablets to the research team.

Overall treatment compliance will be assured by review of the information recorded in the CRF, participant diaries and by full reconciliation of the IMP accountability logs.

# Sample Management

Samples collected in the COPELIA trial will NOT be used for stratification or to assign participants to a trial arm. Samples collected will NOT be used for diagnosis or to influence the treatment received by the participant.

Throughout the trial treatment, participants may have blood or other samples taken to monitor the participant’s health and their eligibility to continue with treatment. Such non-research samples will be handled, transported and destroyed as per local procedures for testing patient samples within the NHS framework. Consistent with this, all labs analysing non-research tests will have been assessed and accredited appropriately, for example having Clinical Pathology Accreditation (CPA) or accreditation from the United Kingdom Accreditation Service (www.ukas.com). The handling of such samples will not be discussed in the COPELIA sample handling manual.

Table 10 below shows the schedule of research sample collection in the COPELIA trial. Blood plasma is collected to determine angiomodulatory molecules. Whole blood is collected to determine levels of CTCs, and 3 ml will be immediately frozen for possible future use. Archival tumour tissue will be tested for *POLEm*, MMRd and p53abn.

| Study visit | Trial arms | Sample Type |
| --- | --- | --- |
| Screening* | All arms | Blood plasma |
| Post-randomisation and before or on C1D1 |  |  |
|  | All arms | Blood plasma |
|  | All arms | Whole blood for CTCs |
| C2D1 (or up to 4 days prior) | All arms | Blood plasma |
| C3D1 (or up to 4 days prior) | All arms | Blood plasma |
| 6 and 12 weeks (with CT scan or treatment visit) | All arms (only participants with CTCs on C1D1) | Whole blood for CTCs |
| End of treatment | All arms | Blood plasma |
|  | All arms | Whole blood for freezing |
|  | All arms (only participants with CTCs on C1D1) | Whole blood for CTCs |

Table 10: Schedule of research sample collection in the COPELIA trial.

All participating sites will be trained in the sample handling procedures and will receive a copy of the COPELIA sample handling manual.

For all research samples it is the responsibility of the participating site to ensure that samples are packaged, labelled and transported in accordance with the COPELIA sample handling manual. The procedures described in the sample handling manual have been designed to ensure that samples are received in good condition and in an optimal state for processing. The labelling instructions ensure that all samples comply with the Data Protection Act 2018 and provide researchers with the required information for processing and tracking samples received.

Blood samples and tissue biopsies will be sent to, and processed at, different research centres as described in the COPELIA sample handling manual. For each research centre, an agreement between the Sponsor and the centre will be signed by both parties before initiation of the trial. Samples will only be transported to, processed by, and destroyed by laboratories approved by the Sponsor. All research samples will be processed and stored under approval from a recognised REC and therefore, according to the Human Tissue Act 2004, centres do not need to hold a licence from the Human Tissue Authority (HTA).

The TMG will review which samples remain after analysis and will implement a plan for remaining samples. This plan may involve further testing, storage or destruction, and will always be in accordance with the consent given by participants and legal and regulatory requirements.

- Consent: all participants who provide optional research samples will have consented to future research on their samples, including genetic testing.
- Legal requirement: beyond the REC approval, it is a legal requirement for all relevant material to be stored on HTA-licensed premises.

## Samples for measurement of angiomodulatory molecules in plasma

Blood plasma samples are required for the measurement of angiomodulatory molecules. For each participant, and at required time points (see Table 10), sites will collect 2 x 10 ml of blood into an EDTA collection tube. Initial processing of the samples—spinning to separate blood components, aliquoting and freezing—will be performed at site.

On arrival at the research centre, the samples will be checked, logged and stored at -80°C. No samples will be thawed for analysis until the research centre has received written notice from the Sponsor stating that analysis should be initiated.

Angiogenesis biomarkers will be quantified using commercially available, validated ELISA assays (Backen et al 2009). The following biomarkers will be measured: VEGFA, Ang2, Ang1, Tie2, VEGFR1 and VEGFR2. Participants will have provided informed consent for the analysis of angiogenesis biomarkers generally, and so if new assays for relevant biomarkers become available then, with the agreement of the Sponsor, the research centre may also analyse these. Similarly, if any assay is withdrawn, then this will be communicated to the Sponsor by the research centre, and the assay will not be undertaken. In both cases it would not be necessary to inform the participant or amend the protocol. All changes to the planned sample processing will be documented in the TMF.

It is expected that surplus plasma will remain and this may be analysed in the future, possibly for genetic analysis.

## Whole blood samples for evaluation of circulating tumour cells (CTCs) and future use

Samples will be collected using the CELLSEARCH® platform for the enumeration of CTCs and assessment of gamma-H2AX in captured CTCs. At each time point, as outlined in Table 10, 10ml whole blood will be collected in CellSave® tubes and shipped at ambient temperature within 24 hours of collection. Samples will be analysed by CELLSEARCH® for enumeration of CTCs within 96 hours of sample draw. Note that only participants who have detectable CTCs at baseline will have whole blood taken at later dates for further CTC analysis.

A 3 ml EDTA tube of whole blood will Also be collected from each consenting participant at the end of treatment visit. This sample will be frozen at -80°C at site and periodically shipped to the research centre together with the plasma samples. This sample will be analysed in the future and may include genetic analysis.

The collection and shipping procedures are detailed in the COPELIA sample handling manual.

## Tumour samples for evaluation of *POLEm*, MMRd, p53abn and NSMP

Archival tumour tissue will be tested using genetic sequencing and/or protein expression assays for POLE mutations (POLEm), MMR deficiency (MMRd) and aberrant p53 expression (p53abn). Tumours with POLEm, MMRd and p53abn will be classified as POLEm. Tumours with no POLEm but instead MMRd and p53abn will be classified as MMRd. Tumours with no POLEm or MMR deficiency (referred to as proficient MMR [MMRp]) but instead abnormal p53 expression with be classified as p53abn. Tumours with no POLEm, MMRp and no abnormal p53 expression will be classified as no specific molecular profile (NSMP).

# Trial visits and procedures

The order of visits in the trial can vary depending on which arm the participant is in and whether the participant withdraws or disease progresses, the approximate order is:

1. Screening for eligibility (this may require multiple visits to complete all tests), and registration to the trial
2. Randomisation (may be combined with C1D1 visit)
3. Participants in Arm 3 at participating sites may choose to return for biopsy
4. Treatment associated visits for up to six cycles
   - Arms 1 and 2: visits on D1, 8 and 15 of each cycle*
   - Arm 3: visit on D1 and 15 of each cycle*
5. CT scans at 6 and 12 weeks post-randomisation and then every 12 weeks until disease progression
6. End-of-treatment assessment 30 days after trial treatment has ended (or sooner if required, see section 10.1). If done sooner, then medical notes will be checked for SAEs after 30 days have passed.
7. Monthly assessments in hospital until disease progression
8. After disease progression, hospital visits are not required but remote follow-up is 3-monthly.

*Sites may require participants to attend hospital before the treatment visit for bloods to be taken (bloods to be taken as close to the treatment visit as possible, up to maximum of 4 days).

Telephone assessments are permitted if the participant is unable to attend the hospital site from cycle 3 for participants on Arms 2 & 3 only. Blood, BP and urinalysis assessments can be done locally (GP/local hospital) if the patient is unable to attend the site. BP assessment is important for patients on olaparib and cediranib due to cediranib AEs. Records of all assessments, reports/results and telephone consultations must be updated in the patient notes. If a patient is unable to have bloods, BP and urinalysis assessments contact CTR immediately to discuss if the patient can continue trial treatment.

## Screening for eligibility and registration to the trial

Screening tests to determine the participant’s eligibility will be carried out within 28 days prior to the first dose of treatment. Bloods that are required for treatment decisions will need to be repeated if these have not been performed within 4 days of C1D1 dosing.

Written informed consent will be obtained at the screening visit and before any trial-specific procedures are undertaken.

The procedures undertaken and data recorded at the screening visit(s) are:

- Urine analysis for protein—note that a re-test urine dipstick at least a week later will be required if the first dipstick is ≥1+. Results from a urine dipstick taken in clinic before the participant has consented to the trial may be used if it was taken within 28 days prior to the first dose of treatment, and the result has not been superseded by a urine dipstick showing a higher level of protein.
- 12-lead ECG—QTc should be calculated using the Fridericia formula. If the first test does not show a QTc > 470 ms then a retest is not required. If the first test does show a QTc > 470 ms then a second test should be done within a 24 hour period.
- ECOG, height, weight, age (determined from month and year of birth)
- Pregnancy test for any participant of child bearing potential
- Vital signs including blood pressure and temperature
- Full blood count (haemoglobin, neutrophils, platelets), biochemistry (sodium, potassium, urea, serum creatinine, calcium, phosphate, albumin, bilirubin, ALT or AST, alkaline phosphatase (ALP), gamma-glytamyl transpeptidase (GGT), lactate dehydrogenase (LDH), TFTs (thyroid stimulating hormone (TSH), T4), CA125, Coagulation (INR, activated partial thromboplastin time (APTT))
- Calculated GFR
- QL questionnaires: these questionnaires will be collected monthly until the end-of-treatment assessment using the validated EORTC questionnaires- QLQ-c30 and the endometrial specific subscale EN24.
- Medical history including histopathological diagnosis, histologic grade and FIGO stage at diagnosis
- Prior cancer treatment: chemotherapy, radiotherapy, surgery, tumour embolisation.
- Prior exposure to an IMP
- Concomitant medications
- Radiological assessment of disease by CT and reported according to RECIST v1.1
- Optional plasma sample for translational analysis
- Registration of participant to obtain screening ID number (see section 9.5.1)

## Randomisation

Before randomisation the inclusion and exclusion criteria must be reviewed in the CRF. To be eligible to enter the trial, all participants must meet all of the inclusion criteria and none of the exclusion criteria and this must be confirmed by a medically qualified doctor on the delegation log. Participants will then be randomised in accordance with section 9.5.2. It is not a requirement for the participant to be present for the randomisation process.

## Treatment associated visits for up to six cycles

See section 13.8 for the timetable of assessments. Treatment associated visits are planned for the initial six-cycles of treatment, however, if the participant has disease progression, then treatment and these associated visits will stop. If the participant completes these visits without disease progression, then further monthly assessments will be undertaken (see section 13.6).

## CT scans

Participants will have CT scans reported according to RECIST v1.1 until disease progression. CT scans are planned for 6 and 12 weeks post-randomisation and then every 12 weeks.

The protocol will result in participants receiving up to two extra CT scans of the chest, abdomen and pelvis in excess of standard of care. While ionizing radiation is associated with risks of second malignancy, the patient group recruited to this trial have advanced recurrent endometrial cancer and thus the risks to this population are considered minimal.

## End-of-treatment assessment

The main purpose of the end-of-treatment assessment is to monitor safety, and this includes identifying whether any SAEs have occurred since treatment ended.

The end-of-treatment assessment involves all of the assessments performed on D1 of each cycle, with the exceptions that:

- 12-lead ECG, as per the screening procedure is collected
- a review of the IMP administered in the previous cycle is not collected
- an optional plasma sample for translational analysis may be collected
- an optional whole blood sample for future analysis may be collected
- an optional whole blood sample may be taken from participants who had detectable CTCs on cycle 1, day 1.

It is necessary for the toxicity assessment to cover 30 days since trial treatment ended for the identification of SAEs. In some cases, the end-of-treatment assessment may need to be done sooner than 30 days after trial treatment, for example if a new treatment is being started (see section 10.1). In this case the site will review the participant’s medical notes after 30 days to complete the toxicity assessment section of the CRF.

End-of-treatment assessments will be completed for any participants still on trial medication at the end of the trial.

## Monthly assessments in hospital until disease progression

Participants on **all arms** who have not experienced disease progression will attend hospital every 28 days for clinical assessments. These monthly assessments involve all of the assessments performed on D1 of each cycle. In a circumstance where a person ends trial treatment before disease progression then the monthly assessments (and 12-weekly CT scans, see section 13.5) will continue beyond the end-of-treatment assessment above.

## Remote follow-up: 3-monthly

After disease progression, hospital visits are not required but remote follow-up will be 3-monthly. For remote follow-up, sites will check the medical notes they hold for that participant. If the information required is not available in these notes, then the site may telephone either the participant directly or their GP for information. Remote follow-up will stop if the participant has died or the trial ends.

## Schedule of Assessments

The following schedule tables show the planned assessments for each arm. The tables refer to this key:

a) CT scan- 3 area- performed at 6 weeks, 12 weeks and then every 12 weeks until disease progression.

b) Blood pressure, pulse and temperature.

c) Biochemistry (sodium, potassium, urea, serum creatinine, calcium, phosphate, albumin, bilirubin, ALT or AST, alkaline phosphatase (ALP), gamma-glytamyl transpeptidase (GGT), lactate dehydrogenase (LDH), TFTs (thyroid stimulating hormone (TSH), T4), CA125, Coagulation (INR, activated partial thromboplastin time (APTT)).

d) FBC (haemoglobin, neutrophils, platelets).

e) Cediranib or olaparib may be dispensed mid-cycle if dose reduction required.

f) Whole blood samples are only taken from participants who had the presence of CTCs confirmed in their initial sample. Whole blood samples are taken only at 6 and 12 weeks (not recurring 12-weekly CT scans). The whole blood sample can be taken at the 6- and 12-weeks treatment visit, or the CT scan visit.

The acceptable time windows for the trial events and assessments are:

- Hospital visits, D 1, 8 and 15: +/- 2 days (blood tests ≤ 4 days prior)
- CT scans: +/- 1 week
- Monthly assessments: +/- 1 week
- End-of-treatment assessment: Min 30 days for toxicity assessment
- Remote follow-up +/- 1 month


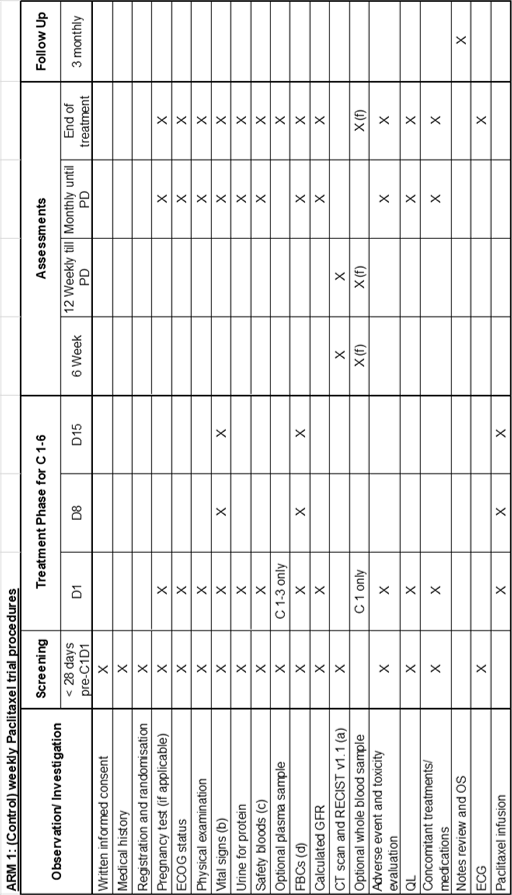

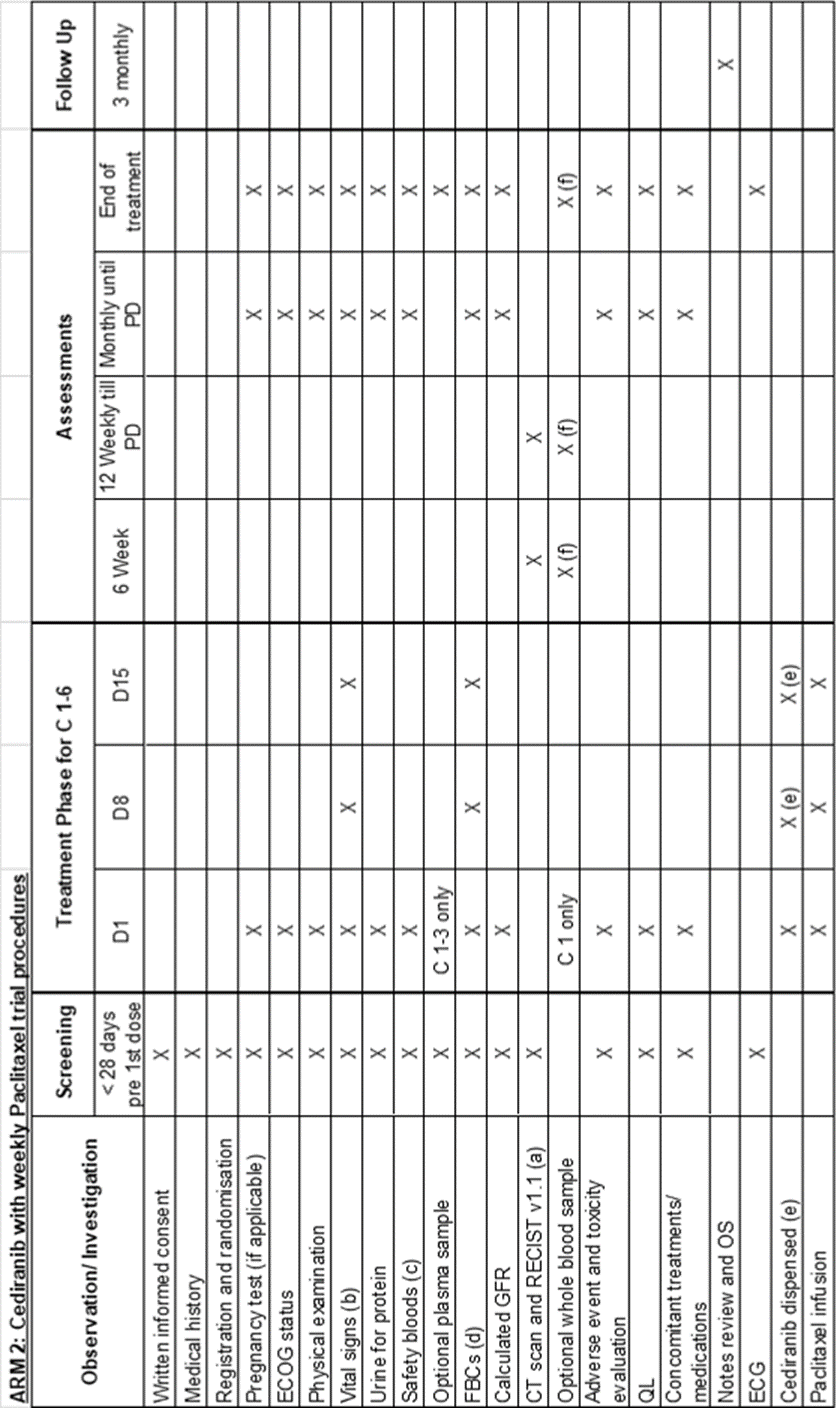


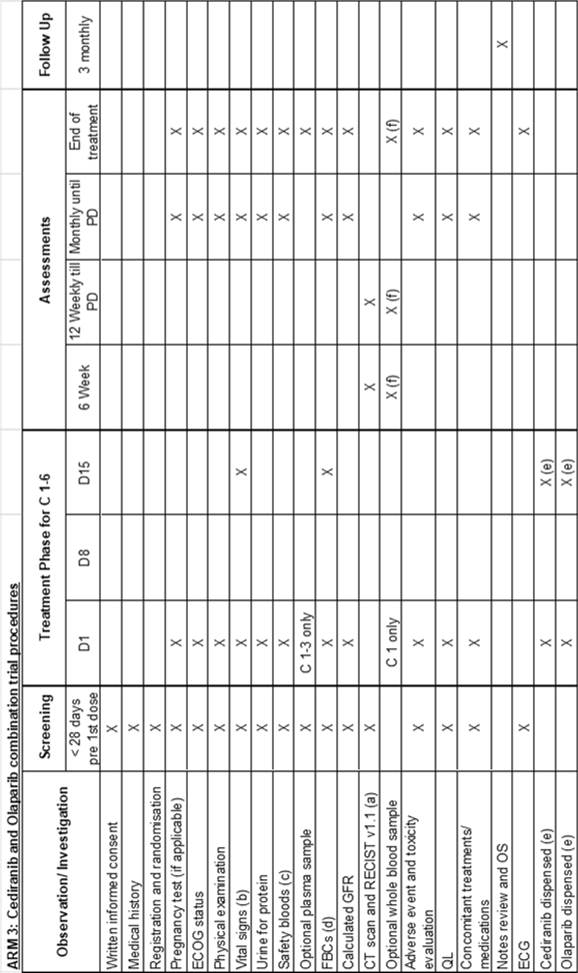


## Follow-up

Follow-up beyond the initial six cycles of treatment involves hospital visits until disease progression, withdrawal from follow-up (or participant becomes lost to follow-up), participant death or the end of the trial. These hospital trial visits involve monthly assessments and 12-weekly CT scans.

Participants will have an end-of-treatment hospital visit 30 days after their last dose of trial drug to monitor for adverse events. If the participant is still taking trial drug when the trial ends then the end-of-treatment hospital visit will happen at this point, even if the participant continues to receive drug beyond the trial.

With the possible exception of the end-of-treatment assessment, there will be no more hospital based trial assessments after disease progression. After discovery of disease progression, participants will be remotely followed up every 3 months.

# Pharmacovigilance

The PI is responsible for ensuring that all site staff involved in this trial are familiar with the content of this section.

All SAEs must be reported immediately (and within 24 hours of knowledge of the event) by the PI at the participating site to the CTR Safety Team (see contact details in section 0), unless the SAE is specified as not requiring immediate reporting (see section 14.2). This includes SAEs related to IMPs, nIMPs and trial procedures.

## Definitions

| Term | Definition |
| --- | --- |
| Adverse Event (AE) | Any untoward medical occurrence in a participant or clinical trial participant administered a medicinal product and which are not necessarily caused by or related to that product |
| Adverse Reaction (AR) | Any untoward and unintended response in a clinical trial participant to an investigational medicinal product which is related to any dose administered to that participant |
| Serious Adverse Event (SAE) | Any adverse event that -   - Results in death - Is life-threatening* - Required hospitalisation or prolongation of existing hospitalisation** - Results in persistent or significant disability or incapacity - Consists of a congenital anomaly or birth defect - Other medically important condition*** |
| Serious Adverse Reactions (SARs) | Any SAE occurring in a clinical trial participant for which there is a reasonable possibility that it is **related** to the IMP at any dose administered. |
| Suspected Unexpected Serious Adverse Reactions (SUSARs) | A SAR, the nature and severity of which is not consistent with the Reference Safety Information (RSI) for the IMP. |

***Note:** The term ‘life-threatening’ in the definition of serious refers to an event in which the trial participant was at risk of death at the time of the event or it is suspected that used or continued used of the product would result in the subjects death; it does not refer to an event which hypothetically might have caused death if it were more severe.

****Note:** Hospitalisation is defined as an inpatient admission, regardless of the length of stay, even if the hospitalisation is a precautionary measure for continued observation. Pre-planned hospitalisation e.g., for pre-existing conditions which have not worsened, or elective procedures, do not constitute an SAE.

*****Note:** other events that may not result in death, are not life-threatening, or do not require hospitalisation, may be considered as an SAE when, based upon appropriate medical judgement, the event may jeopardise the participant and may require medical or surgical intervention to prevent one of the outcomes listed above. Also see section 14.2.

## Trial Specific SAE Reporting requirements

In addition to the SAE reporting requirements above, for the purposes of this trial, the following events will also be considered SAEs and must be captured on the SAE form and reported to the CTR within 24 hours of knowledge of the event—these events should be reported as SAEs for the duration of the study, including when the participant is in follow up:

• Development of MDS, AML or other clonal blood disorder (AML is listed on CTCAE version 4.03 as ‘leukemia secondary to oncology chemotherapy’)

• Pneumonitis grade 2 or worse

• New malignancies

• SARs (as determined by the investigator)

For the purposes of this trial the following events do not require reporting as SAEs:

• Hospitalisation due to disease progression

• Life threatening events due to disease progression

• Death due to disease progression

• Elective hospitalisation and surgery for treatment of disease

• Elective hospitalisation to simplify treatment or trial procedures

• Admissions for palliative care

**These should be completed in the participant’s notes and on the relevant toxicities CRF page and forwarded to the CTR in the normal timeframes for CRFs.** Pre-existing conditions do not qualify as adverse events unless they worsen.

## Causality

Causal relationship will be assessed for IMPs, other trial treatments (nIMPs) and procedures:

| **IMPs:** Paclitaxel, Cediranib, Olaparib  **nIMPs:** All participants receiving cediranib (Arms 2 and 3) will be prescribed loperamide in advance of expected diarrhoea. Other nIMPs to reduce side-effects may be given.  **Procedures:** Participants allocated to Arm 3, and being treated at participating hospitals, may have a tissue biopsy. In some cases, participants may receive palliative radiotherapy. |
| --- |

The PI (or another delegated medically qualified doctor from the trial team) will assess each SAE to determine causality. In addition, the CI (or another medically qualified doctor from the TMG) may provide an assessment of causality. The PI’s (or delegate’s) causality decision will not be influenced by the Sponsor (CI or their delegate). If the causality assessments differ then the most conservative approach will be taken for regulatory reporting. The causality assessment given by the PI (or delegate) cannot be downgraded by the CI (or delegate), and the PI (or delegate) will not be put under any pressure to change their assessment. In the case of disagreement both opinions will be fully documented.

| Relationship | Description | Reasonable possibility that the SAE may have been caused by the IMP, nIMP or trial procedure? |
| --- | --- | --- |
| Unrelated | There is no evidence of any causal relationship with the IMP, nIMP or trial procedure. | No |
| Unlikely | There is little evidence to suggest there is a causal relationship with the IMP, nIMP or trial procedure (e.g., the event did not occur within a reasonable time after administration of the trial medication). There is another reasonable explanation for the event (e.g., the participant’s clinical condition, other concomitant treatment). | No |
| Possible | There is some evidence to suggest a causal relationship with the IMP, nIMP or trial procedure (e.g., because the event occurs within a reasonable time after administration of the trial medication). However, the influence of other factors may have contributed to the event (e.g., the participant’s clinical condition, other concomitant treatments). | Yes |
| Probable | There is evidence to suggest a causal relationship and the influence of other factors is unlikely. | Yes |
| Definite | There is clear evidence to suggest a causal relationship and other possible contributing factors can be ruled out. | Yes |

Table 11: Definitions of causality for SAE assessments.

## Expectedness

The Chief Investigator (or another delegated appropriately qualified individual) will assess each SAR to perform the assessment of expectedness.

The expectedness assessment will be made with reference to the current Reference Safety Information (RSI) for each IMP (see Table 12). RSI will be reviewed regularly according to CTR procedures. Expectedness decisions must be based purely on the content of the RSI; other factors such as the participant population and participant history should not be taken into account. Expectedness is not related to what is an anticipated event within a particular disease.

SARs which add significant information on specificity or severity of a known, already documented adverse event constitute unexpected events. For example, an event more specific or more severe than that described in the RSI is considered unexpected.

| IMP | RSI to be used for expectedness assessment | Relevant section of RSI to be used for expectedness assessment |
| --- | --- | --- |
| Paclitaxel | Sites will use paclitaxel from pharmacy stock and manufacturers include Accord Healthcare Ltd, Hospira UK Ltd, Actavis UK Ltd, medac GmbH.  Irrespective of the brand used at site, for the RSI the SmPC from Accord Healthcare Ltd for paclitaxel 6 mg/ml concentrate for solution for infusion will be used. | Section 4.8. |
| Cediranib | IB  Manufacturer: AstraZeneca | Section 5.6 |
| Olaparib | IB  Manufacturer: AstraZeneca  Manufacturer: AbbVie Deutschland GmbH & Co | Section 5.6 |

Table 12: Location of RSI for each IMP.

## Reporting procedures

### Participating site responsibilities

The PI (or delegated medically qualified doctor from the trial team) should sign and date the SAE CRF to acknowledge that he/she has performed the seriousness and causality assessments. Investigators should also report SAEs to their own health boards or trust in accordance with local practice.

A completed SAE form for all events requiring immediate reporting should be submitted via fax or email to the CTR within 24 hours of knowledge of the event. A separate form must be used to report each event, irrespective of whether or not the events had the same date of onset.

| SAEs must be reported to the CTR Safety Team—see section 0 for contact details |
| --- |

The participant will be identified only by trial number, month and year of birth, and initials. The participant’s name must not be used on any correspondence.

It is required that sites respond to and clarify any queries raised on any reported SAEs and report any additional information as and when it becomes available through to the resolution of the event. Additionally, CTR/pharmaceutical companies may request additional information relating to any SAEs/SARs and the site should provide as much information as is available to them in order to resolve these queries.

SAEs should be reported from randomisation, throughout the treatment period and until:

- the end of follow-up for any of these events: development of MDS, AML or other clonal blood disorder; pneumonitis grade 2 or worse; new malignancies; a SAR (an SAE with possible, probable or definite relationship to the IMP as determined by the investigator at site).
- 30 days after the participant receives their last dose of IMP for all other events.

All SAEs will be followed till resolved or no further information is expected.

AEs should be graded using the NCI Common Terminology Criteria for Adverse Events (CTCAE) version 4.03. The toxicity grades should be recorded on the toxicity part of the CRF.

An SAE form is not considered as complete unless the following details are provided:

• Full participant trial number

• An Adverse Event/ Adverse Reaction

• A completed assessment of the seriousness, and causality as performed by the PI (or another appropriately medically qualified doctor registered on the delegation log).

If any of these details are missing, the site will be contacted, and the information must be provided by the site to the CTR within 24 hours.

All other AEs should be reported on the CRF.

### CTR responsibilities

Following the initial report, all SAEs should be followed up to resolution wherever possible, and further information may be requested by the CTR. Follow up information must be provided on a new SAE form.

The CTR should continue reporting SAEs until 30 days after the participant receives their last dose of IMP. Serious adverse reactions, and the trial specific SAEs (section 14.2), should continue to be reported until the end of follow up.

Once an SAE is received at the CTR, it will be evaluated by staff at the CTR and sent to the CI (or their delegate) for an assessment of expectedness.

Investigator reports of suspected SARs will be reviewed immediately and those that are identified as SUSARs will be reported to the MHRA, Main Ethics Committee and AstraZeneca.

## SUSAR reporting

The University of Manchester is undertaking the duties of trial Sponsor and has delegated to the CTR the responsibility for reporting SUSARs and other SARs to the regulatory authorities (MHRA and REC) and to AstraZeneca as follows:

SUSARs which are fatal or life-threatening must be reported to the MHRA and REC within 7 calendar days of receipt at the CTR. If the report is incomplete, then additional follow-up information should be reported within a further 8 calendar days of submitting the initial report.

SUSARs that are not fatal or life-threatening must be reported to the MHRA and REC within 15 days of receipt at the CTR. Any additional, relevant information must be reported within a further 15 days.

N.B. There is no requirement for the CTR to report SUSARs to nIMPs to the MHRA except in the following instances:

• If the adverse reaction is suspected to be linked to an interaction between a nIMP and IMP, and is serious and unexpected, CTR should report as a SUSAR due to the interaction with the IMP.

• If a SUSAR is suspected and might be linked to either a nIMP or an IMP and cannot be attributed to only one of these.

• If the adverse reaction due to the nIMP is likely to affect the safety of trial subjects then an USM may be required (see section 14.10).

## Unblinding for the purposes of SUSAR reporting

This is an open-label study and therefore unblinding is not required.

## Safety Reports

A list of all SARs (expected and unexpected) will be reported annually to the MHRA, REC, Sponsor and AstraZeneca in the form of a Development Safety Update Report (DSUR).

The CTR will report a list of all SARs (expected and unexpected) and any other safety recommendations to all PIs annually throughout the course of the trial. This frequency may be reviewed and amended as necessary. This reporting will be done via an Investigator Safety Report (ISR).

AstraZeneca will provide periodic safety reports to the Sponsor. The CTR safety team will advise AstraZeneca by email of all SAEs within 1 business day of the CTR becoming aware of the SAE. If required, the CTR will send copies of all SAE forms to AstraZeneca. The CTR safety team will also keep AstraZeneca informed of the status of SAEs with update emails and will provide full details of how the SAE has resolved.

## **Contraception and pregnancy**

Most women participating in this trial will have previously been surgically treated with a hysterectomy and will not be fertile. In addition, most women are likely to be aged over 60 years and post-menopausal. Any woman who is already pregnant is not eligible to take part in the trial.

After review of the participant’s medical history the PI will decide if the participant is of child-bearing potential.

If the participant is of child-bearing potential, then the PI will discuss with the participant the trial requirements for contraceptive use, the potential for harm to a developing foetus by the IMP, and the requirements to report and follow-up pregnancies. Participants of child-bearing potential will be required to take a pregnancy test before starting trial treatment, monthly during the treatment period, and at the end-of-treatment assessment.

If the participant is not of child-bearing potential, then it is not necessary for the PI to raise these issues with the participant.

### Contraception

If a participant is of childbearing potential, then for inclusion in the trial they must agree to use at least one highly effective birth control method throughout the treatment period and for six months following the end of treatment. For the list of acceptable contraceptive methods see the exclusion criteria in section 8.2.

### Pregnancy reporting whilst participating in the trial

Pregnancy whilst participating in the trial is not considered an SAE, however, a congenital anomaly or birth defect is. When pregnancy occurs, this will be followed up until the end of pregnancy, so it is known if a congenital anomaly or birth defect occurred that therefore requires SAE reporting. Sites will report any pregnancy that occurs within six months of the end of treatment.

The CTR has a standard procedure for pregnancy reporting which will be followed in the unlikely event of a pregnancy occurring.

## Urgent Safety Measures (USMs)

An urgent safety measure is an action that the Sponsor, CI or PI may carry out in order to protect the participants of a trial against any immediate hazard to their health or safety. If the USM has been actioned by the PI, then this must be communicated to the CTR **immediately** via the CTR Safety Team (see contact details in section 0).

The CTR will then notify the MHRA of any urgent safety measure relating to this trial immediately by telephone, and then within 3 days in writing to both the MHRA and REC, that such a measure has been taken. CTR will handle reported USMs according to CTR processes.

# Statistical considerations

## Randomisation

Participants will be registered and randomised for the trial as described in section 9.5.

## Blinding

There are no blinding or unblinding requirements as this is an open-label trial. It is not pragmatic for this study to be blinded due to both the different drug delivery methods between arms (infusion and oral), and the well-known and distinctive side-effects of the trial drugs.

## Sample size

A Multi-Arm Multi-Stage (MAMS) framework has been adopted, with a relaxed type 1 error reflective of the phase 2 setting and a 3-arm 2-stage design (Bratton et al, 2013). For the control arm, PFS at 3 months is anticipated to be approximately 50 % (Fleming et al 2015; Oza et al 2015) and the trial is powered for an improvement in either test arm to 70 %. The design is first derived using normal approximations and the MAMS sample size program within Stata was used; the interim analysis is to have a nominal alpha of 0.50 and power of 90 %, whereas the final analysis will have a nominal alpha of 0.20 and power of 85 %. These criteria yield a design with an interim analysis after 19 participants in each arm (57 in total) have 3-month RECIST data available, which we estimate to be after 16 months of starting recruitment, and a final analysis after 41 participants in each arm (123 in total) have been obtained. Allowing for 5 % lost to follow-up from dropouts and from participants being non-evaluable, a total of 129 participants will be recruited, so that the interim analysis will be conducted after 20 participants in each arm (60 in total) have 3-month RECIST data available.

## Missing, unused & spurious data

There will be no data imputation for missing data in the primary endpoint. Imputation methods may be proposed for purposes of sensitivity analysis—imputation methods for missing data in the primary endpoint and secondary endpoints will be fully documented in the SAP.

If a participant has missing primary outcome data at the 3 month time point, then data confirming PFS at later time points will be used to assume PFS at 3 months. If progression has been recorded at the next time point, then the 3 month data will be unevaluable.

Time to event data will be censored at the date the participant was last seen if no event is recorded during the trial.

The EORTC QLQ30 and EN24 module allow for some items to be calculated if at least 50 % of responses have been provided.

## Procedures for reporting deviation(s) from the original SAP

Deviations from the original SAP will be submitted as substantial amendments where applicable and recorded in subsequent versions of the Protocol and SAP. Any deviation(s) from the final statistical plan will be described and justification given in the final report.

## Termination of the trial

Decisions on the termination of the trial are the responsibility of the TSC with guidance from the IDMC (see section 23).

Safety will be assessed by the IDMC according to the timeframe in section 23.3.

If the interim analysis shows one of the experimental arms is not significant at the nominal alpha level, then the IDMC may recommend that the trial arm should be dropped, or if both experimental arms are not significant at this stage, then the IDMC may recommend the trial be closed early.

## Inclusion in analysis

The Intention-to-Treat (ITT) population will be the basis for the primary analysis of efficacy in this study and will constitute all randomised participants. Participants in the ITT population will be included in the treatment arm to which they were randomised.

# Analysis

A Statistical Analysis Plan (SAP) will be developed before the first interim analysis of the trial.

Histograms and boxplots will be used to check the distribution and possible outliers for continuous variables. Continuous variables that follow a normal distribution will be summarised using means and standard deviations. Skewed continuous variables will be summarised using medians and inter-quartile ranges. Categorical variables will be summarised using frequencies and percentages. Univariate models will be performed for all demographic and other study variables, as statistically appropriate, with models such as Fisher’s exact test or the Chi-square test utilised for categorical variables and t-tests or the Wilcoxon test (if the continuous variable does not follow a normal distribution) for continuous ones.

## Main analysis

### Safety Analysis:

Safety analysis will include all participants who received study treatment. Analyses will consist of data summaries for clinical and laboratory parameters, and for AEs. The safety data will be summarized by treatment arm. Vital signs and ECG data will be summarised by changes from baseline values using descriptive statistics. Laboratory parameters will also be summarised using descriptive statistics. The frequency of adverse events (AEs) will be assessed for severity (CTCAE version 4.03), expectedness, seriousness and causal relationship to study drugs(s). In addition, AEs will be summarised by toxicity type, impact on study drug(s) and by timing. AEs will be coded using Medical Dictionary for Regulatory Activities (MedDRA) terminology.

### Efficacy Analysis:

The Intention-to-Treat (ITT) population will be the basis for the primary analysis of efficacy in this study and will constitute all randomised subjects. Subjects in the ITT population will be included in the treatment arm to which they were randomised.

The primary outcome is PFS at 3 months, according to RECIST v1.1 criteria. In each trial arm, the proportion of participants who are progression free at the 3 month scan will be calculated from the total number of participants randomised. The same endpoint of PFS at 3 months will be used to assess the trial at the interim analysis (see section 16.1.5). The difference in proportions between each experimental trial arm and the control arm will be calculated and assessed using a two sample test of proportions. One-tail tests will be considered for each test arm against control. Six months PFS will be similarly calculated as a secondary endpoint. PFS will be summarized descriptively using the Kaplan-Meier method in both arms of the trial. Median PFS will be estimated for each arm of the trial, as a secondary endpoint, from the 50th percentile of the corresponding Kaplan-Meier estimates and then the one-sided logrank test will be then used to formally test the equality of the survivor functions.

Further, treatment groups will be compared using the log-rank test stratified by the randomisation stratification factors. The hazard ratio will be estimated using a stratified Cox proportional hazards model, i.e. assuming different baseline hazards for each stratum. Results based on unstratified analyses also will be presented for sensitivity analysis. If the hazards are proportional, then Cox regression will be performed to adjust the hazard ratio for the stratification factors. Similar analyses will be performed for the secondary endpoint of OS especially median OS. The radiological response rate assessed by RECIST v1.1 will be compared using the Cochran-Mantel Haenszel chi square test. For all estimates, 95 % confidence intervals will be calculated.

Exploratory subgroup analyses will also be conducted on the stratification variables, namely number of chemotherapy regimens for metastatic disease and cancer type.

### Exploratory Analysis:

The translational research endpoints include measuring changes in plasma concentration of circulating angiogenesis-related cytokines, measuring changes in concentration of CTCs and evaluation of *POLEm*, MMRd, p53abn and NSMP molecular groups. Exact analyses will be summarised in a separate translational protocol and approved by the TSC.

### Quality of life Analysis:

QL will be measured by EORTC QLQ-C30 questionnaire and the endometrial specific subscale EN28. These scales will be scored by first estimating a raw score, followed by using a linear transformation to standardize the raw score, so that the scores range from 0 to 100. Codes for the scoring procedure provided by EORTC will be utilized. Missing item data will be treated as recommended by EORTC, namely by determining whether items are missing for a particular reason and then utilizing their proposed method of imputation. In the case of missing form data, the investigators will document and report the reasons and extent of the missing data.

### Interim analysis

There will be two planned analyses; an interim analysis with the potential early termination of either test arm for lack of benefit after 20 participants in each arm (60 in total) have 3 month RECIST data available (which we estimate to be after 16 months of starting recruitment), and a final analysis after 43 participants in each arm (129 in total) have completed participation in the trial.

Recruitment will continue whilst awaiting the outcome of interim analysis.

# Data Management

Source Data is defined as “*All information in original records and certified copies of original records of clinical findings, observations or other activities in a clinical trial necessary for the reconstruction and evaluation of the trial. Source data are contained in source documents.*” There is only one set of source data for any data element.

Source documents include, but are not limited to, hospital records (from which medical history and previous and concurrent medication may be summarised into the CRF), clinical and office charts, laboratory and pharmacy records, diaries, microfiches, radiographs, and correspondence. CRF entries will be considered source data if the CRF is the site of the original recording (e.g., there is no other written or electronic record of data). All documents will be stored safely in confidential conditions. Sites will retain all original source data from these investigations for future reference. On all trial-specific documents, other than the signed consent form, the participant will be referred to by the trial participant ID, not by name.

| Trial data | Source data  (* may be any of these sources depending on local procedures) | | | | | | |
| --- | --- | --- | --- | --- | --- | --- | --- |
|  | Consent form | CRF | Medical notes / GP letter | Pharmacy file | Questionnaire | NHS reports | Laboratory data |
| Written informed consent | X |  |  |  |  |  |  |
| Written informed consent for optional samples and archived samples | X |  |  |  |  |  |  |
| Medical history |  |  | X |  |  |  |  |
| Pregnancy test |  | X* |  |  |  | X* |  |
| ECOG Performance status |  | X |  |  |  |  |  |
| Physical Examination |  | X |  |  |  |  |  |
| Vital signs – blood pressure and temperature |  | X |  |  |  |  |  |
| Urine for protein |  | X* |  |  |  | X* |  |
| Safety bloods |  |  |  |  |  | X |  |
| Full Blood Count |  |  |  |  |  | X |  |
| RECIST v1.1 reporting |  |  |  |  |  | X |  |
| Adverse event and toxicity evaluation |  | X* | X* |  |  | X* |  |
| QL |  |  |  |  | X |  |  |
| Concomitant treatments/medications |  |  | X |  |  |  |  |
| Overall survival |  |  | X |  |  |  |  |
| ECG |  |  |  |  |  | X |  |
| Translational sample results |  |  |  |  |  |  | X |
| Trial medication dispensed |  |  |  | X |  |  |  |

Table 13: Source data for the COPELIA trial

## Completion of Paper CRFs

A paper CRF will be used to collate the trial data. Participating sites will be provided with training and instructions on how to complete and return the CRFs.

A copy of each completed CRF will be returned to the CTR for data entry, and one copy will be retained at the local site. In accordance with the principles of GCP, the PI is responsible for ensuring accuracy, completeness, legibility and timeliness of the data reported to the CTR in the CRFs.

CRF pages and data received by the CTR from participating trial sites will be checked for missing, illegible or unusual values (range checks) and consistency over time. If missing or questionable data are identified, a data query will be raised with the site. Sites will be instructed on the procedure to answer queries.

The CRF pages should not be altered. All answered data queries and corrections should be signed off and dated by a delegated member of staff at the relevant participating site. The completed data clarification form should be returned to the CTR and a copy retained at the site along with the participants’ CRF.

The CTR will send reminders for any overdue data. It is the site’s responsibility to submit complete and accurate data in timely manner.

# Translational research

Samples for translational research will only be taken from participants who have consented to this optional part of the study. The translational research in this trial will cover two areas. These are:

1. Soluble angiogenesis-related biomarkers: Published data have shown that VEGF pathway inhibitors, such as cediranib, reduce the plasma concentration of VEGFR2, whereas at the development of progression, perturbation of the Ang-Tie axis is observed (Batchelor et al 2007; Valle et al 2015). Therefore, for all trial arms, at screening, at day 1 of cycles 1-3 of treatment, and upon development of progressive disease, plasma samples will be taken according to the laboratory manual and sent to the Cancer Research UK Manchester Institute, where they will be analysed in batches to determine plasma levels of VEGF-A, VEGFR2, Ang1, Ang2 and Tie2.
2. Circulating Tumour Cells: CTC levels have been demonstrated to have prognostic significance in many epithelial malignancies and have been proposed as non-invasive means for real-time patient monitoring and treatment stratification (Krebs et al 2014). Our unpublished data (Kristeleit et al, UCL) demonstrate that 55 % of patients with advanced endometrial cancer have detectable CTCs and we propose to assess serial CTC levels in this subgroup.

Details of timepoints for translational research sampling, and instructions for sample handling, processing and shipping can be found in the COPELIA sample handling manual.

After the planned analysis, some of the donated samples may be left over. These samples are incredibly valuable to us and all participants who have opted to gift samples will have consented to future testing, including genetic testing. Samples will always be stored, processed or destroyed in accordance with legal and regulatory requirements.

The PIS makes it clear to participants that we cannot predict all possible uses of the samples in the future and that they will not be informed, or asked for permission, about any future tests. Participants are also informed that after the trial testing any remaining samples will be anonymised and it will therefore no longer be possible for participants to request their samples be destroyed. With the agreement of the TMG, anonymised samples, with some non-identifying information such as the participant’s age, may be released for further analysis to researchers from other universities or commercial companies.

During the consent process participants can also opt to donate archived tissue samples collected before starting in the trial. This consent is being obtained to allow us to evaluate putative biomarkers of benefit or resistance in tumour tissue that may become of interest during or following the trial. Archived tissue samples will only be requested with the permission of the TMG and if appropriate funding and approvals are in place for the planned testing. Participants will not be informed, or asked for permission, about any future tests on archived tissue.

# Protocol/GCP non-compliance

PIs will report any non-compliance to the trial protocol, or the conditions and principles of GCP, to the CTR in writing as soon as they become aware of it. The CTR will advise the PI what information and actions are required.

The CTR will follow its standard procedures to ensure non-compliances are correctly classified (in terms of seriousness), documented and reported as required. A corrective and preventative action plan may be implemented by the CTR.

The CTR is responsible for reporting serious breaches.

If an urgent safety measure has been taken to protect a participant(s) from immediate harm, then see section 14.10.

# End of Trial definition

The end of the trial is defined as the date of final data capture to meet trial endpoints. The trial will end once all participants have met at least one of the criteria: completed 12 months’ follow-up (including treatment), withdrawn from follow-up, been lost to follow-up, experienced disease progression, or died.

On behalf of the Sponsor, the CTR will notify the MHRA and main REC of the end of the trial within 90 days of its completion, or within 15 days if the trial is terminated early.

# Archiving

The TMF will be archived at an approved external storage facility for a minimum of 25 years. The Sponsor is responsible for archiving the TMF. Each PI is responsible for archival of their ISF on approval from the Sponsor. Essential documents pertaining to the trial shall not be destroyed without permission from the Sponsor.

# Regulatory Considerations

Decisions to amend the trial protocol will be made by the TMG. It is the responsibility of the Sponsor to determine whether an amendment is substantial. Amendment applications will be submitted to the MHRA, REC and HRA in accordance with their procedures. Version control and communication of amendments to stakeholders will be done in accordance with CTR procedures.

## CTA

This trial has Clinical Trials Authorisation (CTA) from the UK Competent Authority: MHRA.

## Ethical and governance approval

This protocol has approval from a Research Ethics Committee (REC) that is legally “recognised” by the United Kingdom Ethics Committee Authority for review and approval.

This trial protocol will be submitted through the HRA which assesses governance and legal compliance for the NHS in England. The HRA approval process replaces the need for local checks of legal compliance and related matters by participating sites in England—this means sites do not give site specific governance approval. If additional governance review and approval (at local or national level) is required to open sites in Wales or Scotland then this will be obtained (note, the national administrations are working to develop a UK-wide review system, but this may not be in place for the start of the trial).

Participating sites will confirm their capacity and capability to deliver the study.

## Data protection and participant confidentiality

Once randomised, participants will be assigned a unique trial ID which will be used, in addition to their initials and month and year of birth, throughout their participation in the trial to link trial data. All staff involved with the trial will comply with the requirements of the Data Protection Act 2018. The CTR is the data custodian. Representatives of the Sponsor, CTR or regulatory authorities will be given access to trial data and trial documents (at sites or the CTR) for monitoring or inspection purposes. Prior written agreement from the Sponsor or its designee must be obtained for the disclosure of any confidential information to other parties.

### Data at sites

Sites will ensure all trial data and trial documents are stored securely. All digital data will be stored on password protected computers. Sites are responsible for ensuring the participant’s confidentiality is maintained at all times. Sites will not pass any participant-identifying data (e.g., name, address) into the public domain.

### Data at the CTR

With the exception of participant’s initials and month and year of birth recorded on CRFs, the CTR will not request sites to send any participant-identifying data and the CTR will not store participant-identifying data. Any participant-identifying data received by the CTR in error will be redacted or destroyed, and the sender notified.

CRFs containing trial data will be sent to the CTR and these will be stored in a secure and accessible manner at the CTR, with the possibility of archiving securely offsite if the need arises. All digital data will be stored on password protected computers on a secure computer network with frequent back-ups to prevent data loss. Data stored on other media will be encrypted.

## Indemnity and Sponsorship

The University of Manchester will act as the sponsor for this trial. Delegated responsibilities will be assigned to the CTR to manage the trial on behalf of the Sponsor, and to participating sites recruiting participants into this trial. All participants will be recruited at NHS sites and therefore the NHS indemnity scheme/NHS professional indemnity will apply with respect to claims arising from harm to participants at site.

The University of Manchester has a specialist insurance policy in place for research involving human participants (e.g., clinical trials) that provides cover for legal liabilities arising from its actions or those of its staff or supervised students, subject to policy terms and conditions.

AstraZeneca have accepted limited liability relating to the manufacturing and original packaging of the trial drug and to the losses, damages, claims or liabilities incurred by trial participants based on known or unknown AEs which arise out of the manufacturing and original packaging of the trial drug, but not where there is any modification to the trial drug (including without limitation re-packaging and blinding).

## Funding

The COPELIA trial is being funded by AstraZeneca.

# Trial committees

## TMG (Trial Management Group)

The Trial Management Group (TMG) will include those individuals responsible for the day-to-day management of the trial including at least the CIs, co-investigators and identified collaborators, the trial statistician and the trial manager(s). It will also include at least one patient representative. TMG members will be required to sign up to the remit and conditions as set out in the TMG Charter.

Notwithstanding the legal obligations of the Sponsor and CIs, the TMG have operational responsibility for the conduct of the trial including monitoring overall progress to ensure the protocol is adhered to and to take appropriate action to safeguard the participants and the quality of the trial.

The TMG will meet at least as frequently as required by the CTR SOP on TMGs. The current SOP requires a meeting at least every 3 months, but this frequency may change if the SOP changes. Details of significant and relevant issues will be circulated to PIs as requested by the TMG. Minutes from any TMG meetings will be provided to PIs on request.

The Committee’s terms of reference, roles and responsibilities will be defined in a charter.

## TSC (Trial Steering Committee)

The Trial Steering Committee (TSC) will be facilitated by the CTR. The CTR organises regular TSC meetings which oversee a number of phase II trials being managed by the CTR. The appointed Chairperson will be independent from the trial (not involved directly in the trial other than as a member of the TSC). Non-independent members include one CI, the TM and trial statistician. It is important for the independence of the committee that the majority of committee members are independent of the trial. TSC members will be required to sign up to the remit and conditions as set out in the TSC Charter. Additional observers may be present at the TSC without voting rights and may be asked to leave the meeting by the Chairperson.

The purpose of the TSC is to be an independent committee that takes responsibility for the scientific integrity of the trial, the scientific validity of the trial protocol, assessment of the trial quality and conduct (to ensure that the trial is being conducted in accordance with the principles of GCP and the relevant regulations) as well as for the scientific quality of the final trial report. Decisions about the continuation or termination of the trial are the responsibility of the TSC.

The TSC will meet once ethics approval has been given and before the trial begins recruitment. Once the trial has started the TSC will meet at least annually to monitor the progress of the trial, although there may be periods when more frequent meetings are necessary.

The Committee’s terms of reference, roles and responsibilities will be defined in a charter.

## Independent Data Monitoring Committee (IDMC)

The IDMC will be independent of the investigators, funder and Sponsor and will comprise of an independent statistician and at least two other independent experts in gynaecological cancers. IDMC members will be required to sign up to the remit and conditions as set out in the IDMC Charter.

The IDMC will review accruing trial data and assess whether there are any safety issues that need to be addressed, or if there are any reasons to terminate the trial. Reports to the IDMC will be prepared and presented by the trial statistician prior to the IDMC meeting. The trial statistician may be called in to the IDMC meeting to answer questions, and the IDMC may request additional reports or information. The IDMC Chairperson will report the IDMC recommendations to the TSC. The report may also be submitted to the TMG and if required, the REC and MHRA.

To allow an early assessment of any possible safety issues, the IDMC will meet once eight participants have completed one cycle of treatment in each arm. The IDMC will then meet at least annually, although there may be periods when more frequent meetings are necessary.

The Committee’s terms of reference, roles and responsibilities will be defined in a charter.

# Quality Control and Assurance

## Monitoring

The clinical trial risk assessment will be used to determine the intensity and focus of central and on-site monitoring activity in the COPELIA trial. A trial monitoring plan will be in place and fully documented before the trial opens to recruitment.

The monitoring plan will be developed in accordance with the MRC/DH/MHRA Joint project guidance document ‘Risk-adapted approaches to the management of Clinical Trials of Investigational Medicinal Products’. Due to the Type C risk associated with the IMP (see section 6.1), a higher intensity monitoring plan will be employed, with additional monitoring if required to address specific vulnerabilities identified in the risk assessment.

Site investigators will permit trial related monitoring by providing direct access to source data and documents as required. Participant consent for this will be obtained.

Findings generated from on-site and central monitoring will be shared with the Sponsor, CI, PIs and local R&D departments.

## Audits & inspections

The trial is participant to inspection by the MHRA as the regulatory body. The trial may also be participant to inspection and audit by the University of Manchester under their remit as Sponsor, and the CTR under their delegated duties in managing the trial.

The CI, or PIs and participating sites, will permit audits, REC review, and regulatory inspections, providing direct access to source data and documents. Participating sites must inform the CTR of any MHRA inspections.

# Publication policy

All presentations and publications relating to the trial will be authorised by the TMG and Sponsor.

The main trial results will be published in the name of the trial in a peer-reviewed journal, on behalf of all collaborators. The manuscript will be prepared by a writing group, appointed from amongst the Trial Management Group, and this may also include high accruing clinicians and/or other people who contribute to the trial. All participating centres and clinicians will be acknowledged in this main publication together with appropriate staff from the CTR.

Authorship of any secondary publications, e.g., relating to the various biological studies, will reflect the intellectual and scientific input of individuals into these studies, and will not necessarily be the same as on the primary publication.

# References

Abkevich V et al. Patterns of genomic loss of heterozygosity predict homologous recombination repair defects in epithelial ovarian cancer. Br J Cancer 1776-1782 (2012)

Aghajanian C et al. Phase II trial of bevacizumab in recurrent or persistent endometrial cancer. J Clin Oncol 2259-65 (2011)

Aparna AK et al. Metronomic chemotherapy enhances the efficacy of antivascular therapy in ovarian cancer. Cancer Res 67: 281-288 (2007)

Audeh MW et al. Oral poly (ADP-ribose) polymerase inhibitor olaparib in patients with BRCA1 or BRCA2 mutations and recurrent ovarian cancer: a proof-of-concept trial. Lancet 245-51 (2011)

Backen AC et al. 'Fit-for-purpose' validation of SearchLight multiplex ELISAs of angiogenesis for clinical trial use. J Immunol Methods 106-114 (2009)

Batchelor TT et al. AZD2171, a Pan-VEGF Receptor Tyrosine Kinase Inhibitor, Normalizes Tumor Vasculature and Alleviates Edema in Glioblastoma Patients. Cancer Cell 11: 83-95 (2007)

Bender D et al. A phase II evaluation of cediranib in the treatment of recurrent or persistent endometrial cancer. Gynecol Oncol 507-12 (2015)

Bilbao C et al. Double strand break repair components are frequent targets for microsatellite instability in endometrial cancer. Eur J Cancer 2821-7 (2010)

Bratton DJ, Phillips PP, Parmar MK. A multi-arm multi-stage clinical trial design for binary outcomes with application to tuberculosis. BMC Med Res Methodol 2013; 13(1):139

Bryant HE et al. Specific killing of BRCA2-deficient tumours with inhibitors of poly (ADP-ribose) polymerase. Nature 913-7 (2005)

Castonguay V et al. A phase II trial of sunitinib in women with metastatic or recurrent endometrial carcinoma. Gynecol Oncol 274-80 (2014)

Cerbinskaite A et al Defective homologous recombination in human cancers. Cancer Treatment Reviews 89-100 (2012)

Curtin NJ et al DNA repair dysregulation from cancer driver to therapeutic target. Nature Reviews Cancer. 801-17 (2012)

Daemen A et al Cross-platform pathway-based analysis identifies markers of response to the PARP inhibitor olaparib. Breast Cancer Research and Treatment. 505-17 (2012)

Dedes KJ et al Synthetic lethality of PARP inhibition in cancers lacking BRCA1 and BRCA2 mutations. Cell Cycle. 1192-9 (2011)

Dedes KJ et al PTEN deficiency in endometrioid endometrial adenocarcinomas predicts sensitivity to PARP inhibitors. Sci Transl Med 53ra75 (2010)

Drew Y et al Therapeutic potential of PARP inhibitor AG014699 in human cancer with mutated or methylated BRCA. JNCI 334-346 (2011)

Drew Y et al. Therapeutic potential of poly (ADP-ribose) polymerase inhibitor AG014699 in human cancers with mutated or methylated BRCA1 or BRCA2. Journal of the National Cancer Institute.334-46 (2011)

Fleming GF et al. Phase III trial of doxorubicin plus cisplatin with or without paclitaxel plus filgrastim in advanced endometrial carcinoma: a Gynecologic Oncology Group Trial. Journal of Clinical Oncology. 2159-66 (2004)

Fleming GF et al Second-line therapy for endometrial cancer: The need for better options. J Clin Oncol 3535-40 (2015)

Forster M et al Treatment with Olaparib in a patient with PTEN-deficient endometrioid endometrial cancer. Nat Rev Clin Oncol 302-306 (2011)

Hecht JL et al. Molecular and pathologic aspects of endometrial carcinogenesis. Journal of Clinical Oncology. 4783-91 (2006)

Hirte H et al. A phase 2 trial of cediranib in recurrent or persistent ovarian, peritoneal and fallopian tube cancer. Gynecol Oncol 138:55-61 (2015)

Homesley HD et al. A phase II trial of weekly 1-hour Paclitaxel as second-line therapy for endometrial and cervical cancer. Int J Clin Oncol 13:62-65 (2008)

Kommoss et al. Final validation of the ProMisE molecular classifier for endometrial carcinoma in a large population-based case series. Ann Oncol. 29; 1180-1188 (2018).

Kamat A et al. Clinical and biological significance of VEGF in endometrial cancer. Clin Cancer Res. 7487-95 (2007)

Koppensteiner R et al. Effect of MRE11 loss on PARP-inhibitor sensitivity in endometrial cancer in vitro. PLoS One 13; 9(6) (2014)

Konstantinopoulos P D et al Gene Expression Profile of BRCAness That Correlates with Responsiveness to Chemotherapy and With Outcome in Patients with Epithelial Ovarian Cancer. JCO 3555 (2010)

Ledermann J et al Cediranib in patients with relapsed platinum-sensitive ovarian cancer (ICON6): a randomised, double-blind, placebo-controlled phase 3 trial. Lancet 1066-74 (2016)

Ledermann J et al Olaparib maintenance therapy in patients with platinum-sensitive relapsed serous ovarian cancer: a preplanned retrospective analysis of outcomes by BRCA status in a randomised phase 2 trial. Lancet Oncol 852-61 (2014)

Ledermann J et al Olaparib maintenance therapy in platinum-sensitive relapsed ovarian cancer. N Engl J Med 366: 1382-92 (2012)

Leon-Castillo et al Molecular Classification of the PORTEC-3 Trial for High-Risk Endometrial Cancer: Impact of Prognosis and Benefit from Adjuvant Therapy. J Clin Oncol. 38: 3388-3397 (2020)

Liu JF et al. Combination cediranib-olaparib versus olaparib alone for women with recurrent platinum-sensitive ovarian cancer:a randomised phase 2 trial. Lancet Oncol 15:1207-14 (2014)

Liu JF et al. A phase I trial of the PARP inhibitor olaparib in combination with the anti-angiogenic cediranib in recurrent epithelial ovarian or triple negative breast cancer. Eur J Cancer 2972-78 (2013)

Liu J et al. A Phase I trial optimizing the dosing of olaparib tablet formulation combined with cediranib in recurrent ovarian cancer. J Clin Oncol 5559a (2015)

Lorusso D et al. Randomised phase II trial of carboplatin-paclitaxel compared to carboplatin-paclitaxel-bevacizumab in advanced (III=IV) or recurrent endometrial cancer: the MITO-END2 trial. Proc ASCO 2015

Mateo J et al. An adaptive study to determine the optimal dose of the tablet formulation of the PARP inhibitor olaparib. Targ Oncol 11: 401-415 (2016)

Matulonis U et al. Cediranib, an oral inhibitor of vascular endothelial growth factor receptor kinases, is an active drug in recurrent epithelial ovarian, fallopian tube and peritoneal cancers. J Clin Oncol 5601-06 (2009)

McConechy MK et al In-depth molecular profiling of the biphasic components of uterine carcinosarcomas. Pathol Clin Res. 173-85 (2015)

Mendes-Pereira AM et al. Synthetic lethal targeting of PTEN mutant cells with PARP inhibitors. EMBO Molecular Medicine 315-22 (2009)

Miller D et al. Late-Breaking Abstract 1: Randomized phase III noninferiority trial of first line chemotherapy for metastatic or recurrent endometrial carcinoma: A Gynecologic Oncology Group trial. Gynecologic Oncology 125, 771 (2012).

Miyasaka A et al. Anti-tumor activity of Olaparib, a poly ADP ribose polymerase (PARP) inhibitor in cultured endometrial carcinoma cells BMC Cancer 14:179 (2014)

Moskwa P et al. miR-182-mediated downregulation of BRCA1 impacts DNA repair and sensitivity to PARP inhibitors. Molecular Cell. 210-20 (2011)

Mukhopadhyay A et al Clinicopathological features of homologous recombination deficient epithelial ovarian cancers: Sensitivity to PARP inhibitors, platinum and survival. Cancer Research, 2012.

Mukhopadhyay A et al Development of a functional assay for homologous recombination status in primary cultures of epithelial ovarian tumor and correlation with sensitivity to poly (ADP-ribose) polymerase inhibitors. Clinical Cancer Research, 2344-2351 (2010)

Mukhopadhyay A et al Clinicopathological Features of Homologous Recombination-Deficient Epithelial Ovarian Cancers: Sensitivity to PARP Inhibitors, Platinum, and Survival. Cancer Research. 5675-82 (2012)

Oza et al Randomised Phase II Trial of Ridaforolimus in Advanced Endometerial Carcinoma. Journal of Clinical Oncology 33(31): 3576-82 (2015).

Pignata S et al. Pazopanib plus weekly paclitaxel versus weekly paclitaxel alone for platinum-resistant or platinum-refractory advanced ovarian cancer (MITO-11): a randomised open-label phase 2 trial. Lancet Oncol 561-568 (2015)

Powell M A et al. A phase II trial of brivanib in recurrent or persistent endometrial cancer. Gynecol Oncol 135: 38-43 (2014)

Powell MA et al Phase II evaluation of paclitaxel and carboplatin in the treatment of carcinosarcoma of the uterus: a Gynecologic Oncology Group Study J Clin Oncol 2727-31 (2010)

Pujade-Lauraine E et al. Bevacizumab combined with chemotherapy for platinum-resistant recurrent ovarian cancer. The AURELIA open-label randomized phase III trial. J Clin Oncol 1302-08 (2014)

Pujade-Lauraine E et al. Olaparib tablets as maintenance therapy in patients with platinum-sensitive, relapsed ovarian cancer and a BRCA1/2 mutation (SOLO2/ENGOT-Ov21): a double-blind, randomised, placebo-controlled, phase 3 trial. Lancet Oncol 18: 1274-84 (2017)

Rosenberg P et al. Randomized trial of single agent paclitaxel given weekly versus every three weeks and with peroral versus intravenous steroid premedication to patients with ovarian cancer previously treated with platinum. Acta Oncologica 41:5 418-424 (2002)

Salvesen HB, Haldorsen IS, Trovik J. Markers for individualised therapy in endometrial carcinoma. Lancet Oncology. 353-61 (2012)

Setiawan VW et al. Type I and II endometrial cancers: have they different risk factors? Journal of Clinical Oncology.2607-18 (2013)

Stockler M et al. Patient-reported outcome results from the open-label randomized phase III AURELIA trial evaluating bevacizumab-containing therapy for platinum-resistant recurrent ovarian cancer. J Clin Oncol 1309-16 (2014)

Symonds RP et al. Cediranib combined with carboplatin-paclitaxel in patients with metastatic or recurrent cervical cancer (CIRCCa): a randomised, double-blind placebo-controlled phase 2 trial. Lancet Oncol 1515-24 (2015)

TCGA, Integrated genomic analyses of ovarian carcinoma. Nature 609-615 (2011)

Tutt A et al Oral poly (ADP-ribose) polymerase inhibitor olaparib in patients with BRCA1 or BRCA2 mutations and advanced breast cancer: a proof-of-concept trial. Lancet. 235-4 (2010)

The Cancer Genome Atlas Research Network. Integrated genomic characterization of endometrial carcinoma. Nature 67–73 (2013)

Valle JW et al Cediranib or placebo in combination with cisplatin and gemcitabine chemotherapy for patients with advanced biliary tract cancer (ABC-03): a randomised phase 2 trial. Lancet Oncol. 16: 967-78 (2015)

Vilar E et al MRE11 deficiency increases sensitivity to poly (ADP-ribose) polymerase inhibition in microsatellite unstable colorectal cancers. Cancer Res 71: 2632-42 (2011)

Wedge SR et al. AZD2171: a highly potent, orally bioavailable, vascular endothelial growth factor receptor-2 tyrosine kinase inhibitor for the treatment of cancer. Cancer Res 65: 4389-400 (2005)

Wysham W et al BRCAness Profile of Sporadic Ovarian Cancer Predicts Disease Recurrence. PLoS ONE. 7: p. e30042 (2012)

Ying S et al Mre11-dependent degradation of stalled DNA replication forks is prevented by BRCA2 and PARP1. Cancer Research 2814-21 (2012)

# Appendices

## Appendix 1

ECOG Performance Status

0—Asymptomatic (fully active, able to carry on all pre-disease activities without restriction).

1—Symptomatic but completely ambulatory (restricted in physically strenuous activity but ambulatory and able to carry out work of a light or sedentary nature; for example, light housework, office work).

2—Symptomatic, < 50 % in bed during the day (ambulatory and capable of all self-care but unable to carry out any work activities; up and about more than 50 % of waking hours).

3—Symptomatic, > 50 % in bed, but not bedbound (capable of only limited self-care, confined to bed or chair 50 % or more of waking hours).

4—Bedbound (completely disabled, cannot carry on any self-care, totally confined to bed or chair).

5—Death.

## Appendix 2

RECIST v1.1

Objective Response Criteria (RECIST)

Complete Response (CR): Disappearance of all target and non-target lesions including normalization of elevated tumour marker level. Any pathological lymph nodes (whether target or non-target) must have reduction in short axis to < 10 mm. All non-target lymph nodes must be non-pathological in size (< 10 mm short axis).

Partial Response (PR): At least a 30 % decrease in the sum of longest diameter (LD) of target lesions taking as reference the baseline sum LD.

Stable Disease (SD): Steady state of disease; neither sufficient shrinkage to qualify for PR nor sufficient increase to qualify for PD, taking as reference the smallest sum diameters of target lesions while on study. For non-randomised studies, stable disease must be documented as present at least 4 weeks from the start of the therapy. There may be no appearance of new lesions for this category.

Progressive Disease (PD): At least a 20 % increase (and an absolute increase of at least 5 mm) in the sum of LD of measured lesions taking as references the smallest sum LD recorded since the treatment started. Appearance of new lesions will also constitute PD. In exceptional circumstances, unequivocal progression of non-target lesions may be accepted as evidence of disease progression.

Non-CR/Non-PD: Persistence of 1 or more non-target lesions and/or maintenance of tumour marker level above the normal limits.

| **Target lesions** | **Non target lesions** | **New lesions** | **Overall response** |
| --- | --- | --- | --- |
| CR | CR | No | CR |
| CR | Non-CR/Non-PD | No | PR |
| CR | Not evaluated | No | PR |
| PR | Non-PD or not all evaluated | No | PR |
| SD | Non-PD or not all evaluated | No | SD |
| Not all evaluated | Non-PD | No | NE |
| PD | Any | Yes or no | PD |
| Any | PD | Yes or no | PD |
| Any | Any | Yes | PD |
| - NE: Not evaluable - Note: participants with global deterioration of health status requiring discontinuation of treatment without objective evidence of disease progression at the time should be reported as “symptomatic deterioration”. Every effort should be made to document the objective progression even after discontinuation of treatment. | | | |

Table 14: summary of RECIST v1.1 Response Criteria.
